# Synthesis of imidazole-fused nitrogen-bridgehead heterocycles catalysed by lipase and their antifungal and antimicrobial bioactivity†

**DOI:** 10.1039/d3ra07145f

**Published:** 2024-02-07

**Authors:** Manjit Singh, Manisha Malviya, Vijay B. Yadav, Aishwarya Nikhil, Munesh Gupta

**Affiliations:** a Department of Chemistry, IIT (BHU) Varanasi India manisha.apc@itbhu.ac.in; b Department of Chemistry, University of Allahabad India; c Department of Microbiology, IMS(BHU) Varanasi India

## Abstract

An effective approach for selective C–N bond formation for synthesising imidazo[1,2-*a*] pyridine-based heterocycles using porcine pancreatic lipase (PPL) as a biocatalyst has been devised. Under moderate conditions, a series of imidazo[1,2-*a*]pyridine-based heterocycle derivatives were synthesised with remarkable selectivity in good-to-excellent yields (89–95%). Further, the antimicrobial and antifungal activities of derivatives 3ha, 3ka, 3fa, 3hc, and 3eb were observed, and they were found to be biologically active in antimicrobial susceptibility tests for Gram-positive bacteria (*Enterococcus faecalis ATCC 29212* and *Staphylococcus auris ATCC 25923*), Gram-negative bacteria (*Escherichia coli ATCC 25922* and *Pseudomonas aeruginosa ATCC 27853*) and fungal strains (*Candida albicans ATCC 90028* and *Candida tropicalis ATCC 750*).

## Abstract 2

An effective approach for selective C–N bond formation for synthesising imidazo[1,2-*a*] pyridine-based heterocycles using porcine pancreatic lipase (PPL) as a biocatalyst has been devised.

## Graphical abstract

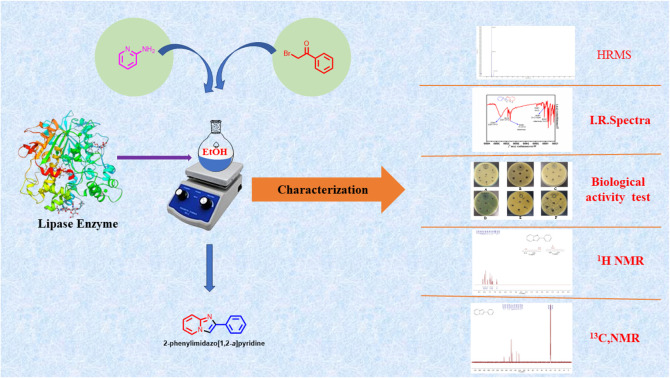

## 1. Introduction

**Fig. 1:**
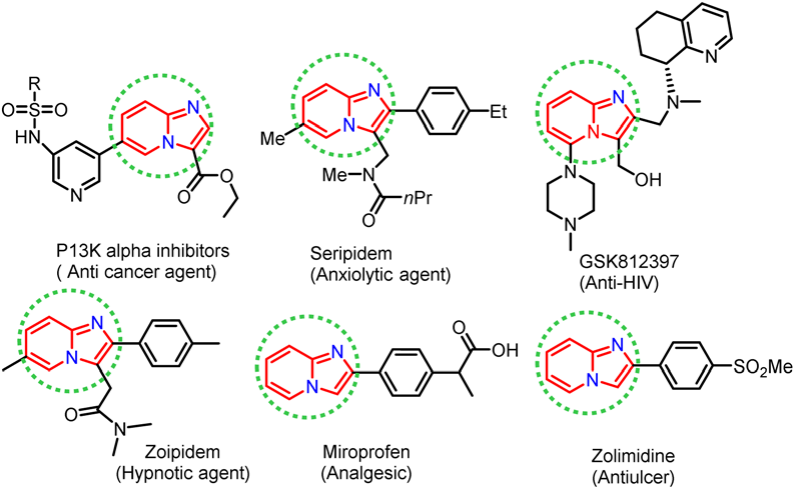
Biologically active imidazo[1,2-*a*]heterocycles.

In recent years, considerable effort has been devoted to developing new synthetic strategies using enzymatic catalysts and such strategies have received much attention, especially in synthesising heterocyclic compounds in synthetic organic chemistry.^1–5^ Lipases are pervasive enzymes that are found in all living organisms. They are essential in food, flavour, beverages, biodiesel production, and biopolymers industry. Lipase enzymes have many attractive applications, such as stability in organic solvents, catalytic activity without cofactors, broad substrate scope, and chemo-, regio- and stereo-selectivity, making them the most versatile class of enzymes in organic synthesis.^6–12^ Lipase as a catalyst has been successfully applied in nucleophilic substitution reactions, such as aldol reaction,^13^ Knoevenagel condensation,^14,15^ Mannich reaction,^16^ Henry reaction,^17^ Morita–Baylis–Hillman reaction,^18^ Michael addition,^19^ and Aza–Diels–Alder reaction.^20^ Phenyl imidazole has been shown to undergo nucleophilic substitution reactions in the presence of various catalysts, including FeCl_3_, Cu, Ag_2_CO_3_, and NBS. However, these catalysts are based on toxic metals, thus there is a need for metal-free and green biocatalysts to perform nucleophilic substitution reactions efficiently, without compromising the yield of the desired product. In this view, lipase is suitable for use in the nucleophilic substitution reaction described above and the biocatalytic properties of lipase encouraged us to exploit the development of scaffolds starting from phenacyl bromide and 2-aminopyridine as substrates. Nitrogen-containing heterocyclic rings are found in many natural products with wide-ranging biological activities. Herein, we have synthesised imidazole-fused nitrogen-bridgehead heterocycles. Imidazo[1,2-*a*]pyridines, imidazo[1,2-*a*]pyrimidines, and imidazo[1,2-*a*]pyrazines are a few essential core structures of this class that have diverse pharmacological activities.^21–26^ For example, the imidazo[1,2-*a*]pyridine moiety is found in drugs, such as alpidem,^27^ necopidem, saripidem^28^ (allanxiolytics), zolimidine^29^ (gastroprotective agent), zolpidem^30^ (hypnotic), olprinone^31^ (cardiotonic agent), GSK812397 (ref. 32) (anti-HIV) and rifaximinan,^33,34^ an antibiotic used to prevent hepatic encephalopathy. Recently, imidazopyridine molecules have also been used as anti-inflammatory, anti-ulcerative, antihypertensive, anti-bronchospastic, and antiproliferative agents (Fig. 1).^35–38^

Various methods for synthesizing this targeted moiety have been published, owing to their diverse uses. Therefore, numerous methods have been established for the synthesis of imidazo[1,2-*a*]pyridine scaffolds by the reaction of 2-aminopyridine with numerous substrates, such as methyl aryl ketone (Meng *et al.*, 2015),^39,40^ and alkyne derivatives.^41^ These reactions are generally performed in the presence of a Lewis acid and base (Scheme 2).^39–41^ Stasyuk reported the iodine-mediated synthesis of imidazo[1,2-*a*]pyridine by the reaction of 2-aminopyridine and aryl methyl ketones in the presence of a base.^42^ Synthesis of imidazo[1,2-*a*] pyridine has been reported by multicomponent reaction of 2-aminopyridines, isonitriles and aldehydes, also known as the Groebke–Blackburn–Bienayme reaction.^43–47^

**Scheme 1:**
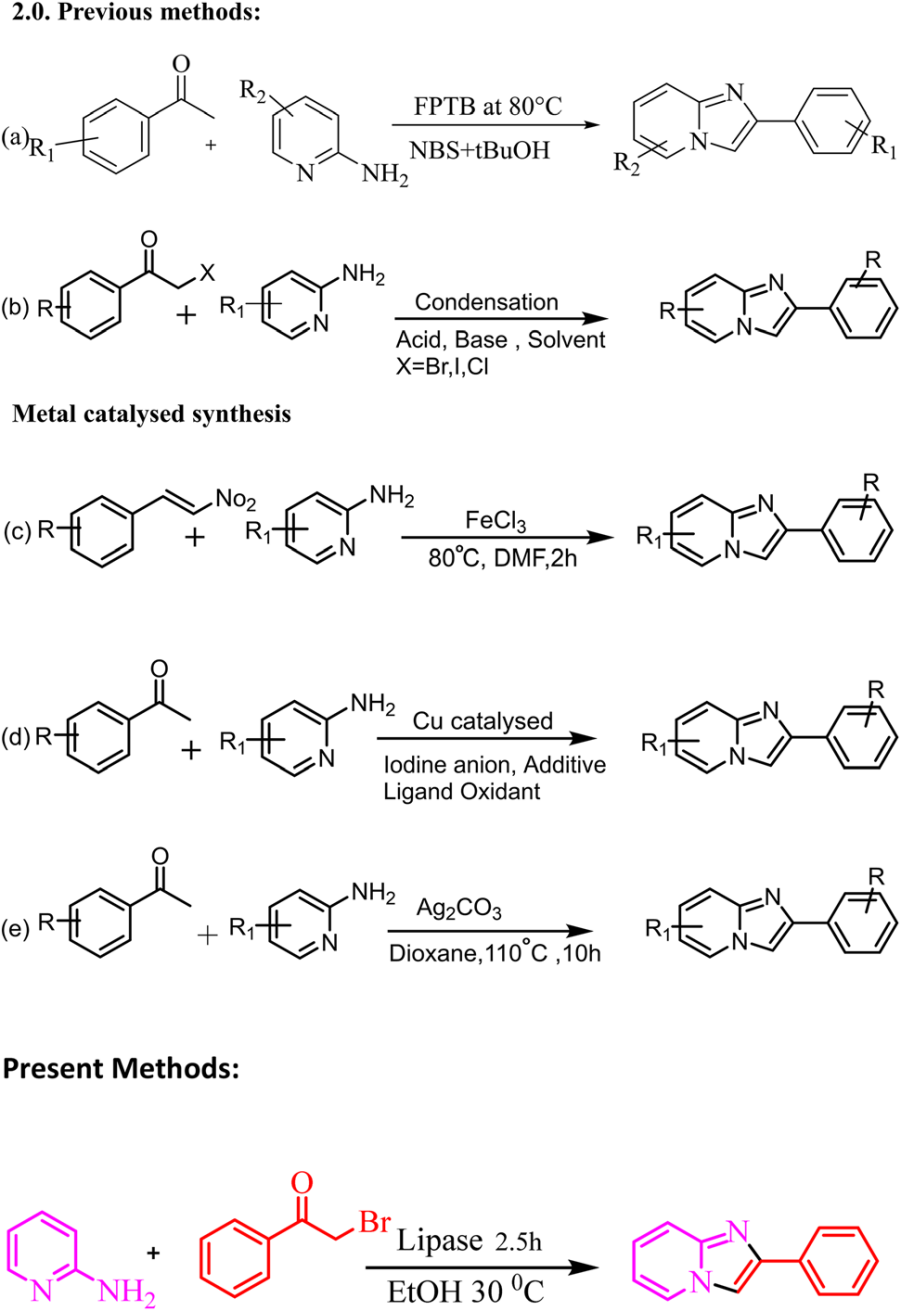
Synthesis of 2-phenyl imidazo[1,2-*a*]pyridine as a modal reaction.

These approaches are appropriate for various substrates but have shortcomings, such as acid/base, low yield, employment of severe reaction conditions, high temperatures, expensive and toxic metal catalysts, long reaction times, and time-consuming work-ups. As a result, we devised a more practical and efficient synthetic procedure: a one-pot synthesis of imidazole-fused nitrogen-bridgehead heterocycles in ethanol as the reaction medium at 30 °C utilising a biocatalyst lipase enzyme (Scheme 1). The present work describes the design, synthesis, physicochemical characterization and biological activity of 2-phenyl imidazo[1,2-*a*]pyridine. The antimicrobial and antifungal activities of derivatives 3ha, 3ka, 3fa, 3hc, and 3eb were observed, and they were found to be biologically active *via* antimicrobial susceptibility tests for Gram-positive bacteria (*Enterococcus faecalis ATCC 29212* and *Staphylococcus auris ATCC 25923*), Gram-negative bacteria (*Escherichia coli ATCC 25922* and *Pseudomonas aeruginosa ATCC 27853*) and fungal strains (*Candida albicans ATCC 90028* and *Candida tropicalis ATCC 750*).

## 2. Results and discussion

We developed an improved clean and efficient method aided one-pot synthesis of 2-aryl imidazo-fused heterocycles. To test the viability of our proposed approach, we used 2-aminopyridine (1a, 1 mmol) and phenacyl bromide (2a, 1 mmol) as substrates. Lipase is used as the catalyst, and ethanol is used as the reaction medium in this reaction.

The chemical reaction was studied using different catalysts and conditions (Table 1). For this synthesis, we first improved the catalyst system. In entries 1 and 2, the reaction proceeds without a catalyst at room temperature and 80 °C to give trace yields of products. Further, in the presence of biocatalyst system α-amylase, trypsin, and amino lipase at 30 °C, there were poor yields of the products (entry no. 3–5); diastase enzyme provides a noticeably improved product yield (entry 6). When the reaction was carried out in the presence of lipase with ethanol as the reaction medium, much to our satisfaction, the yield reached 95%. Finally, we observed that lipase was the most influential promoter regarding reaction time and yield for forming the corresponding product.

**Table 1:** Optimisation of catalyst system for the synthesis of compound 3aa^a^

Entry no.	Catalyst	Temperature (°C)	Time (h)	Yield^b^ (%)
1	No catalyst	RT	5	Trace
2	No catalyst	80	5	Trace
3	α-Amylase	30	4	36
4	Trypsin	30	4	44
5	Amino lipase	30	4	45
6	Diastase	30	4	58
7	**Lipase**	**30**	**2.5**	**95**

a Reaction conditions: all reactions were carried out with 2-2-amino pyridine (2a) (1 mmol) and phenacyl bromide (1a) (1 mmol) using ethanol as the solvent and different catalyst systems.

b Isolated yields.

Additional experiments were carried out to identify the best reaction conditions, which included altering the solvent, temperature, and catalyst quantity. Various solvents were used, including ethanol, methanol, tetrahydrofuran, dichloromethane, water, and 1,4-dioxane. Table 2 contains a summary of the findings. When the experiment was done in water, barely a trace of the product was identified. This could be due to strong hydrogen bonding effects of water, which tend to pull constitutive polar molecules from the inner structure of the enzyme, causing it to unfold and thus reducing its catalytic activity. Other polar solvents have the same effect. Meanwhile, ethanol was shown to outperform other solvents. As a result, ethanol was chosen as the best solvent for this reaction.

**Table 2:** Optimisation of solvent system for the synthesis of compound 3aa^a^

Entry no.	Solvent	Time (h)	Yield^b^ (%)
1	DMSO	5	Trace
2	Chloroform	4	Trace
3	THF	4	30
4	DMF	4	32
5	1,4-Dioxane	3	58
6	Toluene	3	40
7	Xylene	3	30
8	Benzene	3	30
9	Water	3	Trace
10	Methanol	3	58
11	**Ethanol**	**2.5**	**95**
12	Ethanol	3	95

a Reaction conditions: all reactions were carried out with 2-amino pyridine (2a) (1 mmol) and phenacyl bromide (1a) (1 mmol) using different solvent systems, and lipase was used as the catalyst.

b Isolated yields.

**Scheme 2:**
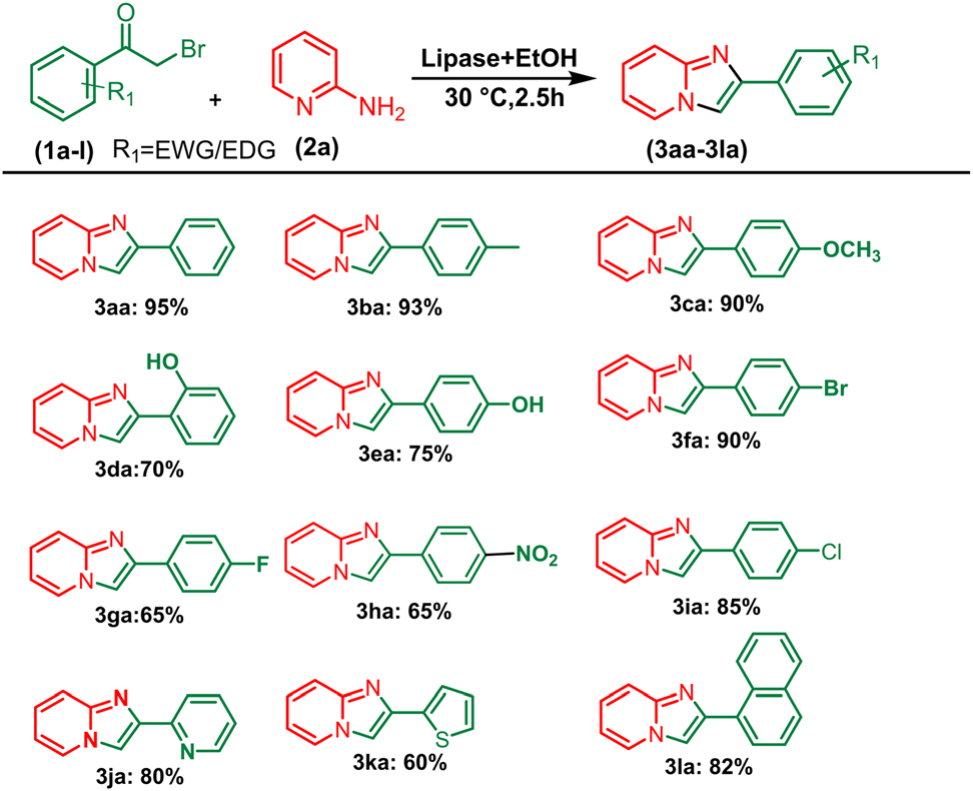
Substrate scope for synthesizing aryl series of imidazopyridines. ^*a*^Reaction conditions: alkyl methyl ketone (1 mmol) and 2-amino pyridine (2a) (1 mmol) in the presence of PPL with EtOH (3 mL) at 30 °C pyridine.

**Scheme 3:**
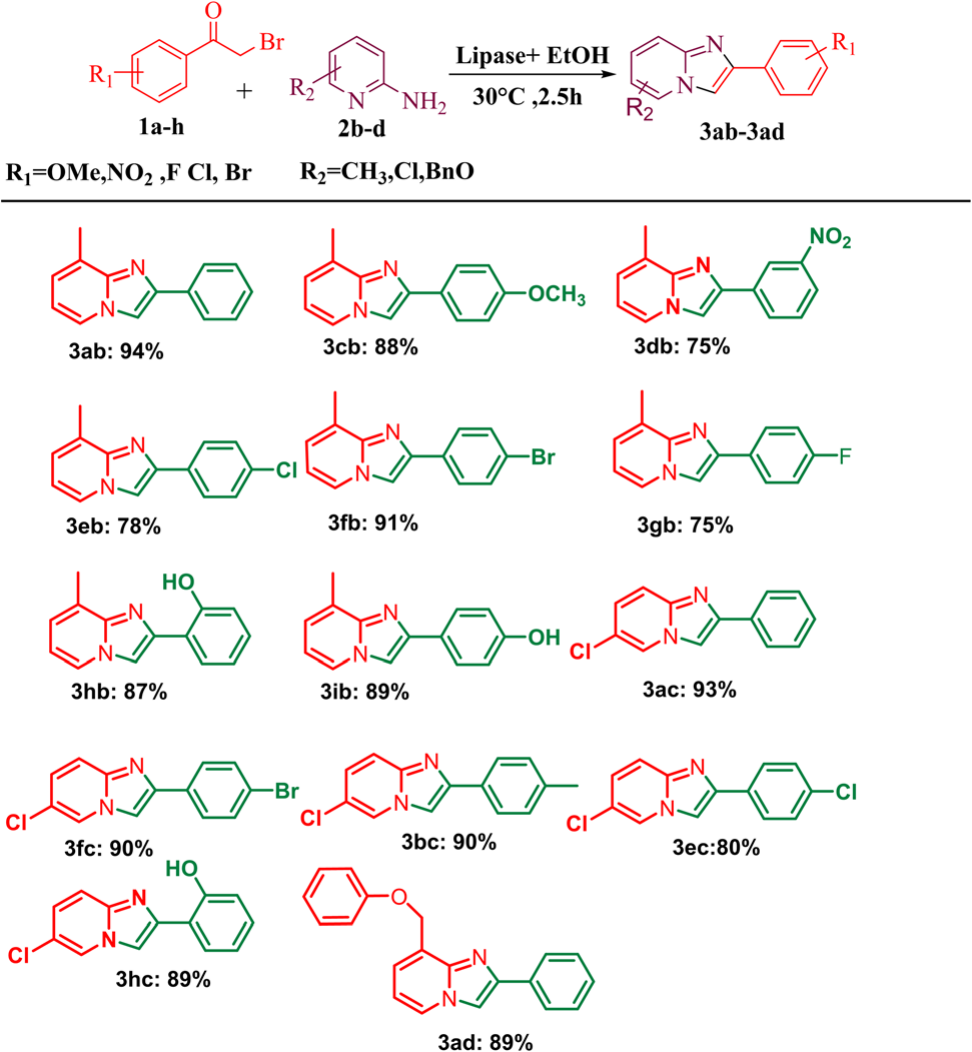
Substrate scope for one-pot conversion of imidazopyridines into corresponding 2-aminopyrimidine. ^*a*^Reaction conditions: all reactions were carried out with 2-amino pyridine (2a) (1 mmol) and phenacyl bromide (1a) (1 mmol) using the ethanol solvent system and in the presence of PPL lipase as the catalyst systems. ^*b*^Isolated yields.

**Scheme 4:**
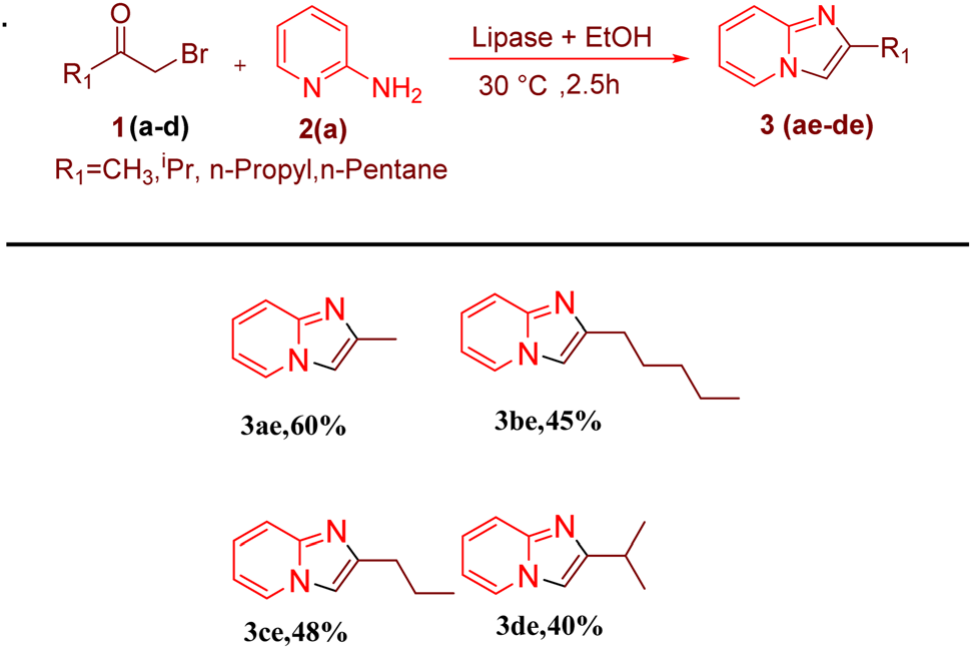
Substrate scopes for synthesizing alkyl series of imidazopyridines. Reaction conditions: alkyl methyl ketone (1 mmol) and 2-amino pyridine (2a) (1 mmol) in the presence of PPL with EtOH as the solvent (3 mL) at 30 °C.

After optimising the reaction solvent and conditions, we investigated the substrate scope for synthesizing a series of differently substituted imidazopyridines. All target molecules were successfully synthesised with good-to-excellent yields and short reaction times (Scheme 3). In general, phenacyl bromides bearing an electron-withdrawing group on the *ortho* or *para* position of the phenyl ring gave lower yields (3ca, 3fa, and 3ga). In contrast, the presence of an electron-donating group on the phenyl ring increased the yield of the desired product (Scheme 2, 3aa–3la). Meanwhile, an electron-donating group at the *ortho* or *para* position of 2-aminopyridine gave higher yields (Scheme 3, 3ab–3ib). At the same time, an electron-withdrawing group at the *ortho* or *para* position of 2-aminopyridine gave a low yield (Scheme 3, 3ac–3ad). Synthesis of imidazopyridine using an aliphatic ketone gave a low yield (Scheme 4, 3ae–3de). Conversely, aliphatic ketones are less reactive in comparison to aryl ketones, which gave only 40–60% yields of our desired products (Scheme 4, 3ab–3ab).

### 2.1. Plausible reaction mechanism

**Scheme 5:**
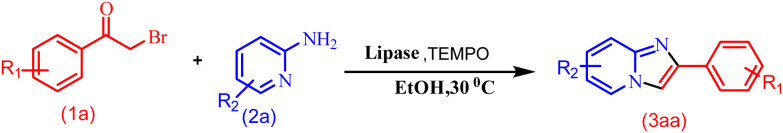
Control experiment with TEMPO.

A control experiment was conducted to explore the reaction mechanism (Scheme 5). The reaction was performed with radical scavenger TEMPO (2,2,6,6-tetramethylpiperidin-1-yloxyl) under optimised conditions, but it did not quench the reaction. This rules out the possibility of a radical pathway for the reaction.

**Scheme 6:**
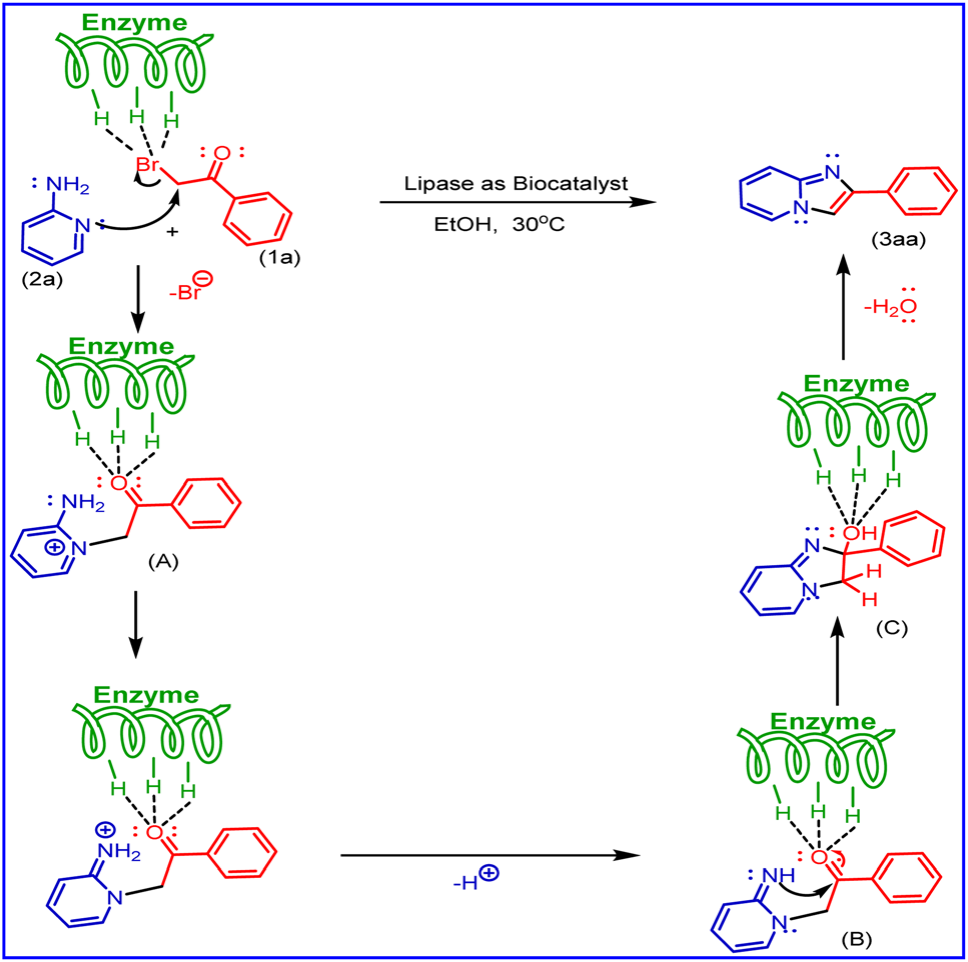
A plausible mechanism for the synthesis of 2-phenyl imidazo[1,2-*a*]pyridine derivatives.

Based on literature reports and our observations, a plausible mechanism is shown in Scheme 6. In the present methods, aminopyridine (2a) and phenacyl bromide (1a) were used as starting materials. In this, the mechanism first step undergoes Br of phenacyl bromide activated by porcine pancreatic lipase (PPL), the lone pair of aminopyridine nitrogen (ring nitrogen) attacks nucleophilic substitution phenacyl bromide to form molecule (A), also activated by (PPL) to undergo cyclisation by the attack of nitrogen (NH) lone pair (B) as nucleophilic addition on carbonyl carbon to give (C) and followed by dehydration to the formation of imidazo [1,2-*a*] pyridine derivatives (3aa).

### 2.2. Scalability of the protocol

**Scheme 7:**
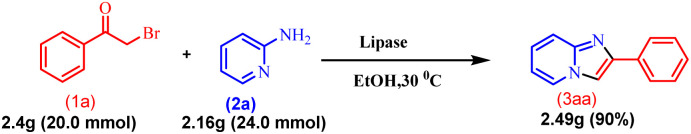
Gram-scale synthesis of imidazo[1,2-*a*]pyridine.

After developing this methodology, gram scalability was demonstrated. The reaction was performed with acetophenone (1a) (2.4 g, 20.0 mmol) and 2-amino pyridine (2a) (2.16 g, 24.0 mmol) under optimised reaction conditions, and it gave 90% (3.49 g) yield of the product (3aa), which is similar to the mmol scale synthesis. This indicates that our methodology is also effective for gram-scale synthesis (Scheme 7).

### 2.3. Reusability of the PPL lipase catalyst

**Fig. 2:**
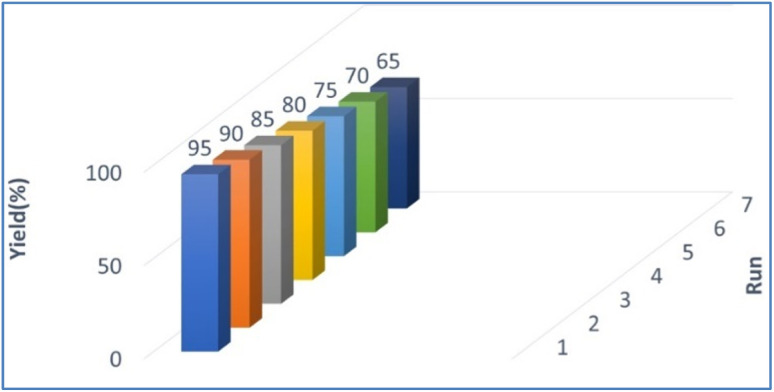
Catalyst reusability.

The reusability of PPL lipase was also examined under optimised reaction conditions for up to 7 runs (Fig. 2). The catalyst was separated by filter paper after completion of the reaction, washed with water and hexane (3 × 10 mL), dried and stored at 10 °C until used for the next reaction. The collected catalyst could be reused numerous times in the succeeding runs without a significant loss of catalytic activity. The conversion decreased after seven cycles, which may be due to the aggregation of particles of the used catalyst blocking the active sites on the catalyst.^48^

#### 2.3.1 Significance of catalyst reusability

Enzymes are very expansive, which will impact overall synthesis process costs. Therefore, enzymes that can be recycled multiple times are more desirable for industrial and laboratory purposes.

## 3. Antimicrobial activity of compounds 3ha, 3ka, 3fa, 3hc, and 3eb

### 3.1. Materials and methods

The microbial culture experiment required potato dextrose agar (PDA), nutrient agar (NA), cation control Mueller Hinton broth and RPMI 1640 medium (without sodium bicarbonate supplemented with 0.165 moles per litre of morpholine propane sulfonic acid (MOPS)) from Hi-Media Laboratories Limited (Mumbai, Maharashtra, India). The RPMI 1640 formulation was obtained from the Roswell Park Memorial Institute (Buffalo, NY, USA). Drugs (amphotericin B, meropenem, and vancomycin) and solvents such as phosphate buffered saline (PBS) and sodium chloride (NaCl) were acquired from Sigma-Aldrich (St. Louis, MO, USA). Falcon tubes, Eppendorf tubes, and other plastic ware were sourced from Tarson Product Private Limited (Kolkata, West Bengal, India). Moreover, 96-well flat-bottom and U-bottom microtiter plates were procured from Thermo Scientific (US). All reagents employed in this study were of analytical grade.

### 3.2. Microbial strains and growth conditions

We used the antimicrobial susceptibility test to investigate the impact of synthesised compounds (3ha, 3ka, 3fa, 3hc, and 3eb) on Gram-positive bacteria (*Enterococcus faecalis ATCC 29212* and *Staphylococcus auris ATCC 25923*), Gram-negative bacteria (*Escherichia coli ATCC 25922* and *Pseudomonas aeruginosa ATCC 27853*) and against fungal cells (*Candida albicans ATCC 90028* and *Candida tropicalis ATCC 750*). Lyophilised cultures of bacteria and fungi were obtained from the American Type Culture Collection (ATCC) and stored at −80 °C. The bacterial strains were subcultured in nutrient agar (NA). The fungal strains were subcultured in Sabouraud dextrose agar (SDA) medium and incubated at 37 °C for 24 hours. Subsequently, the growth rate and microscopic characteristics were observed using Gram stain for all strains. These strains were then preserved at −20 °C for further experiments.

### 3.3. Inoculum preparation

The freeze-frozen preserved culture strains of the above-mentioned bacteria and fungi were subcultured on NA and SDA plates and incubated at 37 °C for 24 hours. Bacterial inoculum was prepared according to the CLSI (Clinical Laboratory Standard Institute) guidelines by adding 1–2 colonies in a normal saline tube, then vortexing, and matching with MacFarland suspension of 0.5 OD at 565 nm wavelength. Subsequently, inoculum suspensions must have a concentration of 10^7^ cells per mL for bacteria, 10^6^ cells for yeast cells, and 10^5^ cells for filamentous fungi.

## 4. Antifungal and antibacterial activity test by well diffusion method

The agar well diffusion method was used to assess *in vitro* antibacterial and antifungal activities by synthesised compounds (3ha, 3ka, 3fa, 3hc, and 3eb). Gram-positive bacteria (*Enterococcus faecalis ATCC 29212* and *Staphylococcus auris ATCC 25923*) and Gram-negative bacteria (*Escherichia coli ATCC 25922* and *Pseudomonas aeruginosa ATCC 27853*) were sub-cultured on nutrient agar. Fungal Strains (*Candida albicans ATCC 90028* and *Candida tropicalis ATCC 750*) were subcultured on Sabouraud dextrose agar and incubated for 24 hours at 37 °C. The inoculum was prepared according to the previously described method. Subsequently, the fungal suspension was evenly spread over MHA plates, and the bacterial suspension was evenly spread over plain MHA plates. Wells were created in each inoculated culture plate using a sterile cork-borer, and 50 μL of test substances was added into each well. Specifically, 10 μg mL^−1^ of meropenem served as a positive control for Gram-negative bacteria, 2 μg mL^−1^ of vancomycin was taken as a positive control for Gram-positive bacteria and 32 μg mL^−1^ of amphotericin B as a positive control for fungal strains. Distilled water was taken as a negative control. Additionally, 50 μL of a 32 μg mL^−1^ solution of each produced compound (3ha, 3ka, 3fa, 3hc, and 3eb) was transferred into separate wells. The culture plates were subsequently incubated for 24 hours at a temperature of 37 °C. The above-described method was again applied for fungal strains. Following incubation, the zones were measured and recorded. All experiments were conducted in triplicate to ensure the reliability of results.

### 4.1. Minimal inhibitory concentration determination of synthesised compounds

The minimal inhibitory concentrations of synthesised compounds against Gram-positive bacterial strains (*Enterococcus faecalis ATCC 29212* and *Staphylococcus auris ATCC 25923*), Gram-negative bacterial strains (*Escherichia coli ATCC 25922* and *Pseudomonas aeruginosa ATCC 27853*) and fungal strains (*Candida albicans ATCC 90028* and *Candida tropicalis ATCC 750*) was determined utilising the broth micro-dilution method in 96-well U-shaped bottom microtiter plates and 96-well flat-bottom microtiter plates as per CLSI guidelines. A 1 mg mL^−1^ stock solution of each synthesised compound was prepared. The inoculum was prepared using a previously described method, and the resulting suspension was diluted to achieve a concentration of 10^7^ cells per mL for bacterial cells and 10^6^ for fungal cells. We used a 96-well microtiter plate for MIC determination. Initially, we conducted a two-fold serial dilution of synthesized compounds in RPMI 1640 with morpholine propane sulfonic acid (MOPS) buffer. Subsequently, we added 100 μL of the inoculum suspension to each well. We used 100 μL of meropenem as a positive control for Gram-negative bacteria, 100 μL of vancomycin as a positive control for Gram-positive bacteria and 100 μL of amphotericin B as a positive control for fungal strains. The microtiter plates were incubated for 24 hours at 37 °C, and MIC values were determined the following day by visually assessing the turbidity.

## 5. Results

### 5.1. Antimicrobial activity by well diffusion method

In our investigation, we assessed the antimicrobial efficacy of synthesized compounds using the well diffusion method against Gram-positive bacteria (*Enterococcus faecalis ATCC 29212* and *Staphylococcus auris ATCC 25923*), Gram-negative bacteria (*Escherichia coli ATCC 25922* and *Pseudomonas aeruginosa ATCC 27853*) and fungal strains (*Candida albicans ATCC 90028* and *Candida tropicalis ATCC 750*). The outcomes of this diffusion assay are illustrated in Table 3. Notably, 3ha, 3ka, 3fa, 3hc, and 3eb did not manifest any significant antimicrobial activity against the tested strain. In contrast, amphotericin B employed as a positive control showed a 15 mm zone of inhibition against fungal strain *Candida albicans* and an 18 mm zone of inhibition against *C. tropicalis*; meropenem showed a 30 mm zone of inhibition in *E. coli* and a 40 mm zone of inhibition in *Pseudomonas*, and vancomycin showed a 25 mm zone of inhibition in *Staphylococcus auris* and a 24 mm zone of inhibition in *Enterococcus faecalis*, as shown in Tables 3 and 4.

**Table 3:** Well diffusion against Gram-positive and Gram-negative bacteria^a^

Bacteria (+, −)	L1	L2	L3	L4	L5	Positive control
*S. auris*	0	0	0	0	0	25 mm
*E. faecalis*	0	0	0	0	0	24 mm
*P. aeruginosa*	0	0	0	0	0	40 mm
*E. coli*	0	0	0	0	0	30 mm

a Determination of the antimicrobial efficacy of synthesised compounds against Gram-positive bacteria (*Enterococcus faecalis ATCC 29212* and *Staphylococcus auris ATCC 25923*) and Gram-negative bacteria (*Escherichia coli ATCC 25922* and *Pseudomonas aeruginosa ATCC 27853*) using the well diffusion method.

**Table 4:** Well diffusion against *Candida* sp (fungal strain)^a^

Bacteria (+, −)	3ha	3ka	3fa	3hc	3eb	Positive control
*C. albicans*	0	0	0	0	0	15 mm
*C. tropicalis*	0	0	0	0	0	18 mm

a Determination of the antimicrobial efficacy of synthesised compounds against fungal strains (*Candida albicans ATCC 90028*, *Candida tropicalis ATCC 750*) using the well diffusion method.

### 5.2. Determination of MIC of synthesized compounds (3ha, 3ka, 3fa, 3hc, and 3eb)

**Fig. 3:**
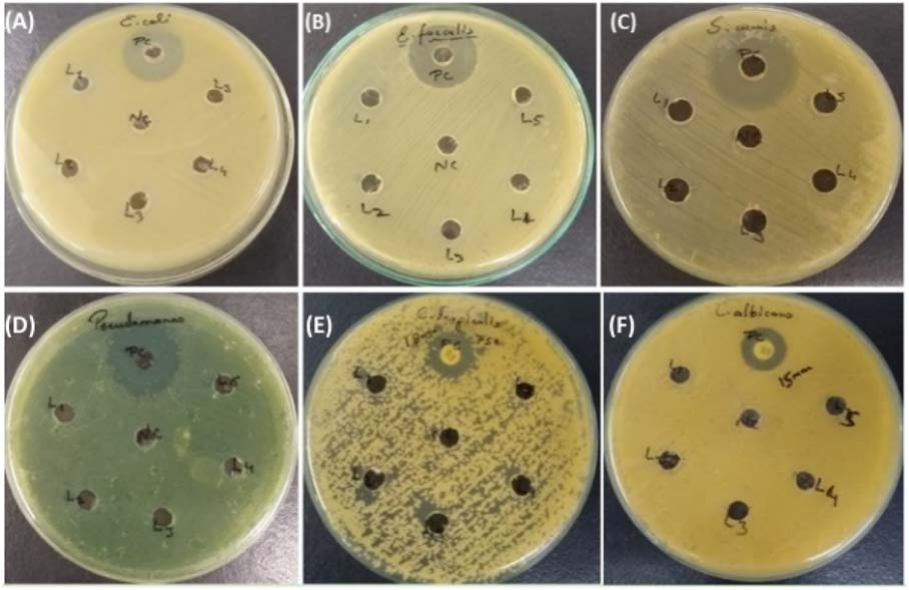
Results of the antimicrobial susceptibility test by agar well diffusion method against Gram-positive, Gram-negative and fungal strains. (A) *Enterococcus faecalis ATCC 29212*: positive control, (3ha), (3ka), (3fa), (3hc) and (3eb). (B) *Staphylococcus auris ATCC 25923*: positive control, (3ha), (3ka), (3fa), (3hc) and (3eb). (C) *Escherichia coli ATCC 25922*: positive control, (3ha), (3ka), (3fa), (3hc) and (3eb). (D) *Pseudomonas aeruginosa ATCC 27853*: positive control, (3ha), (3ka), (3fa), (3hc) and (3eb). (E) *Candida albicans ATCC 90028*: positive control, (3ha), (3ka), (3fa), (3hc) and (3eb). (F) *Candida tropicalis ATCC 750*: positive control, L1, L2, L3, L4, and L5 [(3ha), (3ka), (3fa), (3hc) and (3eb)].

The MIC of the synthesised compound against Gram-positive bacteria (*Enterococcus faecalis ATCC 29212* and *Staphylococcus auris ATCC 25923*), Gram-negative bacteria (*Escherichia coli ATCC 25922* and *Pseudomonas aeruginosa ATCC 27853*), and fungal strains (*Candida albicans ATCC 90028* and *Candida tropicalis ATCC 750*) was determined utilising the broth micro-dilution method in 96-well microtiter plates. The MIC results are presented in Table 5. Specifically, in bacterial strains, we observed MIC values of 2 μg mL^−1^ for 3ha and 3ka, and ≥16 μg mL^−1^ for 3fa, while *E. faecalis* was resistant to 3hc, and 3eb showed a MIC of 2 μg mL^−1^. In fungal strains, 3ha and 3ka gave MIC values of 1 and 2 μg mL^−1^, 3fa showed an MIC of 16 μg mL^−1^, *C. albicans* species was resistant to 3hc but 3hc gave an MIC of 2 μg mL^−1^ in *C. tropicalis* and finally L5 gave MICs of 2 μg Ml^−1^ in *C. albicans* and 1 μg mL^−1^ in *C. tropicalis*, as shown in Tables 5 and 6 (Fig. 3).

**Table 5:** MIC value against Gram-positive and Gram-negative bacteria^a^

Bacteria (+, −)	3ha	3ka	3fa	3hc	3eb
*S. auris*	2 μg mL^−1^	2 μg mL^−1^	16 μg mL^−1^	16 μg mL^−1^	2 μg mL^−1^
*E. faecalis*	2 μg mL^−1^	2 μg mL^−1^	>16 μg mL^−1^	R	4 μg mL^−1^
*P. aeruginosa*	2 μg mL^−1^	2 μg mL^−1^	16 μg mL^−1^	16 μg mL	2 μg mL^−1^
*E. coli*	1 μg mL^−1^	2 μg mL^−1^	16 μg mL^−1^	2 μg mL^−1^	2 μg mL^−1^

a MIC (mg mL^−1^) of imidazopyridine compounds (3ha, 3ka, 3fa, 3hc, and 3eb).

**Table 6:** MIC against *Candida* sp^a^

Fungi	3ha	3ka	3fa	3hc	3eb	Positive control
*C. albicans*	1 μg mL^−1^	2 μg mL^−1^	16 μg mL^−1^	R	2 μg mL^−1^	0.25 μg mL^−1^
*C. tropicalis*	2 μg mL^−1^	2 μg mL^−1^	16 μg mL^−1^	2 μg mL^−1^	1 μg mL^−1^	<0.015 μg mL^−1^

a Minimum inhibition concentration (mg mL^−1^) of imidazopyridine compounds (3ha, 3ka, 3fa, 3hc, and 3eb).

### 5.3. Discussion

Antimicrobial susceptibility testing (AST) is a crucial laboratory technique conducted by clinical laboratory scientists to determine the most effective antimicrobial treatment for individual patients. Additionally, it plays a vital role in assessing the quality of treatment offered by healthcare facilities and national programs to manage and prevent infectious diseases.^49^ In our study, the absence of any inhibition zones in antimicrobial susceptibility testing indicates that the synthesised compound cannot likely hinder microorganism growth under controlled laboratory conditions. Various factors, including the properties of the compounds, such as polarity and volatility,^50,51^ its interactions with microorganisms, its resistance mechanism and the experimental procedure can contribute to this outcome. MIC is another quantitative antimicrobial susceptibility method.^52^ We observed that the MIC for bacterial strains was 2 μg mL^−1^ for compounds 3ha and 3ka, while 3fa exhibited a MIC value of ≥16 μg mL^−1^, and *E. faecalis* demonstrated resistance to 3hc. 3eb showed a MIC value of 2 μg mL^−1^. For fungal strains, 3ha had a MIC value of 1 μg mL^−1^, 3ka had a MIC value of 2 μg mL^−1^, and 3fa had a MIC value of 16 μg mL^−1^, *C. albicans* was resistant to 3hc, while 3hc showed a MIC value of 2 μg mL^−1^ in *C. tropicalis*. Finally, 3eb displayed a MIC value of 2 μg mL^−1^ in *C. albicans* and 1 μg mL^−1^ in *C. tropicalis*. This provides a better understanding of the antimicrobial activity of the compound. Even if a compound does not produce a clear or visible zone in the well diffusion test, it still possesses some level of antimicrobial effectiveness, as indicated by the MIC value. It is essential to systematically investigate and discuss these factors to comprehend the limitation of the compound in antimicrobial treatment—this information is valuable for guiding future research efforts.

## 6. Experimental section

### 6.1. General procedure for the synthesis of products

A mixture of 1.0 mmol of 2-bromoacetophenone, 1.0 mmol of 2-aminopyridine, 3 mL of C_2_H_5_OH and 30 mg PPL was introduced to an round-bottom flask (50 mL), then the mixture was subjected to shaking at 160 rpm with end-over-end rotation at 30 °C for a specific time. The reaction was monitored by TLC (petroleum ether–ethyl acetate ¼ 5 : 1, v/v), and 5 mL of ethyl acetate was added into the reaction mixture to dissolve any solids if necessary. Then, the mixture was filtered through a paper filter to remove the enzymes, and the solvent was evaporated. The solid residue was recrystallised with ethanol, yielding the target compounds. ^1^H NMR spectra were recorded on a Bruker Avance 500 spectrometer at 500 MHz in DMSO, using TMS as the internal standard.

### 6.2. Procedure for gram-scale synthesis

A mixture of acetophenone (2.4 g, 20.0 mmol), 2-amino pyridine (2.16 g, 24.0 mmol), and PPL (30 mg) and C_2_H_5_OH (30 mL) was added to a 50 mL round-bottom flask and stirred continuously. The progress of the reaction was monitored by TLC (petroleum ether–ethyl acetate ¼ 5 : 1, v/v), and 5 mL of ethyl acetate was added into the reaction mixture to dissolve any solids, if necessary. Then, the mixture was filtered through a paper filter to remove enzymes, and the solvent was evaporated. The solid residue was recrystallised with ethanol, yielding target compounds. ^1^H NMR spectra were recorded on a Bruker Avance 500 spectrometer at 500 MHz in DMSO, using TMS as the internal standard.

### 6.3. Procedure for control experiment with TEMPO

Acetophenone (1a) (1.0 mmol), 2-amino pyridine (2a) (1.0 mmol), PPL (0.5 equiv.), TBHP (70% aq., 1 equiv.) and TEMPO (1.0 mmol) were placed in a 50 mL round-bottom flask and stirred for 2 : 30 hours. The progress of the reaction was monitored by TLC. The reaction was monitored by TLC (petroleum ether–ethyl acetate ¼ 5 : 1, v/v), and 5 mL of ethyl acetate was added into the reaction mixture to dissolve any solids, if necessary. Then, the mixture was filtered through a paper filter to remove enzymes, and the solvent was evaporated. The solid residue was recrystallised with ethanol, yielding target compounds. ^1^H NMR spectra were recorded on a Bruker Avance 500 spectrometer at 500 MHz in DMSO, using TMS as an internal standard.

## 7. Conclusions

In this work, a completely regioselective, environmentally friendly technique for the straightforward synthesis of physiologically active imidazole-fused nitrogen-bridgehead heterocycles has been developed by the use of 2-halocarbonyl compounds with 2-aminopyridines as starting materials. This strategy involves an efficient biocatalytic route, using lipase as a biocatalyst and ethanol as an eco-friendly reaction medium. This reaction avoids toxic catalysts and volatile organic solvents. The merits of this protocol are high atom economy, operational simplicity, mild reaction conditions, easy workup and purification process, and good yields of desired products in short reaction times. After studying the antimicrobial and antifungal activities of desired products to understand their limitations in antimicrobial treatment, it is important to conduct a thorough investigation and discourse on these issues. This data is important for directing future investigations.

## Author contributions

Manjit Singh and Aishwarya Nikhil did the experimental work and wrote the main text. Vijay B. Yadav and Munish Gupta helped prepare the manuscript, and Manisha Malviya helped with conceptualisation, supervision, reviewing and editing the original draft. All authors reviewed the manuscript.

## Conflicts of interest

The authors declare no conflict of interest.

## Supplementary Material

RA-014-D3RA07145F-s001
