# Supplementary material for: Synthesis of imidazole-fused nitrogen-bridgehead heterocycles catalysed by lipase and their antifungal and antimicrobial bioactivity
Source: RSC Adv. 2024 Feb 7;14(8):5037–44. doi: 10.1039/d3ra07145f (PMC10848895; doi:10.1039/d3ra07145f)

Electronic Supplementary Material (ESI) for RSC Advances.
This journal is © The Royal Society of Chemistry 2024

Supporting Information

Synthesis of Imidazole-fused nitrogen-bridgehead heterocycles catalysed by lipase and their antifungal and anti-microbial bioactivity.

Manjit Singh[a], Manisha Malviya *Vijay B. Yadav[b], Aishwarya Nikhil[c] &Munesh Gupta[c,]

[a] Department of Chemistry IIT (BHU) Varanasi

[b]Department of Chemistry University of Allahabad

[c]Department of Microbiology, IMS(BHU) Varanasi

Email: manisha.apc@itbhu.ac.in

LIST OF CONTESTS: page no.

1. General Information 2

2. Experimental procedure for the synthesis of 2-phenylimidazo[1,2-a] pyridine 2

3. Analytical data of products 3

4. References 13

5. ^{1}H,^{13}C and Mass spectra 14

1.) General Information

All chemicals and solvents were purchased from Sigma Aldrich and used without purification. Melting points were measured on the Stewart melting point apparatus in one side open capillary and are uncorrected. The progress of the reaction was monitored by thin-layer chromatography on a glass plate coated with silica gel G-234 and fluorescent silica gel. A UV lamp and iodine chamber were used to visualize the reaction spot. High-Resolution Mass Spectrometry (HRMS) was performed using a SCIEX X500R QTOF (TOF-MS) system. ^{1}H and ^{13}C NMR spectra were recorded on Bruker Avance 500 MHz spectrometer in DMSO d6 using TMS as internal standard 500 MHz (^{1}H) and 126 MHz (^{13}C). All chemical shifts were

reported in ppm with reference to the DMSO peak (2.50 for ^1H and 39.50 for ^{13}C NMR). All coupling constants are reported in hertz (Hz). Abbreviations are s: singlet, d: doublet, t: triplet, q: quartet, bs: broad singlet, dd: double doublet. All synthesized products were confirmed by using melting point, ^1H and ^{13}C NMR, and comparison with the literature reports.

2.) General procedure for the synthesis of 2-phenylimidazo[1,2-a] pyridine

2.1 Experimental procedures for the synthesis of 2-phenylimidazo[1,2-a] pyridine

The mixture of 1.0 mmol 2-bromoacetophenone, 1.0 mmol 2-aminopyridine, 3 ml C_2H_5OH and 30 mg PPL was introduced to an RB (50 ml), then the mixture was subjected to shaking at 160 rpm with end-over-end rotation at 30 °C for a certain time. The reaction was monitored by TLC (petroleum ether–ethyl acetate ¼ 5: 1, v/v), and 5 ml ethyl acetate was added into the reaction mixture to dissolve any solids if necessary. Then, the mixture was filtered through a paper filter to remove the enzymes, and the solvent was evaporated. The residue was recrystallised with ethanol, yielding the target compounds. ^1H NMR spectra were recorded on a Bruker Avance 500 spectrometer at 500 MHz in DMSO, using TMS as an internal standard. IR spectra were recorded on a Bruker Equinox-55 spectrophotometer, using KBr discs in the 4000– 400 cm^{-1} regions. All of the enzymes were purchased from Aldrich and used directly. The enzymatic units of all of the enzymes are described as follows: trypsin from porcine pancreas (2500 units per mg), lipase from porcine pancreas (≥200 units per mg), diastase (≥3.5 units per mg), a-amylase from Aspergillus oryzae (≤30 units per mg), a-amylase from hog pancreas (10 units per mg), Amano lipase M from Mucor javanicus (10 units per mg). All reagents were analytical reagent grade and used directly without further purification.

2.2. Procedure for gram-scale synthesis:

A mixture of 2- bromo acetophenone (2.4 g, 20.0 mmol), 2-amino pyridine ((2.16 g, 24.0 mmol), PPL-Lipase (30 mg.) and C_2H_5OH (50 ml.) were added in 30, ml RB and steerer continuously, and the progress of the reaction was monitored by TLC. The reaction was monitored by TLC (petroleum ether–ethyl acetate ¼ 5: 1, v/v), and 5 ml ethyl acetate was added into the reaction mixture to dissolve any solids if necessary. Then, the mixture was filtered through a paper filter to remove the enzymes, and the solvent was evaporated. The solid residue was recrystallised with ethanol, yielding the target compounds.^1H NMR spectra

were recorded on a Bruker Avance 500 spectrometer at 500 MHz in DMSO, using TMS as an internal standard.

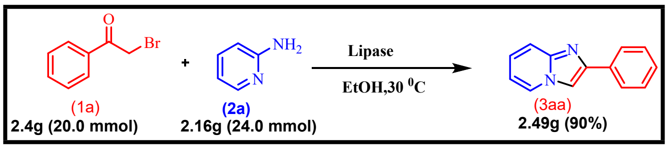

3. A) Analytical data of products:

[3.1] ^{1}H and ^{13}C NMR of the 2-phenylimidazo[1,2-a] pyridine(3aa)[1]

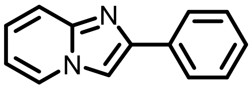	Purified by recrystallisation using aqueous ethanol; white solid; yield: 960 mg (95%); mp 130- 132°C. **^{1}H NMR** (500 MHz, DMSO): δ 8.05 (d, J =7.0 Hz, 1H, arom H), 7.95 (dd, J =8.5 Hz, J =1.5 Hz, 2H, arom H), 7.71 (s, 1H, arom H), 7.59 (d, J =9.0 Hz, 1H, arom H), 7.42 (t, J =7.4 Hz, 2H, arom H), 7.28 (t, J =7.5 Hz, 1H, arom H), 7.14-7.10 (m, 1H, arom H), 6.57 (td, J =7.0 Hz, J =1.0 Hz, 1H, arom H). **^{13}C NMR** (126 MHz, $CDCl_3$): 144.91, 143.83, 134.89, 127.90, 129.15, 125.21, 126.77, 125.85, 116.68, 111.60, 107.30.IR (KBr, v, cm^{-1}): 3433, 3140, 3042, 1945, 1658, 1636, , 1223, 1067, 847, 765 **HRMS** (ESI): Anal. Calcd. For $C_{13}H_{11}N_2$ [M+H]+ 195.0922; Found: 195.0882

[3.2] ^{1}H and ^{13}C NMR of the 2-(p-Tolyl) imidazo [1,2-a] pyridine (3ba):[1]

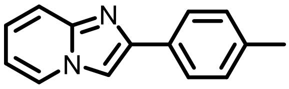	White solid; yield 193 mg (93%); m.p. 143-144 °C; **^{1}H NMR** (500 MHz, DMSO) δ (ppm): 8.93 (d, 1H), 7.84 (d, 2H), 7.77 (s, 1H),7.43 (d, 1H), 7.37 (dd, 1H), 7.26 (d, 2H), 2.44 (s, 3H); **^{13}C NMR** (126 MHz, $CDCl_3$) δ (ppm): 167.38, 143.2, 139.3, 133.5, 131.5, 129.4, 128.6, 126.1, 119.5, 106.5, 14.33; **HRMS** (ESI): calculated for $C_{14}H_{12}N_2$ [M $^+$] H^+: 209.1073, found 209.1077.

[3.3] ^{1}H and ^{13}C NMR of the 2-(4-methoxyphenol) imidazo[1,2-a]pyridine (3ca)[2]

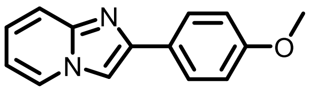	White solid; yield 210 mg (90%); m.p. 138-139 °C; **^{1}H NMR** (500 MHz, DMSO) δ (ppm): 8.63 (dd, 1H), 8.05 (d, 2H), 7.98 (s, 1H), 7.62 (d, 1H), 7.17 – 7.14 (m, 1H), 6.96 (d, 2H), 6.74 (t, 1H), 3.84 (s, 3H); **^{13}CNMR** (126 MHz, $CDCl_3$) δ (ppm):161.32,144.84, 128.74, 126.13, 125.5, 124.5, 123.6, 117.4, 114.2, 110.4, 107.3, 56.4.**HRMS** (ESI): calculated for $C_{14}H_{12}N_2O$ [M $^+$H]$^+$: 225.1022, found 225.1025.

[3.4] ^{1}H and ^{13}C NMR of the 2-(4-nitrophenyl) imidazo[1,2-a]pyridine (3da)[1]

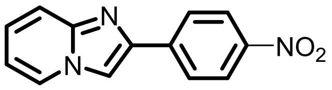	Yellow solid; yield 227 mg (70%); m.p. 202-203 °C; **^{1}H NMR** (500 MHz, DMSO) δ (ppm): 8.54 – 8.44 (m, 1H), 7.93 (d, 1H), 7.92 (dd, 2H), 7.64 (s, 1H), 7.63 (d, 1H), 7.25 (t, 1H), 7.21 (d, 1H), 6.91 (t, 1H)**;** **^{13}C NMR** (126 MHz, $CDCl_3$) δ (ppm): 145.37, 143.67, 133.70, 132.12, 128.03, 127.44, 125.68, 121.15, 117.16, 112.91,109.98. **HRMS** m/z (ESI):calcd. for [$C_{13}H_9N_3O_2^+$] H^+ :240.0768 Found: 240.0769

[3.5] ^{1}H and ^{13}C NMR of the 4-(Imidazo[1,2-a]pyridin-2-yl)phenol (3ea)[4]:

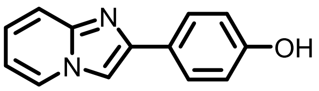	Brown solid; yield 185 mg (75%); m.p. 230-231 °C; **^{1}H NMR** (500 MHz, DMSO) δ (ppm): 8.80 (s, 1H), 8.45 (d, 1H), 8.02 (s, 1H), 7.98 (d, 2H), 7.96 (d, 1H), 7.84 – 7.79 (m, 1H), 7.26 (d, 2H), 6.06 (s, 1H); **^{13}C NMR** (126 MHz, $CDCl_3$) δ (ppm): 156.18, 143.95, 128.01, 127.37, 126.6, 125.49, 117.09, 116.13, 115.96, 112.78, 109.41, 107.46 **HRMS (ESI):** calculated for $C_{13}H_{10}N_2O$

	[M $^{+}$] H^{+}: 211.0866, found 211.0870.

[3.6] ^{1}H and ^{13}C NMR of the 2-(4-Bromophenyl) imidazo[1,2-a] pyridine (3fa)[5]:

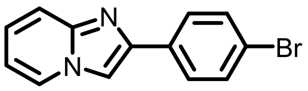	Yellow solid; yield 211 mg (90%); m.p. 206-207 °C; **^{1}H NMR** (500 MHz, DMSO) δ (ppm): 8.53 (d, 1H), 8.44 (d, 2H), 8.00 (s, 1H), 7.98 (d, 1H), 7.41 (d, 2H), 7.59-726 (dd, 1H), 6.91 (t, 1H); **^{13}C NMR** (126 MHz, $CDCl_3$) δ (ppm): 145.78, 143.8, 133.24, 132.18, 129.05, 126.24, 125.59, 117.24, 116.7, 113.7, 109.96; **HRMS (ESI):** calculated for $C_{13}H_9BrN_2$ [M^{+}] H^{+}: 273.0027 (for 79Br) and 275.0007 (for 81Br), found 273.0029 (for 79Br) and 275.0009 (for 81Br).

[3.7] ^{1}H and ^{13}C NMR of the 2-(4-Fluorophenyl) imidazo[1,2-a] pyridine (3ga)[1]:

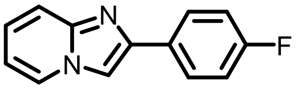	Pale yellow solid; yield 199 mg (65%); m.p. 164-165 °C; **^{1}H NMR** (500 MHz, DMSO) δ (ppm): 8.52 (d, 1H), 7.99 – 7.57 (m, 2H), 7.28 (s, 1H), 7.26 (d, 1H), 7.24 (t, 1H), 7.13 – 7.08 (m, 2H), 6.93 (t, 1H); **^{13}C NMR** (126 MHz, $CDCl_3$) δ (ppm): 163.06, 147.18, 145.25(J=97Hz.),129.84(J=3.2Hz),128.96,127.09(J=8.06Hz),125.68, 125.47, 116.74 116.132,(J=21.5Hz),111.78, 107.81.**^{19}F NMR** (471 MHz, DMSO)δ-108.75.**HRMS** (ESI): calculated for $C_{13}H_9FN_2$ [M $^{+}$]H^{+}: 213.0823, found 213.0815.

[3.8] ^{1}H and ^{13}C NMR of the 4-(imidazo[1,2-a]pyridine-2-yl)benzonitrile(3ha)[7]:

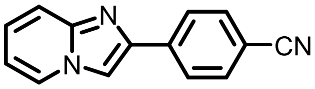	Colourless solid; yield 187 mg (65%); m.p. 200-201 °C; ^{1}**H NMR** (500 MHz, DMSO) δ (ppm): 12.73 (s, 1H), 8.52 (d, 1H), 8.44 (s, 1H), 7.94 (d, 2H), 7.92-791 (t, 2H), 7.64-7.63 (dd, 1H), 7.26 – 6.911(m, 2H); ^{13}**C NMR** (126 MHz, $CDCl_3$) δ (ppm):145.37, 143.67, 133.70, 132.02, 128.03, 127.53, 127.44, 125.68, 121.15, 117.16, 112.91, 109.98. **HRMS (ESI):** calculated for $C_{14}H_9N_3$ [M $^{+}$] H^{+}: 220.0869, found 220.0869.

[3.9] ^{1}H and ^{13}C NMR of the 2-(4-chlorophenyl) imidazo[1,2-a] pyridine (3ia)[1]:

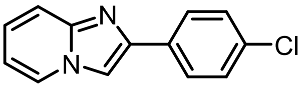	Yellow solid; yield 227 mg (85%); m.p. 202-203 °C; ^{1}**H NMR** (500 MHz, DMSO) δ (ppm): 8.53 – 8.44 (m, 1H), 8.00 (d, 1H), 7.98 (dd, 2H), 7.53 (s, 1H), 7.49 (d, 1H), 7.27 (t, 1H), 7.22 (d, 1H), 6.91 (t, 1H); ^{13}**C NMR** (126 MHz, $CDCl_3$) δ (ppm): 145.37, 145.0, 143.64, 133.55, 129.21, 127.71, 127.43, 125.5, 123.66, 117.16, 112.96, 114.1, 109.96 **HRMS (ESI)**: calculated for $C_{13}H_9ClN_2$ [M $^{+}$] H^{+}: 229.0527, found 229.0531.

[3.10] ^{1}H and ^{13}C NMR of the 2-(Pyridin-2-yl)imidazo[1,2-a]pyridine (3ja)[2]:

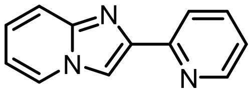	Brown solid; yield 166 mg (80%); m.p. 240-241 °C; ^{1}**H NMR** (500 MHz, DMSO) δ (ppm): 8.64 – 8.63 (m, 2H), 8.24 (s, 1H), 8.23 (dd, 1H), 7.86 (d, 1H), 7.84 (d, 1H), 7.71 – 7.57 (m, 2H), 6.93 (d, 1H); ^{13}**C NMR** (126MHz, DMSO) δ (ppm): 153.38, 151.02, 147.86, 141.19, 139.60, 127.09, 125.68, 123.22, 116.74, 116.71, 111.00, 100.86. **HRMS** (ESI): Anal. Calcd. For $C_{12}H_9N_3$ [M^{+}] H^{+} 195.08; Found: 196.08

[3.11] ^{1}H and ^{13}C NMR of the 2-(Thiophen-2-yl)imidazo[1,2-a]pyridine (3ka)[7]:

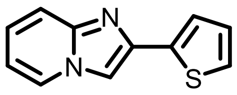	Yellowish white solid; yield 184 mg (60%); m.p. 135-136 °C; **^{1}H NMR** (500 MHz, DMSO) δ (ppm): 8.51 (d, 1H), 8.76 (s, 1H), 8.28 (d, 1H), 7.25 – 7.14 (m, 1H), 7.61 – 7.56 (m, 1H), 7.54 – 7.51 (m, 1H), 7.25 – 7.14 (m, 1H), 6.90 (t, 1H); **^{13}C NMR** (126 MHz, $CDCl_3$) δ (ppm): 145.04, 140.23, 138.26, 128.45, 127.26, 125.92, 125.59, 124.06, 116.79, 112.88, 108.67. **HRMS (ESI)**: calculated for $C_{11}H_8N_2O$ [M^+] H^+: 185.0709, found 185.0695.

[3.12] ^{1}H and ^{13}C NMR of the2-(Naphthalen-1-yl) imidazo[1,2-a]pyridine (3la):[2,5]

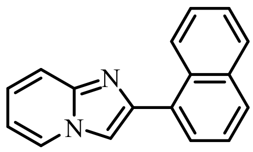	Yellowish liquid; yield 227 mg (82%); **^{1}H NMR** (500 MHz,) δ (ppm): 8.62 – 8.55 (m, 1H), 8.16 (d, 1H), 7.91 – 7.86 (m, 2H), 7.82 (d, 2H), 7.70 (d, 1H), 7.57 – 7.48 (m, 3H), 7.22 – 7.17 (m, 1H), 6.80 (s, 1H); **^{13}C NMR** (126 MHz, DMSO) δ (ppm): 146.48, 144.34, 138.01, 130.87, 132.63, 127.59, 129.47, 126.83, 125.55, 127.06, 124.89, 126.66, 124.52, 123.71, 116.85, 116.74, 111.00. **HRMS (ESI):** calculated for $C_{17}H_{12}N_2$ [M^+] H^+: 245.1073, found 245.1076.

<u>[3.13]</u>^{1}H and ^{13}C NMR of the <u>8-Methyl-2-phenylimidazo[1,2-a]pyridine (3ab)[1]:</u>

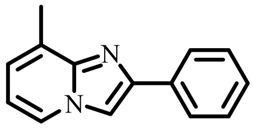	Pale yellow solid; yield 189 mg (94%); m.p. 119-120 °C; **^{1}H NMR** (500 MHz, $CDCl_3$) δ (ppm): 8.69 (t, 1H), 7.98 (d, 2H), 7.62 (s, 1H), 7.82 (dd, 2H), 7.33 – 7.14 (m, 1H), 6.84 (d, 1H), 3.85 (t, 1H), 2.65 (s, 3H); **^{13}C NMR** (126 MHz, DMSO) δ (ppm): 161.06, 143.38, 142.69, 131.69, 128.67, 126.66, 123.47, 122.89, 117.4, 55.93, 19.2.**HRMS (ESI)**: calculated for $C_{14}H_{12}N_2$ [M^+] H^+ : 209.1073, found 209.1074.

[3.14]^{1}Hand^{13}CNMRofthe 2-(4-Methoxyphenyl)-8-methylimidazo[1,2-a]pyridine (3cb)[2]:

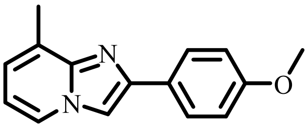	Pale yellow solid; yield 209 mg (88%); m.p. 132-133 °C; ^{1}**H NMR** (500 MHz, DMSO) δ (ppm): 7.94 (d, 1H), 7.87 (d, 2H), 7.72 (s, 1H), 6.96 (d, 2H), 6.91 (d, 1H), 6.65 (t, 1H), 3.83 (s, 3H), 2.64 (s, 3H); ^{13}**C NMR** (126 MHz, $CDCl_3$) δ (ppm): 160.5, 147.2, 144.2, 126.5, 126.4, 125.9, 122.4, 124.2, 115.1, 113.2, 106.8. 54.4, 16.2. **HRMS (ESI):** calculated for $C_{15}H_{14}N_2O$ [M^+] H^+: 239.1179, found 239.1173.

[3.15] ^{1}H and^{13}C NMR of 8-Methyl-2-(3-nitrophenyl)imidazo[1,2-a]pyridine (3db)[5]:

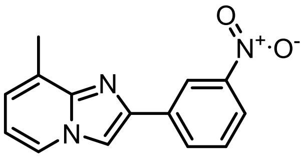	Yellow solid; yield 233 mg (75%); m.p. 168-169 °C**;** ^{1}**H NMR** (500 MHz, DMSO) δ (ppm):8.63 (m, 1H), 8.25 (dd, 1H), 8.14 – 8.10 (m, 1H), 7.97 (d, 1H), 7.84 (s, 1H), 7.68 (t, 1H), 7.05 (dd, 1H), 7.84 (t, 1H), 2.64 (s, 3H); ^{13}**C NMR** (126 MHz, $CDCl_3$) δ (ppm): 148.44, 141.24, 140.73, 140.1, 134.76, 132.79, 129.69, 128.10, 125.92, 124.49, 123.23, 123.18, 108.92, 108.46,19.11 **HRMS** (ESI): Anal. Calcd. For $C_{14}H_{11}N_3O_3$ [M^+] H^+ 253.0922; Found: 254.0882

[3.16]^{1}Hand^{13}C NMR of the2-(4-Chlorophenyl)-8-methylimidazo[1,2-a]pyridine (3eb)[8]:

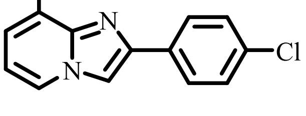	White solid; yield 226 mg (78%); m.p. 119-120 °C; ^{1}**H NMR** (500 MHz, DMSO) δ (ppm): 8.44 (d, 1H), 8.37 (d, 2H), 8.01 (s, 1H), 7.99 (d, 2H), 7.06 (d, 1H), 6.83 (t, 1H), 2.53 (s, 3H); ^{13}**C NMR** (126 MHz, $CDCl_3$) δ (ppm): 145.86, 143.01, 133.47, 132.43, 129.35, 127.70, 126.5, 125.6, 124.5, 123.6, 110.7, 17.09. **HRMS** (ESI): Anal. Calcd. For $C_{14}H_{11}ClN_2$ [M^+] H^+ 242.0922; Found: 242.0621

[3.17]]^{1}Hand^{13}C NMR of the **2-(4-Bromophenyl)-8-methylimidazo[1,2-a]pyridine (3fb)[9]:**

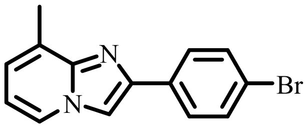	Yellow solid; yield 261 mg (91%); m.p. 131-132°C; **^{1}H NMR** (500 MHz, DMSO) δ (ppm): 8.43 (d, 1H), 7.86 – 7.81 (m, 2H), 7.78 (s, 1H), 7.54 (d, 2H), 6.95 (d, 1H), 6.72 – 6.63 (m, 1H), 2.64 (s, 3H); **^{13}C NMR** (126 MHz, $CDCl_3$) δ (ppm): 155.06,146.25,142.61, 137.13, 133.51, 132.40, 128.01, 121.00, 112. 64, 110.40, 17.04 **HRMS (ESI):** calculated for $C_{14}H_{11}BrN_2$ [M^+ H^+: 287.0178 (for 79Br) and 289.0163 (for 81Br), found 287.0166 (for 79Br) and 289.0170 (for 81Br).

[3.18]] ^{1}H& ^{13}C NMR of the2-(4-Fluorophenyl)-8-methylimidazo[1,2-a]pyridine (3gb)[1]:

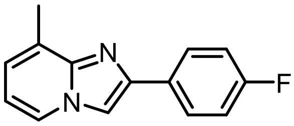	White solid; yield 199 mg (75%); m.p. 128-129 °C**; ^{1}H NMR** (500 MHz, DMSO) δ (ppm): 8.14 (s, 1H), 7.70 (dd, 2H), 7.58 (s, 1H), 7.16 – 7.04 (m, 2H), 6.97 – 6.93 (m, 1H), 6.84 (t, 1H), 2.59 (s, 3H); **^{13}C NMR** (126 MHz, CDC_{13}) δ (ppm): 163.06, 141.37, 141.24(d, J=16.3Hz.),129.84,128.96,(d, J=15.1Hz). 128.10, 125.92, 124.49, 116.32, 108.46, 108.45, (sJ=1.26Hz.) 19.11. **^{19}F NMR** (471 MHz, DMSO) δ -121.66. **HRMS (ESI):** calculated for $C_{14}H_{11F}N_2$ [M^+] H^+: 226.0178 found 227.0166.

[3.19]] ^{1}H and ^{13}C NMR of the 2-(8-Methylimidazo[1,2-a]pyridin-2-yl) phenol (3hb)[3]:

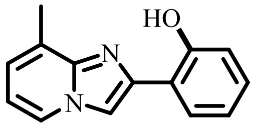	White solid; yield 195 mg (87%); **^{1}H NMR** (500 MHz, DMSO) δ (ppm): 12.94 (s, 1H), 8.47 (s, 1H), 8.46 (dd, 1H), 7.87 (d, 1H), 7.25 – 7.18 (m, 1H), 6.94 – 6.92 (m, 2H), 6.90 (t, 1H), 6.89 (d, 1H), 2.50 (s, 3H); **^{13}C NMR** (126 MHz, $CDCl_3$) δ (ppm): 156.67, 143.80, 143.08, 129.55, 126.88, 125.71, 125.09, 124.86, 118.52, 117.49, 117.24, 113.49, 110.01, 16.81.**HRMS** (ESI): Anal. Calcd. For $C_{14}H_{12}N_2O$ [M^+] H^+ 224.0917; Found: 225.0100

[3.20]] ^{1}H and ^{13}C NMR of the 4-(8-Methylimidazo[1,2-a]pyridin-2-yl)phenol (3ib)[7]:

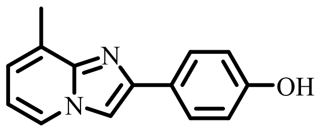	Brown solid; yield 199 mg (89%); **^{1}H NMR** (500 MHz, DMSO) δ (ppm): 10.51 (s, 1H), 8.39 (s, 1H), 8.08(dd, 2H), 7.96 (s, 1H), 7.41 (s, 1H), 7.81 – 7.02 (m, 3H), 6.91 – 6.91 (m, 1H), 6.83 (d, 1H), 6.76 – 6.71 (m, 1H), 2.60 (s, 3H); **^{13}C NMR** (126 MHz, DMSO) δ (ppm): 157.99, 141.77, 141.24, 128.43, 128.10, 125.92, 125.07, 124.49, 116.47, 108.46, 19.11. **HRMS** (ESI): Anal. Calcd. For $C_8H_8N_2O$ [M^+] H^+ 148.0617; Found: 148.0701.

[3.21] ^{1}H &^{13}C NMR of the 6-Chloro-2-phenylimidazo[1,2-a]pyridine (3ac):[5]

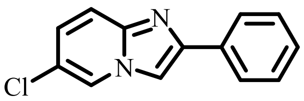	White solid; yield 213 mg (93%); m.p. 207-208 °C; **^{1}H NMR** (500 MHz, DMSO) δ (ppm): 8.28 (d, 1H), 7.83 (d, 2H), 7.66 (s, 1H), 7.62 (d, 1H), 7.45 (t, 2H), 7.39 (d, 1H), 7.32 (dd, 1H); **^{13}C NMR** (126 MHz, $CDCl_3$) δ (ppm): 147.17, 145.57, 132.27, 132.26, 129.78, 128.03, 127.06, 126.65, 115.91, 109.0, 107.53. **HRMS (ESI):** calculated for $C_{13}H_9ClN_2$ [M^+] H^+: 229.0527, found 229.0515.

[3.22] ^{1}H ^{13}CNMR of the 2-(4-Bromophenyl)-6-chloroimidazo[1,2-a]pyridine (3fc):[2]

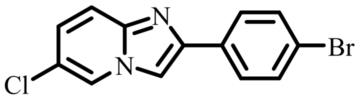	Yellow solid; yield 277 mg (90%); m.p. 199-200 °C; **^{1}H NMR** (500 MHz, DMSO) δ (ppm): 8.27 (dd, 1H), 7.82 – 7.65 (m, 3H), 7.60 (d, 3H), 7.51-739 (dd, 1H); **^{13}C NMR** (126 MHz, $CDCl_3$) δ (ppm): 147.17, 145.57, 132.44, 131.25, 129.78, 128.22, 129.03, 123.88, 115.91, 109.47, 107.53 **HRMS (ESI**): calculated for $C_{13}H_8BrClN_2$ [M^+] H^+: 306.9632 (for 79Br) and 308.9617 (for 81Br), found 306.9624 (for 79Br) and 308.9605 (for 81Br).

[3.23] [3.21] ^{1}H &^{13}C NMR of the 6-Chloro-2-(p-tolyl) imidazo[1,2-a]pyridine (3bc)[7]:

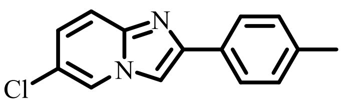	Pale yellow solid; yield 228 mg (90%); m.p. 125-126 °C; **^{1}H NMR** (500 MHz, DMSO) δ (ppm): 8.27 (d, 1H), 7.82 (d, 2H), 7.66 (s, 1H), 7.57 (d, 1H), 7.51 (d, 2H), 7.39 (d, 1H), 2.33 (s, 3H); **^{13}C NMR** (126 MHz, $CDCl_3$) δ (ppm): 147.17, 145.57, 137.20, 132.42, 129.78, 129.49, 128.08, 115.91, 109.47, 107.53, 107.12, 21.13.**HRMS (ESI):** calculated for $C_{13}H_9ClN_2$ [M^+] H^+: 229.0527, found 229.0515.

[3.24]^{1}Hand^{13}C NMR of the6-Chloro-2-(4-chlorophenyl) imidazo[1,2-a]pyridine (3ec)[1,6]:

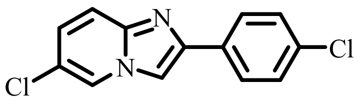	Pale yellow solid; yield 242 mg (80%); m.p. 206-207 °C; **^{1}H NMR** (500 MHz, DMSO) δ (ppm): 8.27 (d, 1H), 7.81 (d, 2H), 7.66 (s, 1H), 7.56 (d, 1H), 7.44 (d, 2H), 7.39 (dd, 1H); **^{13}C NMR** (126 MHz, $CDCl_3$) δ (ppm): 147.17, 145.57, 132.24, 131.39, 129.78, 129.36, 128.72, 128.03, 115.91, 109.47, 107.53 96 **HRMS (ESI)**: calculated for $C_{13}H_8Cl_2N_2$ [M^+] H^+: 262.0101, found 262.0531

[3.25] ^{1}H and ^{13}C NMR of the 2-(6-Chloroimidazo[1,2-a]pyridin-2-yl)phenol (3hc)[8]:

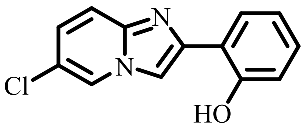	White solid; yield 218 mg (89%); m.p. 197-199 °C; **^{1}H NMR** (500 MHz, DMSO) δ (ppm): 8.26 (d, 1H), 7.77 (s, 1H), 7.64 – 7.56 (m, 2H), 7.42 (ddd, 2H), 7.38 (d, 1H), 6.98 (t, 1H), 6.91 (t, 1H), 1.27 (s, 1H); **13C NMR** (126 MHz, $CDCl_3$) δ (ppm): 160.10, 145.32, 140.95, 130.76, 129.78, 128.03, 127.15, 121.02, 120.31, 119.66, 115.91, 109.47, 105.41. **HRMS** (ESI): Anal. Calcd. For $C_{13}H_9ClN_2O$ [M^+] H^+ 244.0241; Found: 243.0321

[3.26]^{1}Hand ^{13}C NMR of the 8-(Phenoxymethyl)-2-phenylimidazo[1,2-a]pyridine (3ad)[9]:

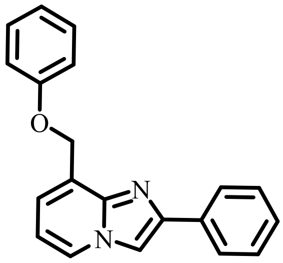	Pale yellow solid; yield 337 mg (89%); m.p. 130-131 °C**; ^{1}H NMR** (500 MHz, DMSO) δ (ppm): 8.16 – 7.84 (m, 2H), 7.63 (s, 1H), 7.42 (dd, 1H), 7.33 – 7.23 (m, 4H), 6.99 (dd, 2H), 6.91 (d, 1H), 6.89 (dd, 1H), 6.23 (d, 1H), 5.23 (s, 2H); **^{13}C NMR** (126 MHz, $CDCl_3$) δ (ppm): 159.22, 141.77, 140.85, 129.54, 129.09, 127.06, 126.52, 121.46, 120.45, 115.72, 108.45, 108.10, 112.5, 109.3, 103.4, 69.44 **HRMS** (ESI): Anal. Calcd. For $C_{13}H_9ClN_2O$ [M^+] H^+ 244.0241; Found: 243.0321.

[3.27] **] ^{1}H and ^{13}C NMR of** the2-methylimidazo[1,2-a] pyridine of (3ae)[2]

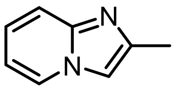	cherry-colored liquid; yield 237 mg (60%); m.p. 39-42 °C**; ^{1}H NMR** (500 MHz, $CDCl_3$) δ 8.20 (d, J = 6.7 Hz, 1H), 7.56 (d, J = 9.9 Hz, 1H), 7.49 (s, 1H), 7.17-7.06 (m, 1H), 6.90 (t, J = 6.7 Hz, 1H), 2.45 (s, 3H**) ^{13}C NMR** (126 MHz, $CDCl_3$) δ 143.46, 125.25, 124.04, 116.90, 111.85, 109.54, 14.51 **HRMS m/z** (ESI): calcd. for [$C_8H_8N_2$] [M^+] H^+ : 133.0767 Found : 133.0759

[3.28] ^{1}H and ^{13}C NMR of of the 2-pentylimidazo[1,2-a] pyridine of (3be)[9]

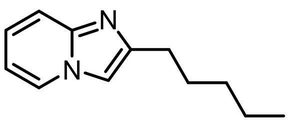	Pale yellow liquid; yield 137 mg (45%); m.p. 40-45 °C; **^{1}H NMR** (500 MHz, $CDCl_3$) δ (ppm): δ 8.20 (m, 1H), 7.57 (d, J = 9.0 Hz, 1H), 7.49 (s, 1H), 7.17 (m,1H), 6.90 (t, J = 6.8 Hz, 1H), 2.71 (t, J = 7.6 Hz, 2H), 1.79 (m, 2H), 1.32 (t, J = 7.4 Hz, 3H) **^{13}C NMR** (126 MHz, $CDCl_3$) δ 148.08, 144.91, 125.24, 123.91, 116.81, 111.71, 108.88, 29.70, 28.92, 22. **HRMS** m/z (ESI) **:**calcd. for [$C_{10}H_{12}N_2$] [M^+] H^+: 161.1072 Found: 161.1067

[3.29] ^{1}H and ^{13}C NMR of of the 2-propyllimidazo[1,2-a]pyridine of (3ce)[9]

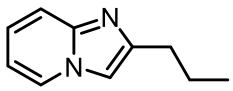	Pale yellow liquid; yield 127 mg (48%); m.p. 45-50 °C**; ^{1}H NMR** (500 MHz, $CDCl_3$) δ (ppm): 8.20 (m, 1H), 7.57 (d, J = 9.0 Hz, 1H), 7.49 (s, 1H), 7.17 (m,1H), 6.90 (t, J = 6.8 Hz, 1H), 2.70 (t, J = 7.6 Hz, 2H), 1.78 (m, 2H), 1.04 (t, J = 7.4 Hz, 3H) **^{13}C NMR** (126 MHz, $CDCl_3$) δ (ppm): δ 147.88, 144.96, 125.27, 123.96, 116.86, 111.76, 109.02, 30.99, 22.59, 14.05 **HRMS** (ESI): Anal. calcd. for [$C_{10}H_{12}N_2$ [M^+] H^+: 161.1073 Found: 161.1068

[3.30] ^{1}H and ^{13}C NMR of the 2-isopropylimidazo[1,2-a] pyridine **(3de)[2]**

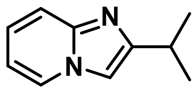	White solid; yield 157 mg (40%); m.p. 80-100 °C**; ^{1}H NMR** (500 MHz, $CDCl_3$) δ (ppm): δ 8.20 (dt, J = 6.8, 1.2 Hz, 1H), 7.57 (dd, J = 9.1, 0.7 Hz, 1H), 7.49 (s, 1H),7.17 (m, 1H), 6.90 (td, J = 6.8, 1.1 Hz, 1H), 2.97 (m, 1H), 1.35 (d, J = 4 Hz, 6H) **^{13}C NMR** (126 MHz, $CDCl_3$) δ (ppm): 154.13, 144.96, 125.43, 123.97, 117.03, 111.72, 107.43, 28.44, 22.51 **HRMS** (ESI): Anal. Calcd. For HRMS m/z (ESI): calcd. for [$C_{10}H_{12}N_2$] [M^+] H^+: 161.1073 Found: 161.1076

4.References:

[1] X. Q. Yu, M. L. Feng, S. Q. Li, H. Z. He, L. Y. Xi, S. Y. Chen, Green Chem., **2019**, 21, 1619-1624.

[2] D. C. Mohan, S. N. Rao, C. Ravi, S. Adimurthy, Asian J. Org. Chem., **2014**, 3, 609-613.

[3] Z. H. Ren, M. N. Zhao, Y. K. Yi, Y. Y. Wang, Z. H. Guan, Synthesis, **2016**, 48, 1920-1926.

[4] K. Godugu, C. G. R. Nallagondu, Journal of Heterocyclic Chemistry, **2021**, 58, 250-259.

[5] X. Meng, Y. M. Wang, C. Y. Yu, P. Q. Zhao, RSC Advances, **2014**, 4, 27301-27307.

[6] S. Kundu, B. Basu, RSC Advances, **2015**, 5, 50178-50185.

[7] Y. Liu, Z. Q. Wang, T. Lei, Y. L. Li, ChemistrySelect, **2022**, 7.

[8] M. H. Shinde, U, A. Kshirsagar, Green Chemistry, **2016**, 18, 1455-1458.

[9] Y. F. Zhang, Z. K. Chen, W. L. Wu, Y. H. Zhang, W. P. Su, Journal of Organic Chemistry, **2013**, 78, 12494-12504.

5. ^{1}H NMR and ^{13}C NMR spectra of the synthesized compounds (3aa-3de)

[3.1]2-phenylimidazo[1,2-a] pyridine(3aa) of ^{13}C and ^{1}H NMR

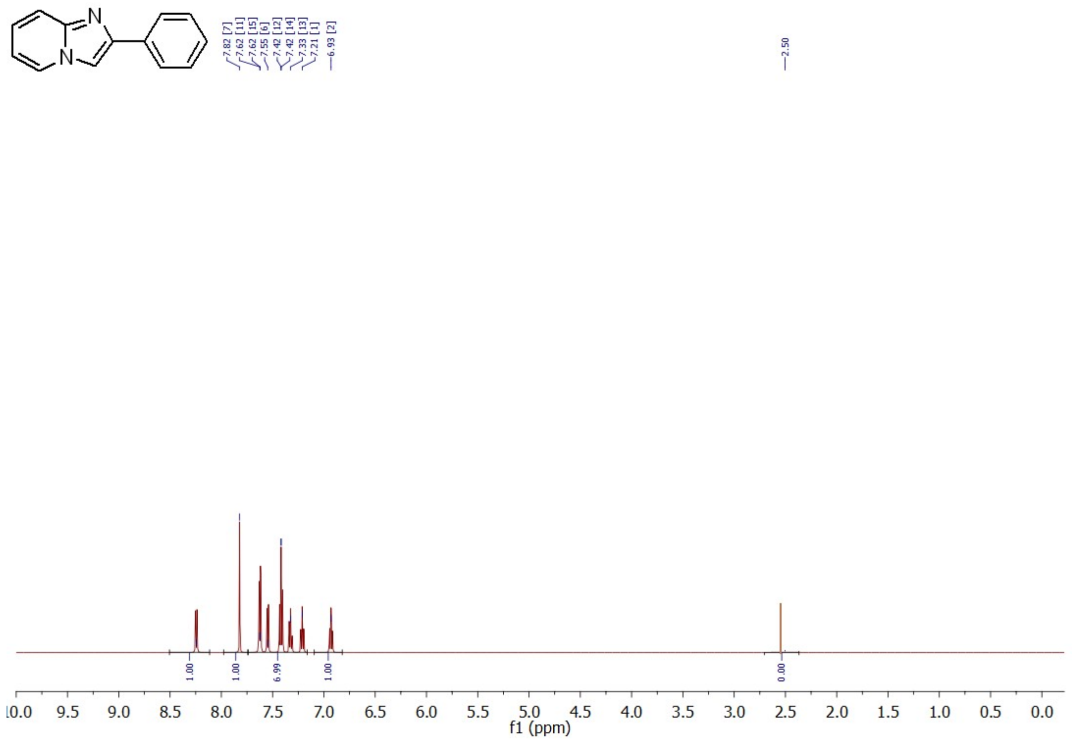

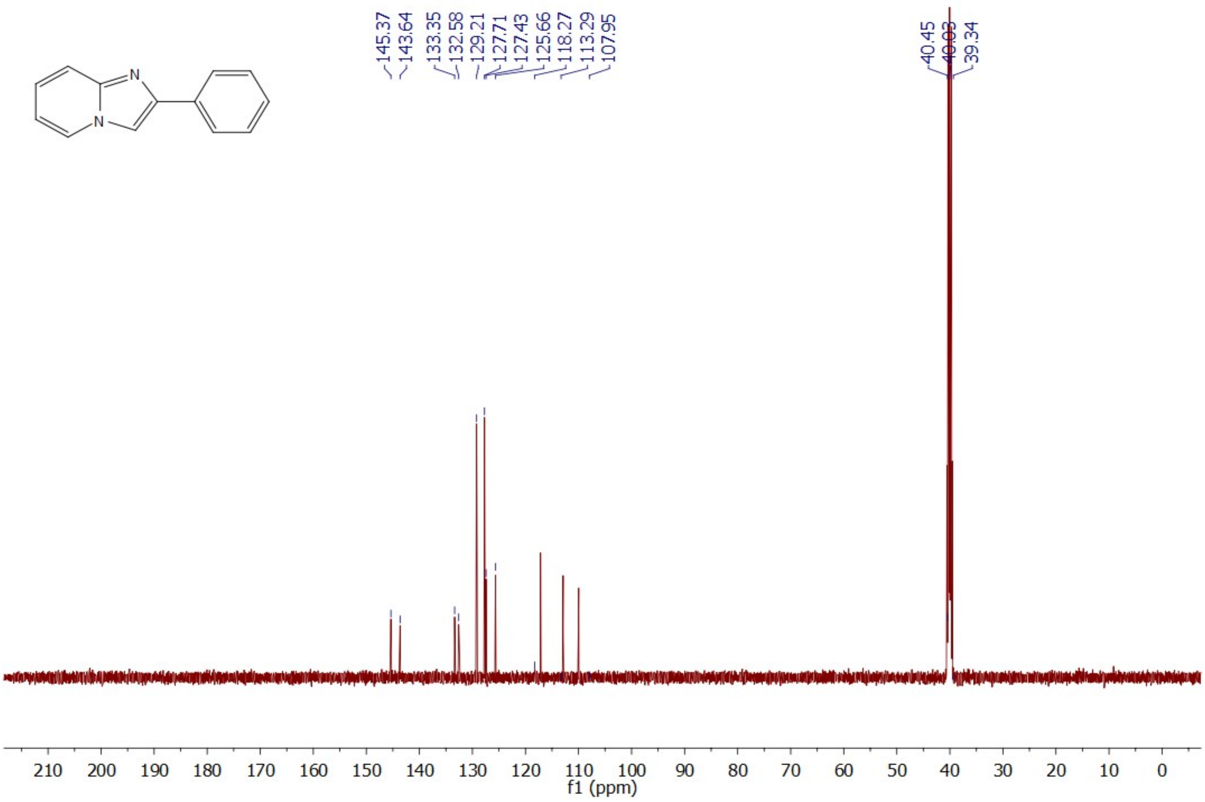

[3.2], ^{1}H and ^{13}C NMR of the2-(p-Tolyl)imidazo[1,2-a]pyridine(3ba) .

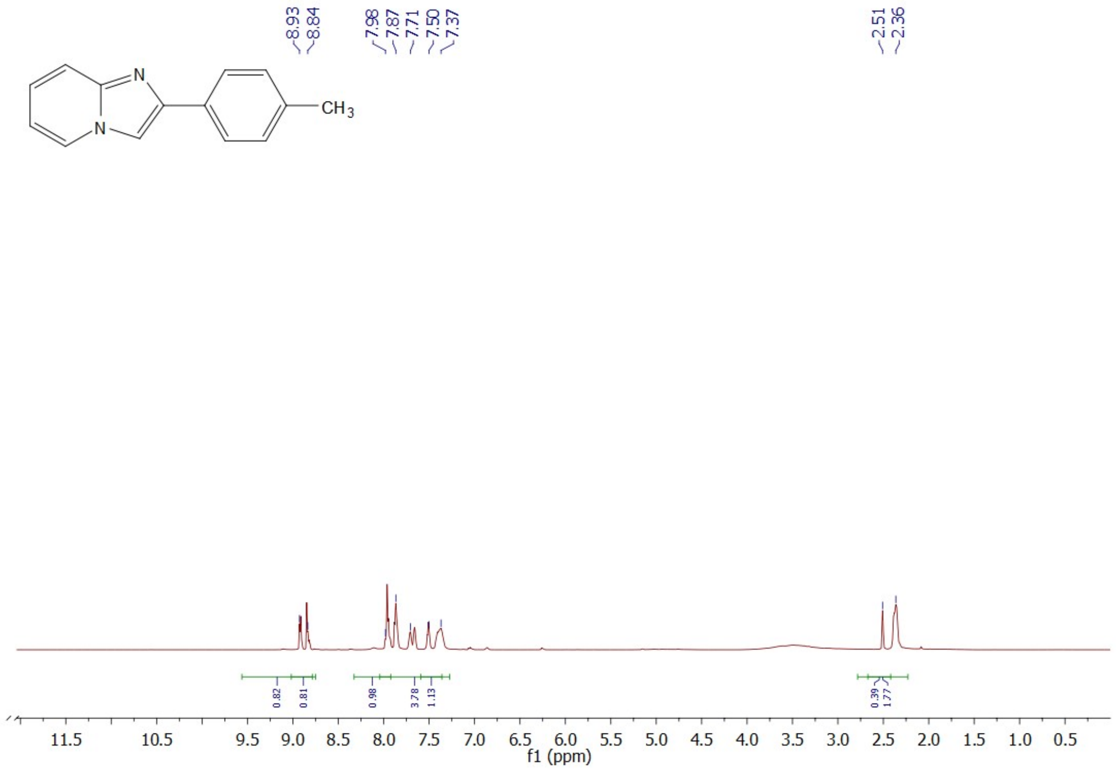

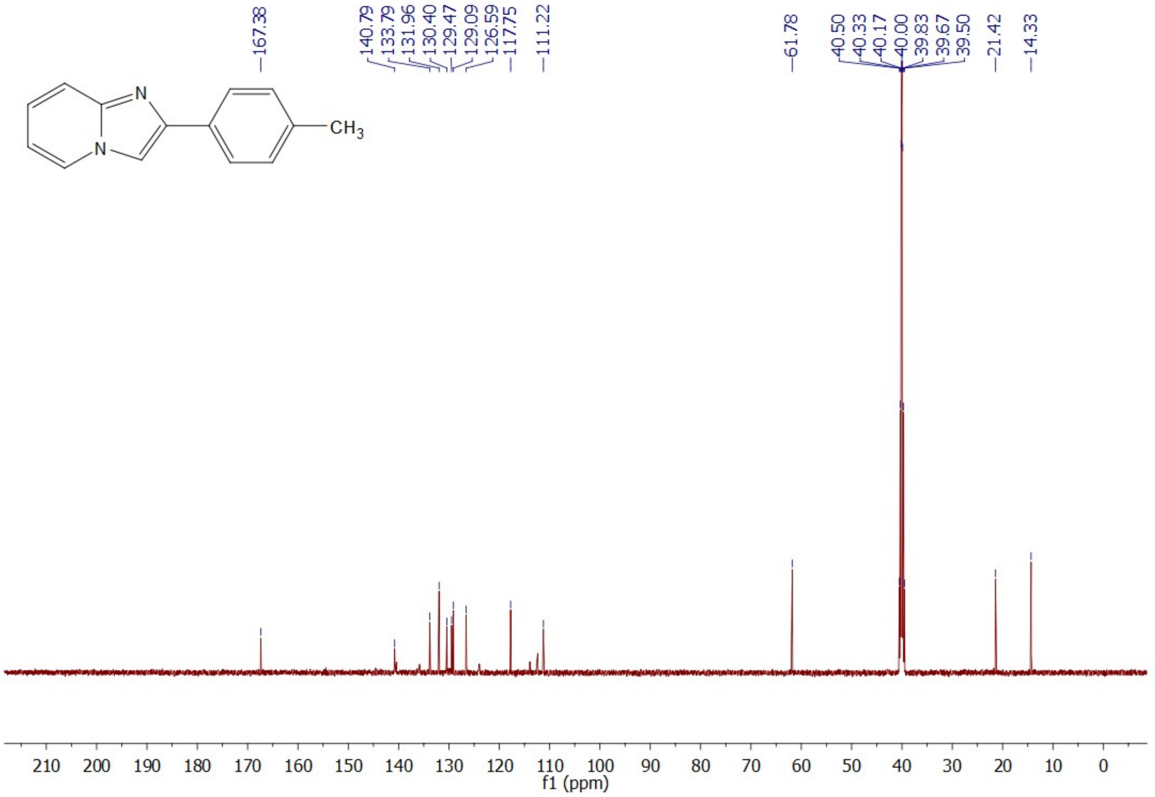

[3.3] ^{1}H and ^{13}C NMR of the 2, -(4-Methoxyphenyl) imidazo[1,2-a]pyridine(3ca)

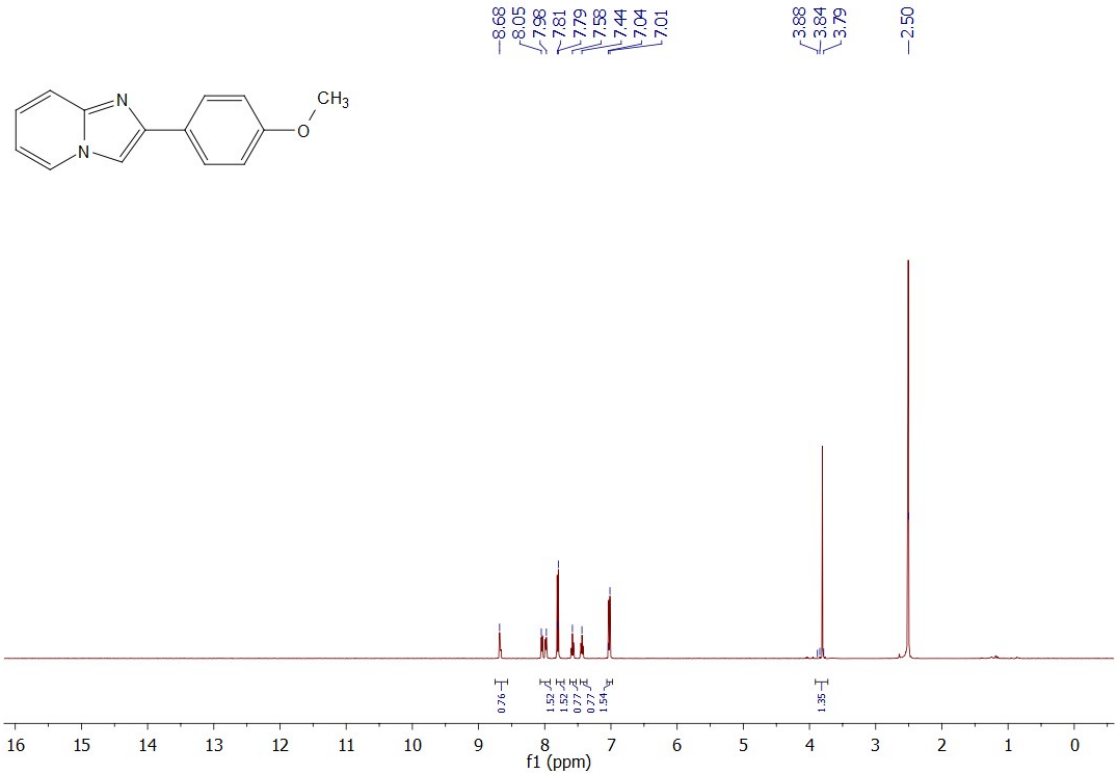

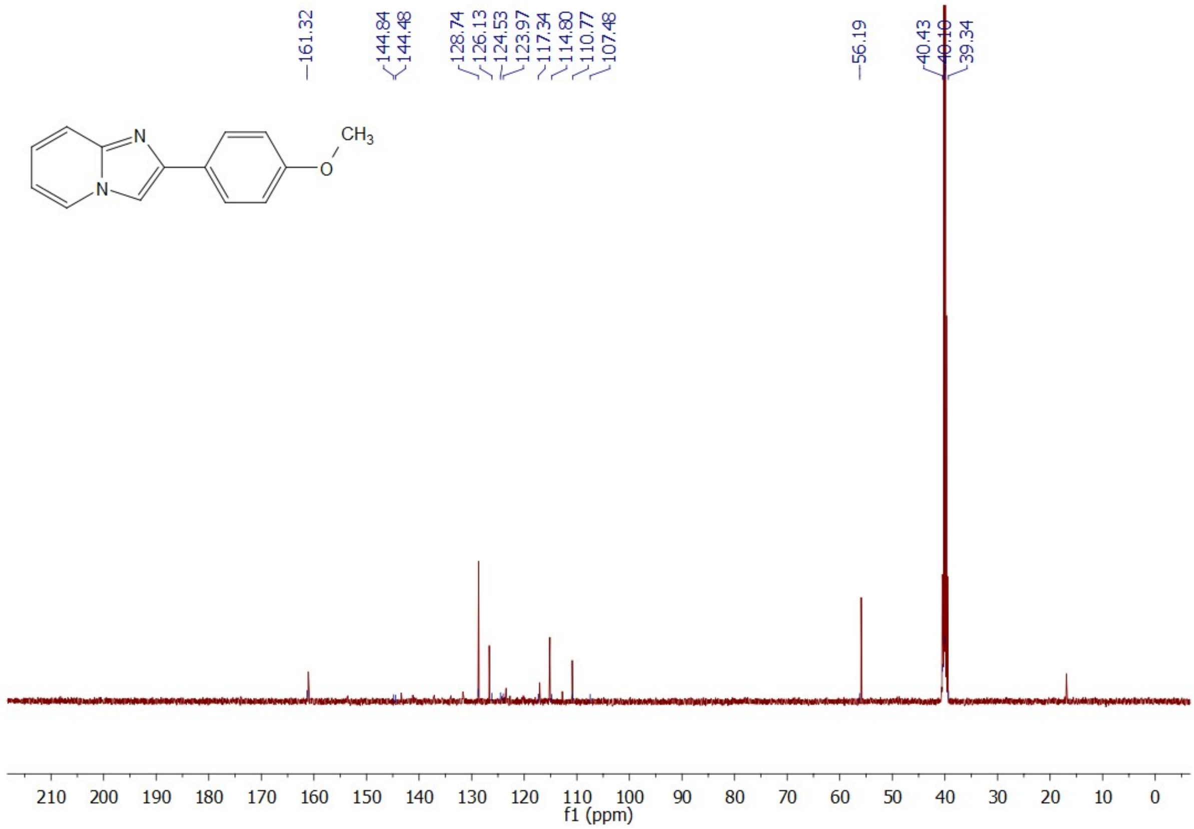

[3.4], ^{1}H and ^{13}C NMR of the 2-(4nitrophenyl)imidazo[1,2-a]pyridine (3da) .

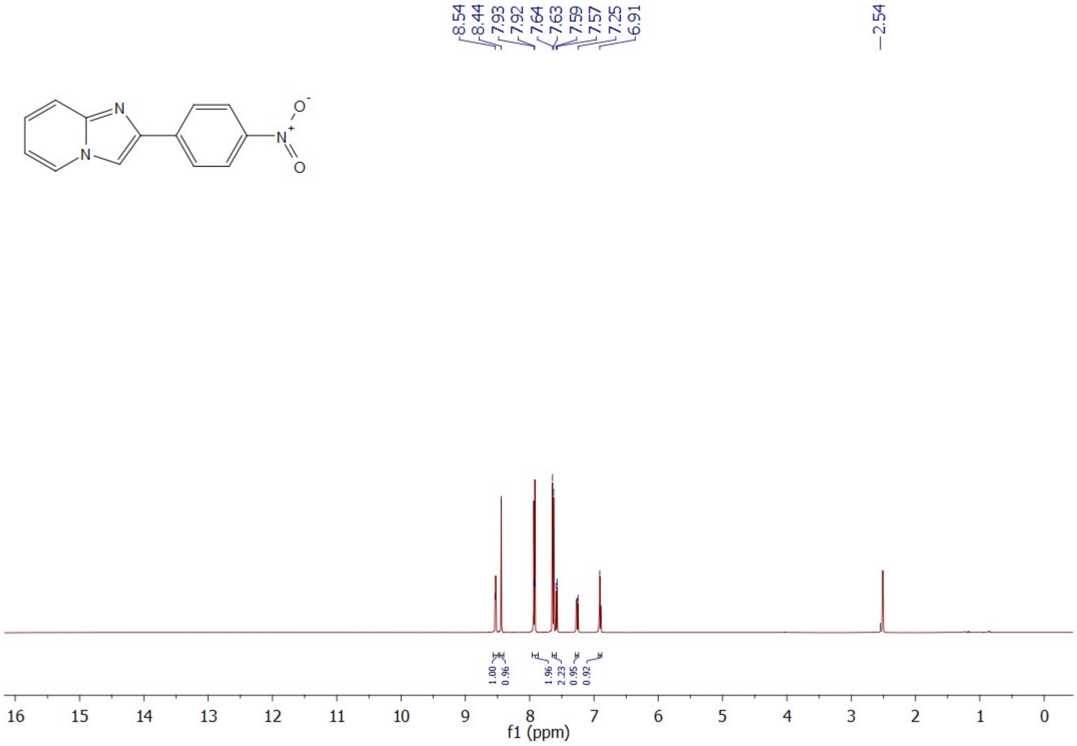

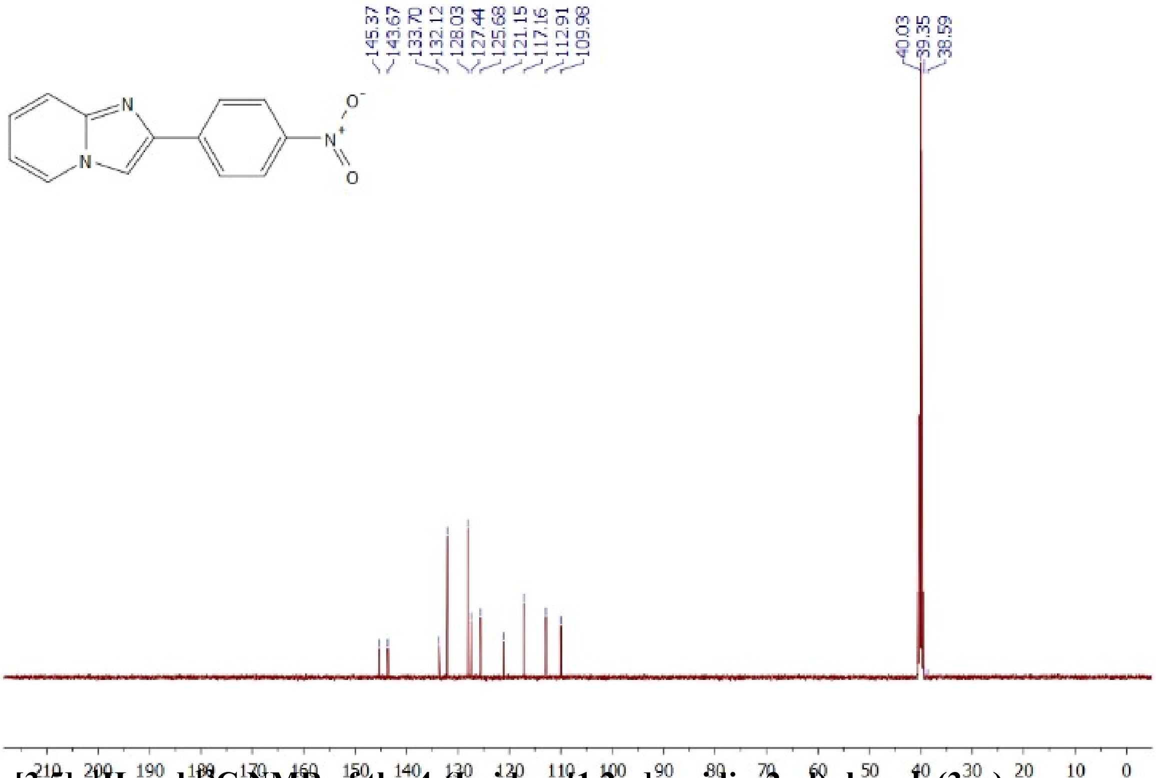

[3.5], ^{1}H and ^{13}C NMR of the 4-(Imidazo[1,2-a]pyridin-2-yl)phenol ,(3ea) .

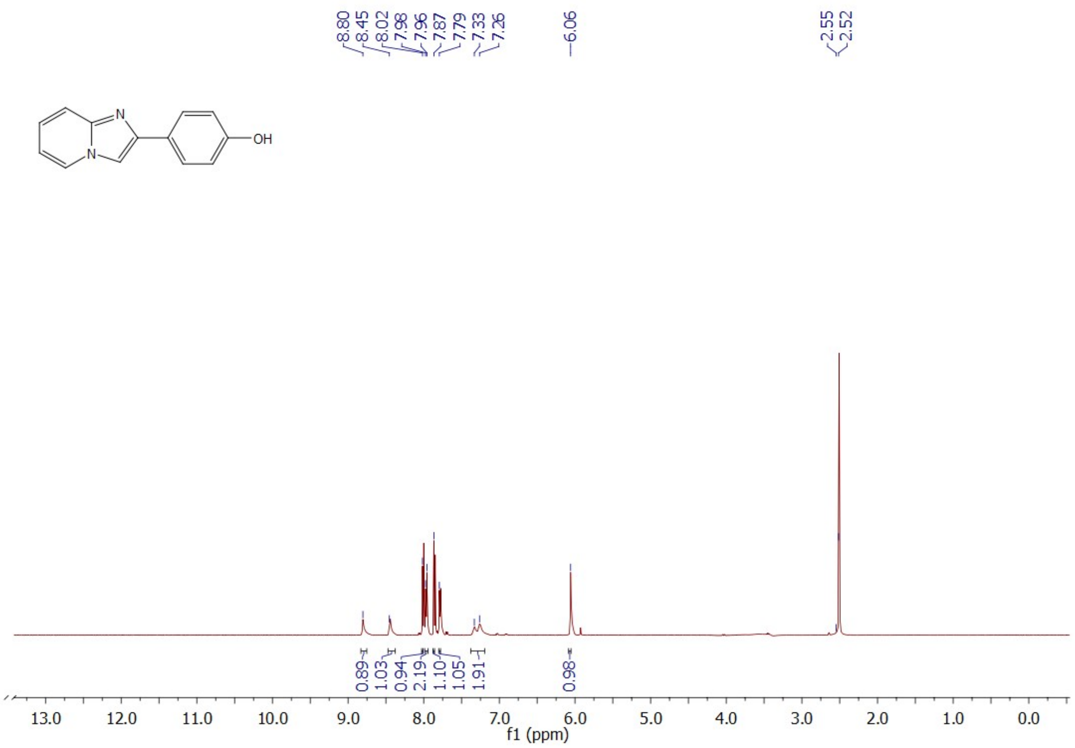

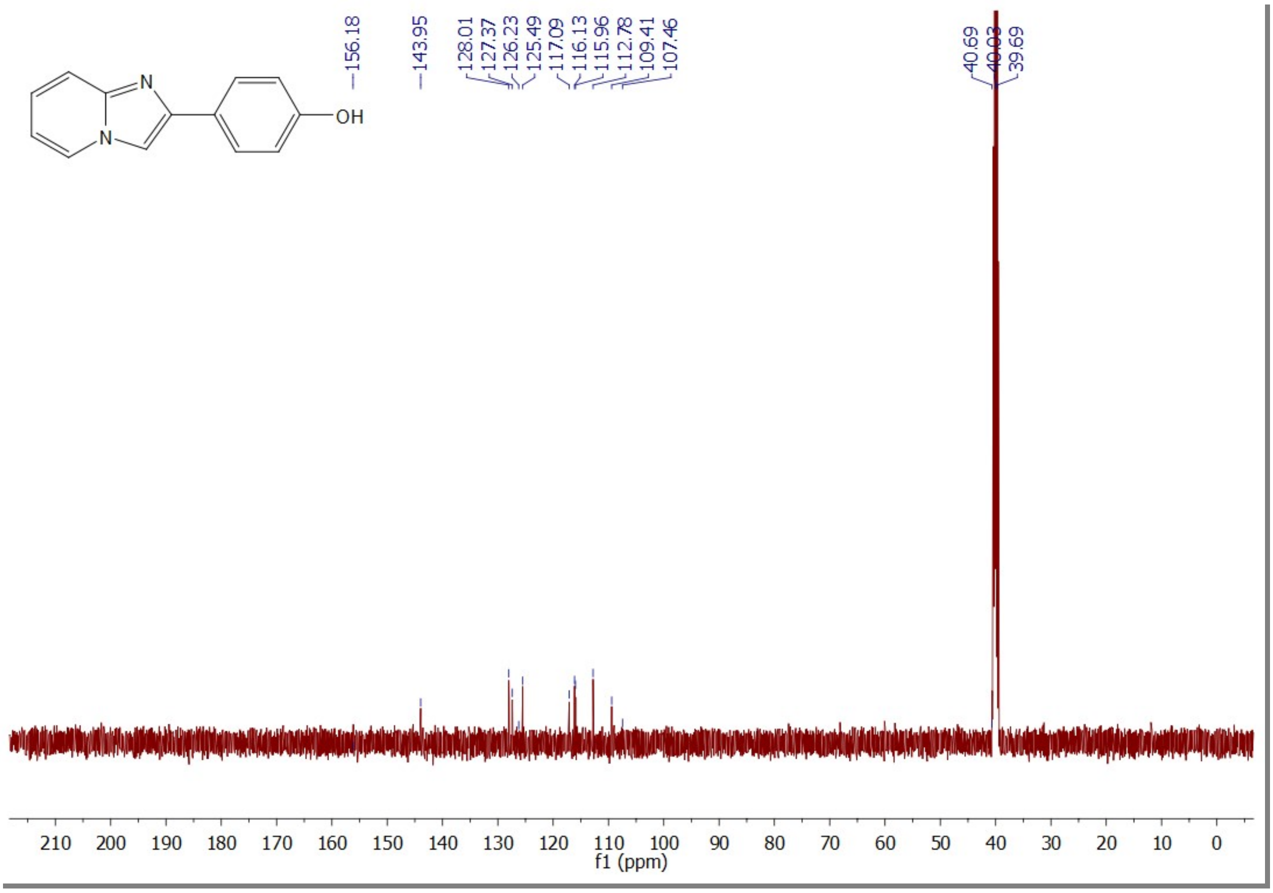

[3.6], ^{1}H and ^{13}C NMR of the 2-(4-Bromophenyl) imidazo[1,2- a]pyridine (3fa) .

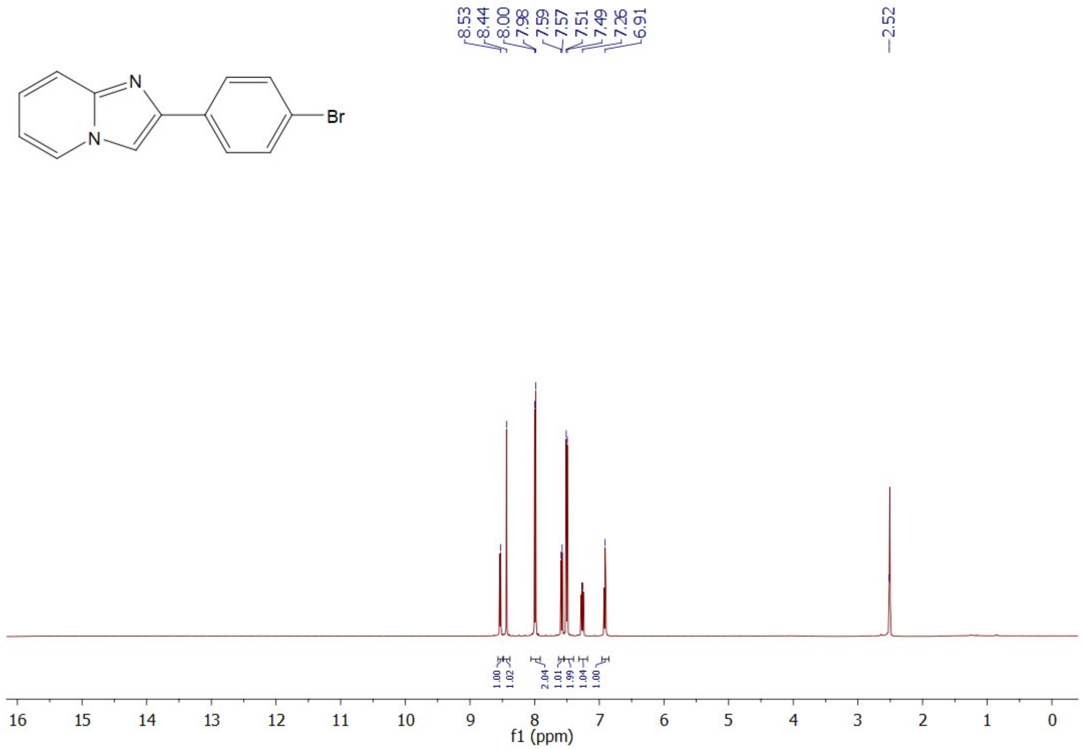
8.53
8.44
8.00
7.98
7.59
7.57
7.51
7.49
7.26
6.91
2.52
N
N
Br
16 15 14 13 12 11 10 9 8 7 6 5 4 3 2 1 0
f1 (ppm)

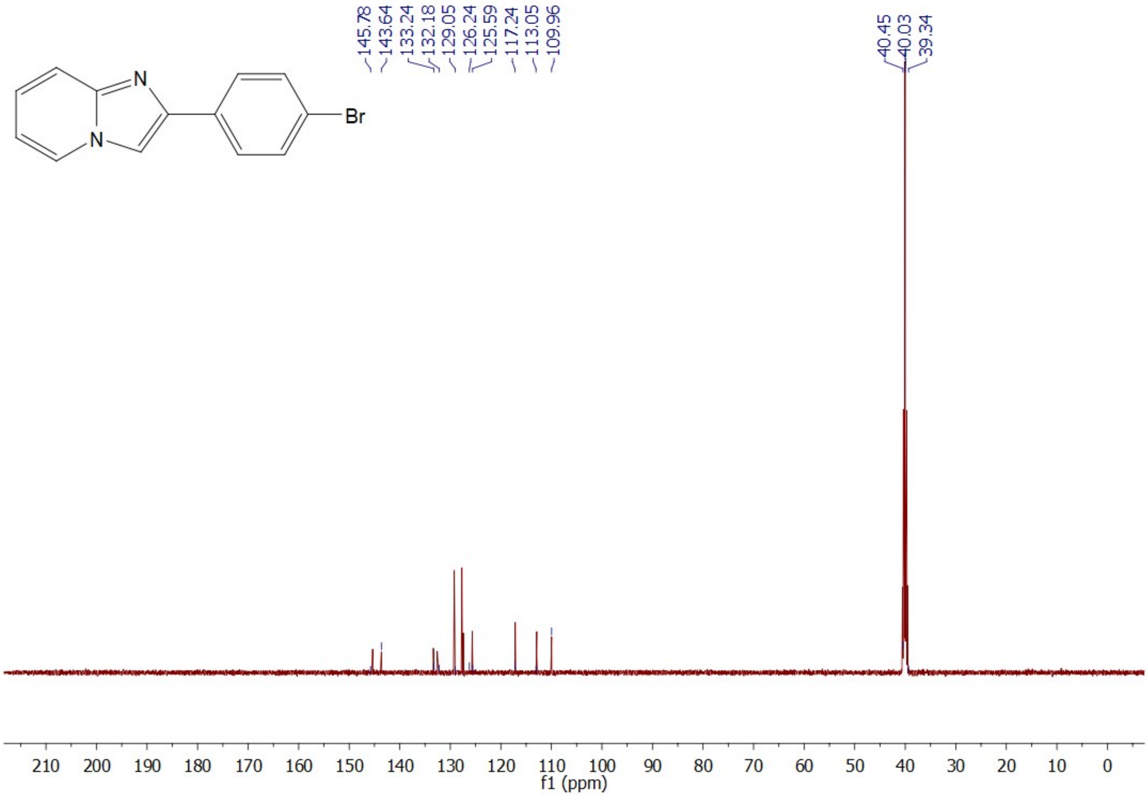
145.78
143.64
133.24
132.18
129.05
126.24
125.59
117.24
113.05
109.96
40.45
40.03
39.34
N
N
Br
210 200 190 180 170 160 150 140 130 120 110 100 90 80 70 60 50 40 30 20 10 0
f1 (ppm)

[3.7], ^{1}H and ^{13}C NMR of 2-(4-Fluorophenyl) imidazo[1,2-a] pyridine(3ga).

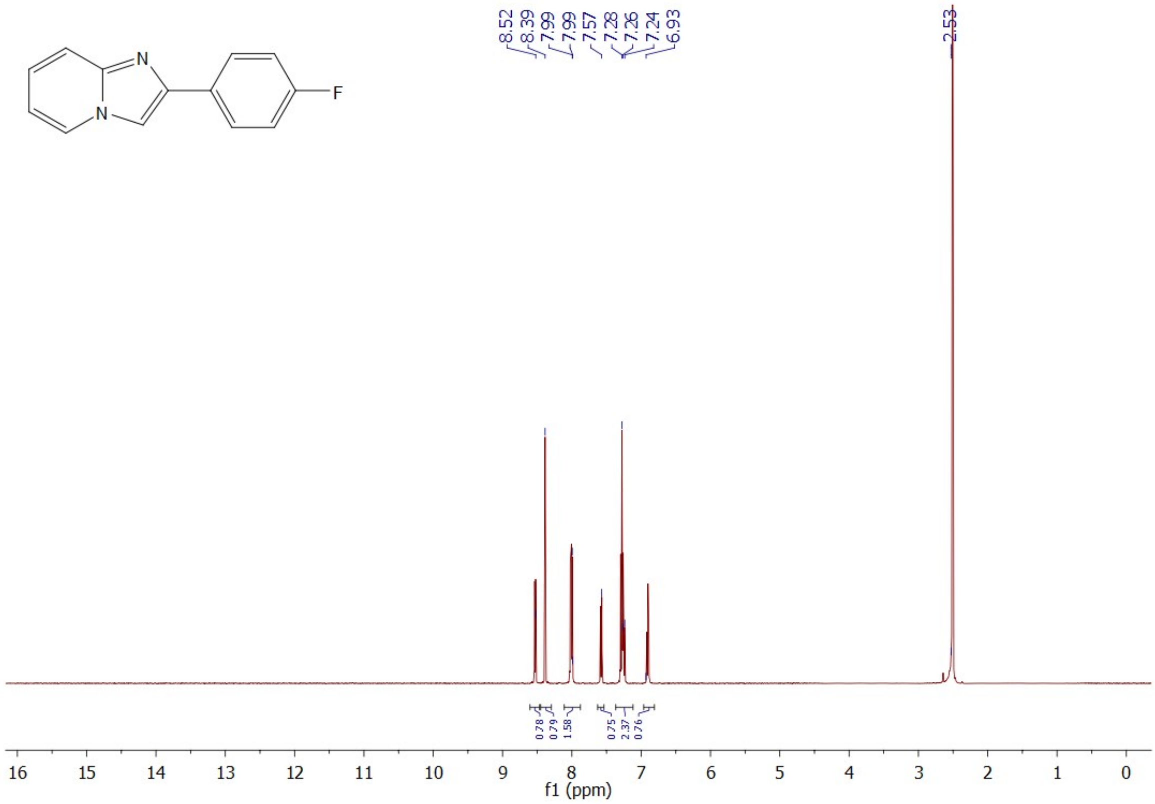

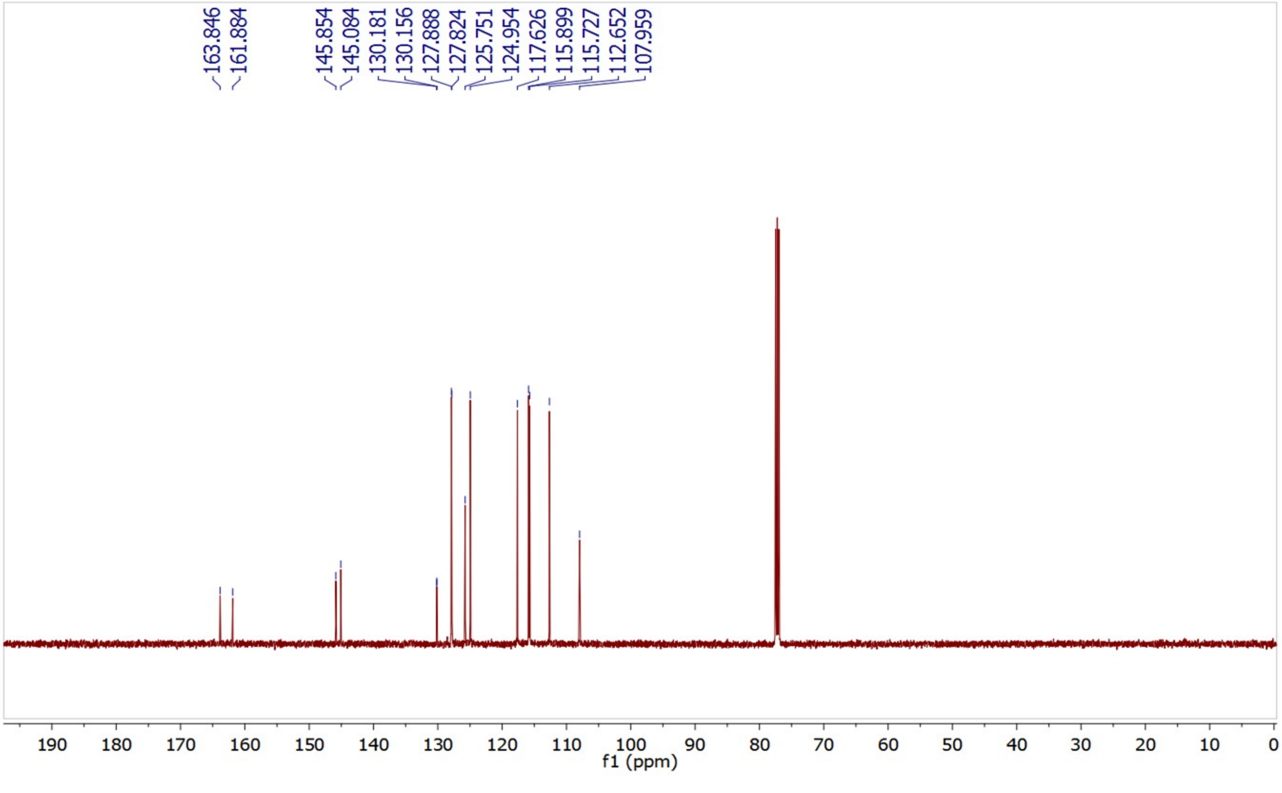

[3.7] ^{19}F NMR of 2-(4-Fluorophenyl) imidazo[1,2-a] pyridine(3ga):

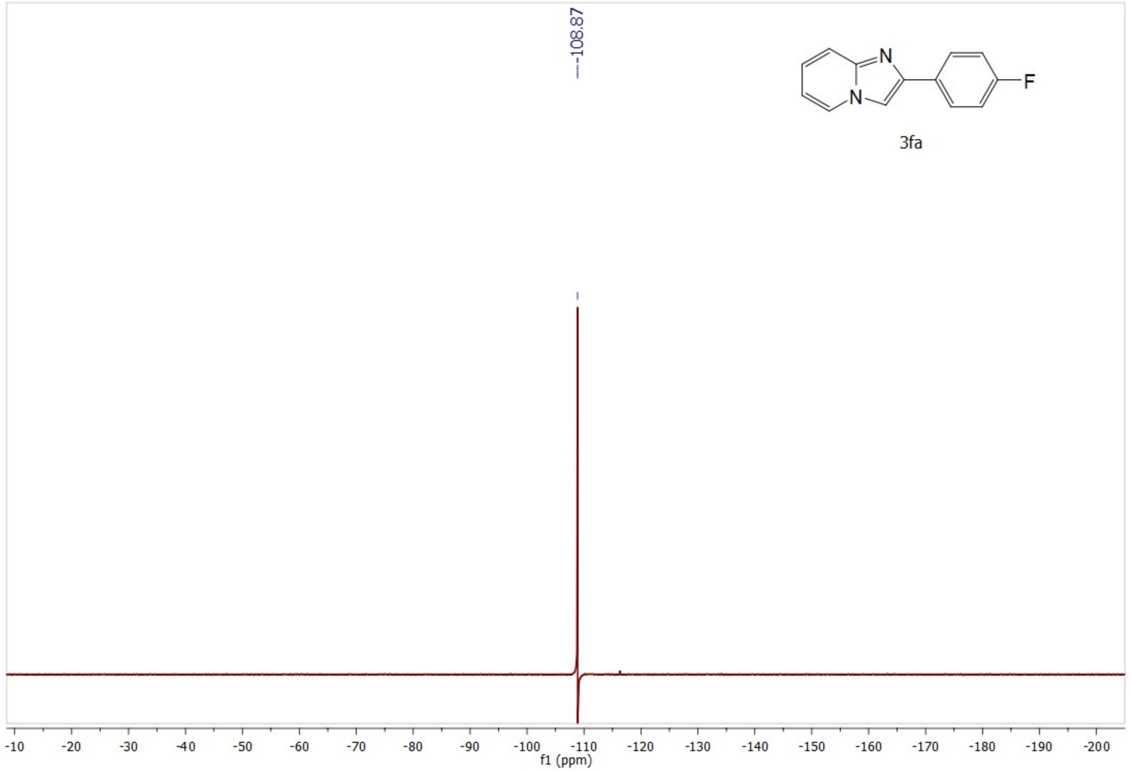

[3.7] HRMS of 2-(4-Fluorophenyl) imidazo[1,2-a] pyridine(3ga)

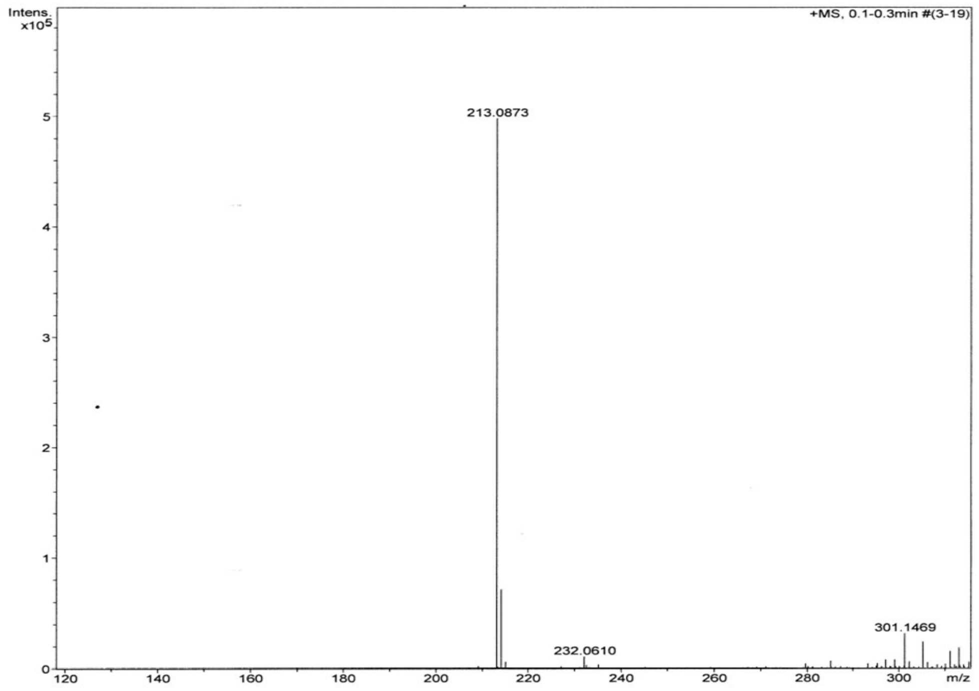

[3.8]^{1}H&^{13}CNMR of the 4-(imidazo[1,2-a]pyridine-2-yl)benzonitrile,of (3ha):

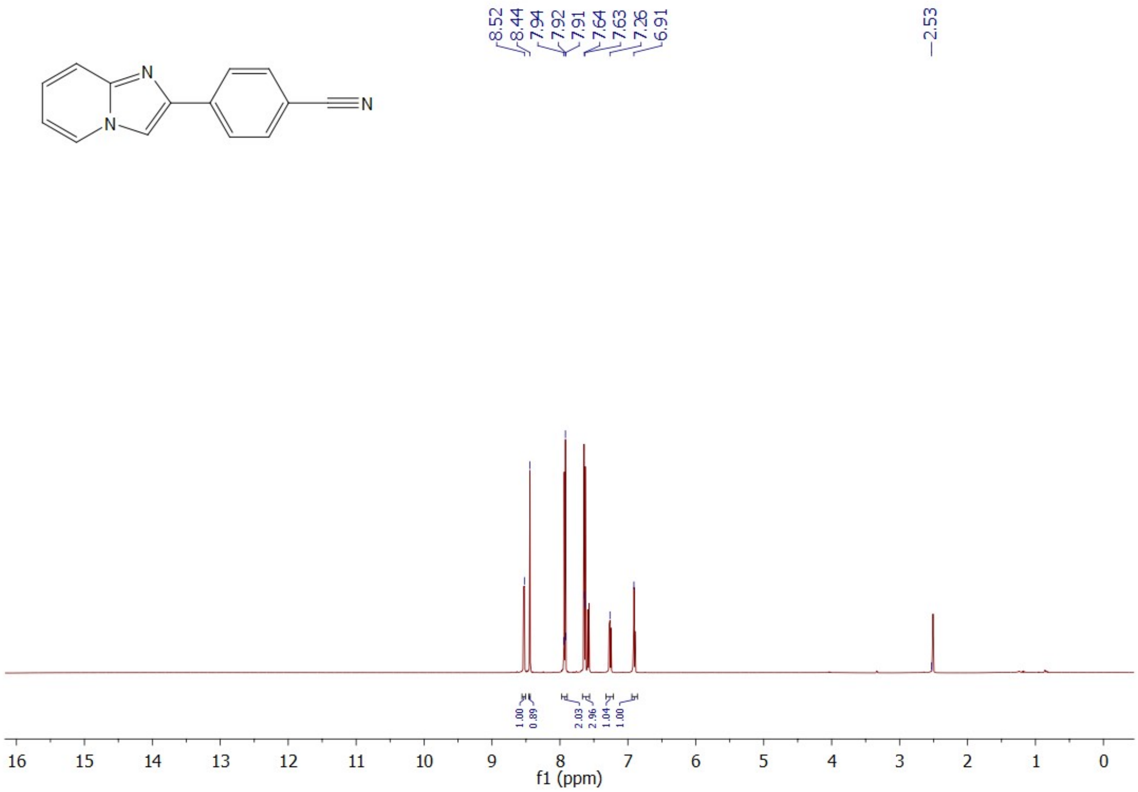

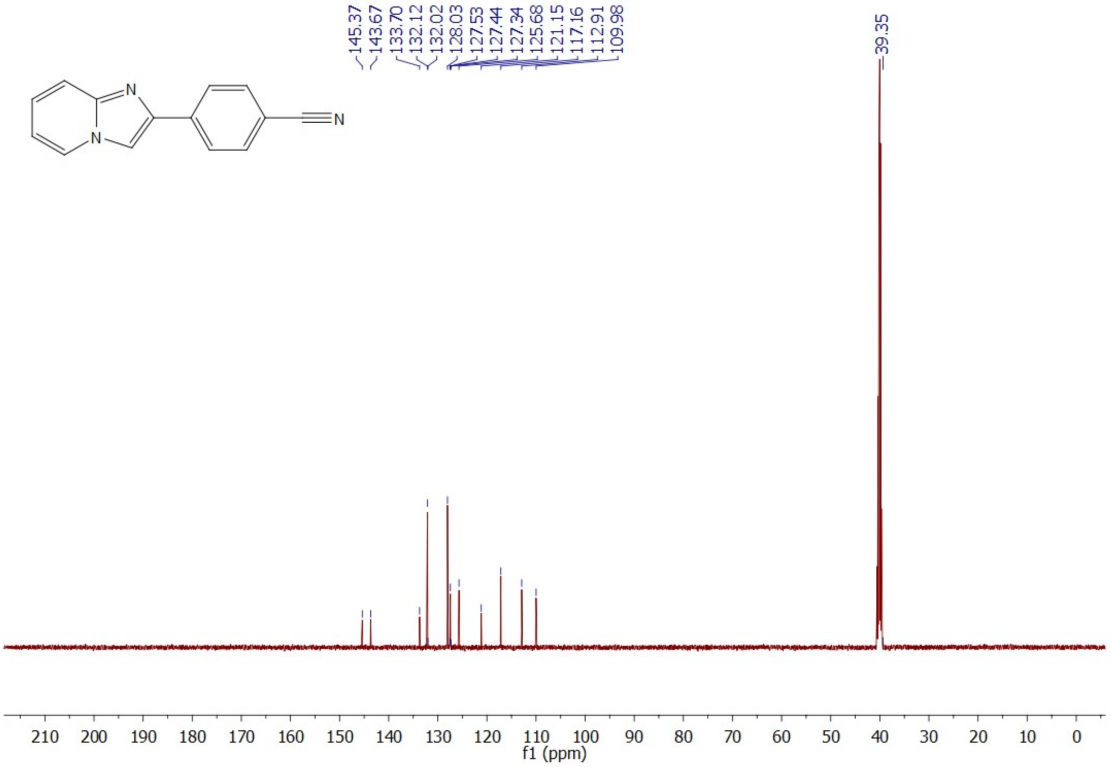

[3.9] (^{1}H & ^{13}C NMR), of the 2-(4-Chlorophenyl) imidazo[1,2-a] pyridine (3ia)

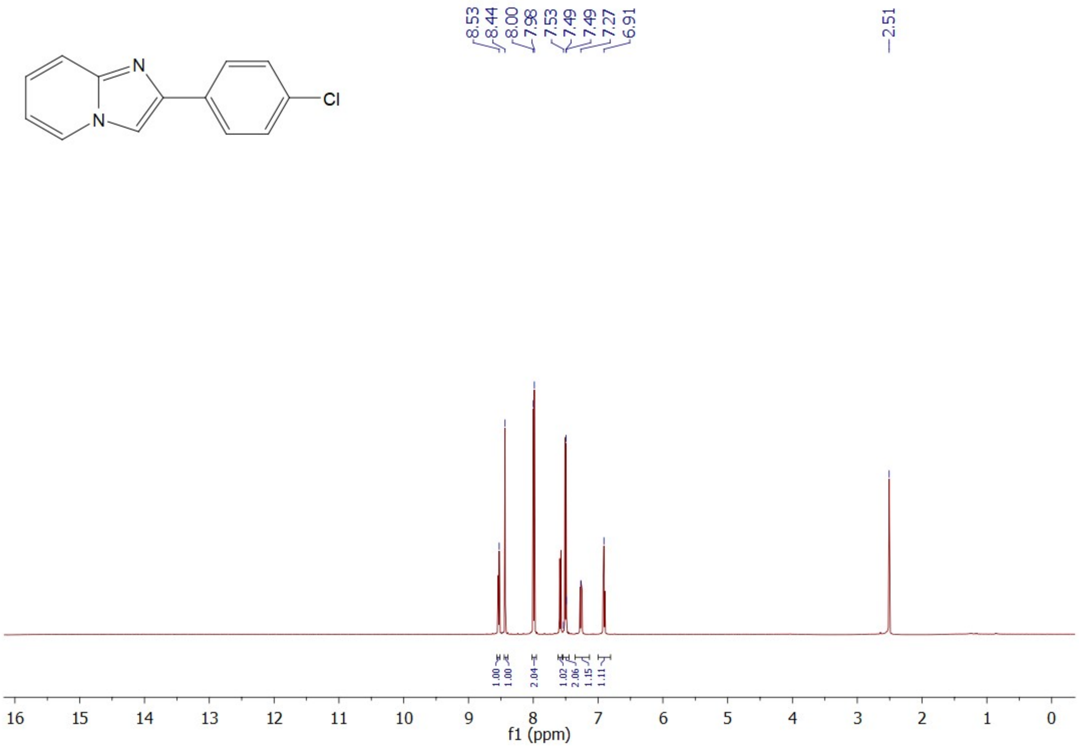

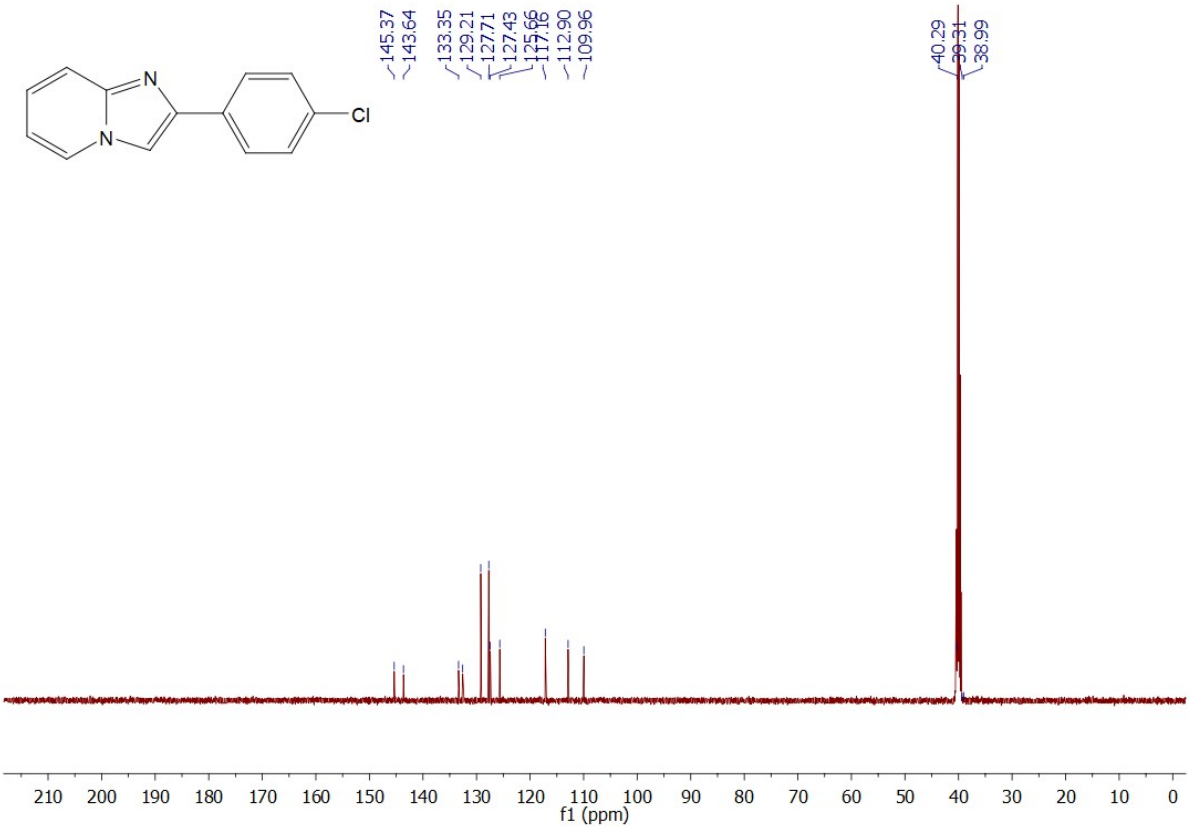

[3.10] ^{1}H and ^{13}C NMR of the 2-(Pyridin-2-yl) imidazo[1,2-a] pyridine (3ja)

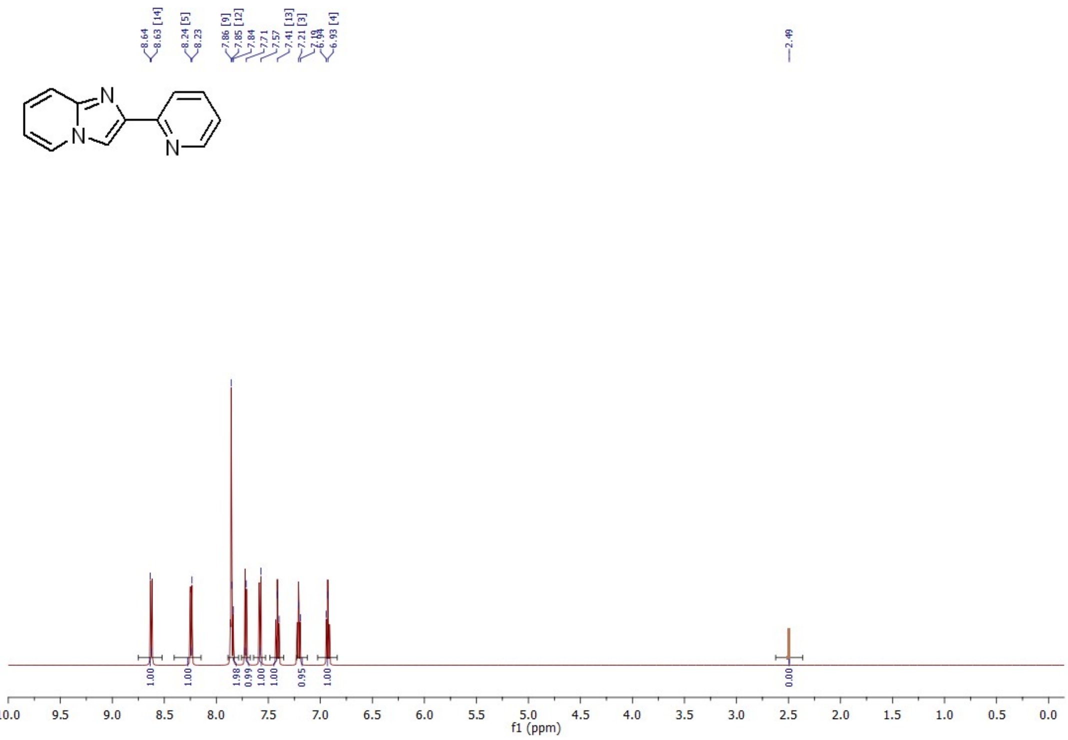
N
N
N
8.64
8.63 [14]
8.24 [5]
8.23
7.86 [9]
7.85 [12]
7.84
7.71
7.57
7.41 [13]
7.21 [3]
6.93 [4]
2.49
1.00
1.00
1.98
0.99
1.00
1.00
0.95
1.00
0.00
f1 (ppm)

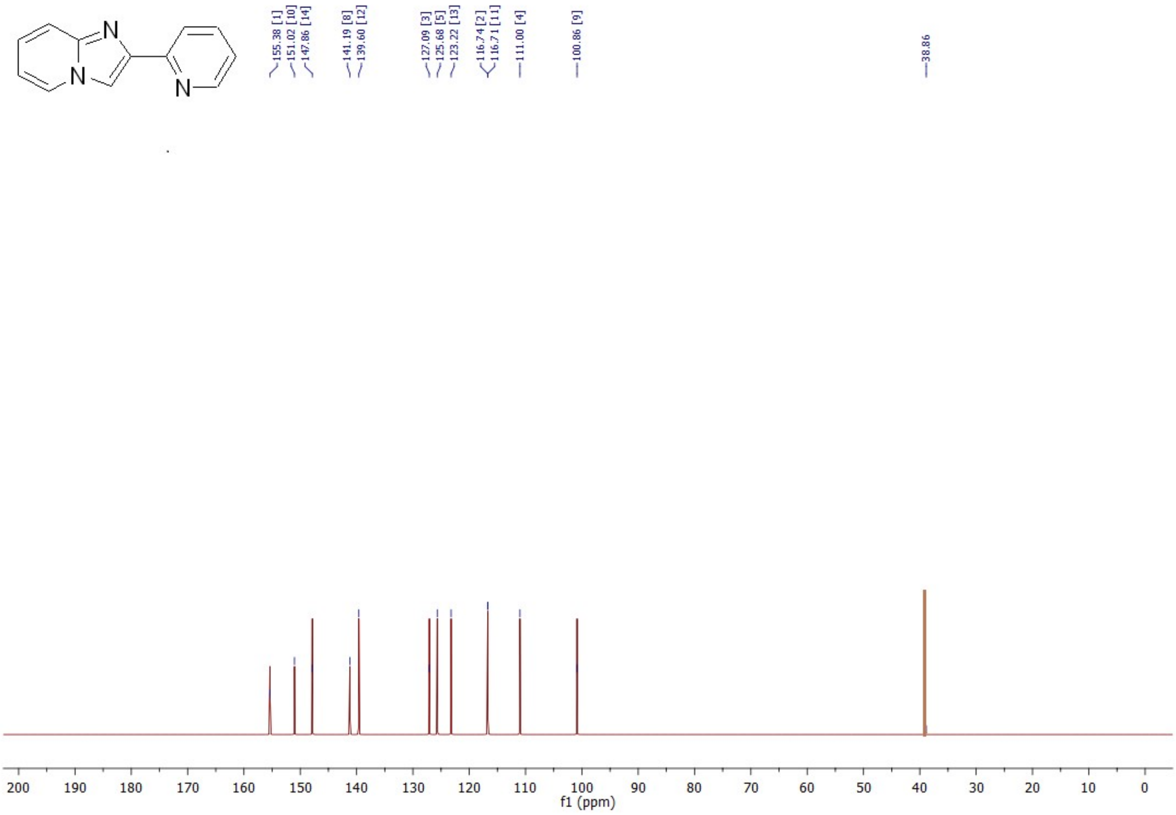
N
N
N
155.38 [1]
151.02 [10]
147.86 [14]
141.19 [8]
139.60 [12]
127.09 [3]
125.68 [5]
123.22 [13]
116.74 [2]
116.71 [11]
111.00 [4]
100.86 [9]
38.86
f1 (ppm)

[3.11] (^{1}H and ^{13}C NMR of the)2-(Thiophen-2-yl) imidazo[1,2-a] pyridine (3ka)

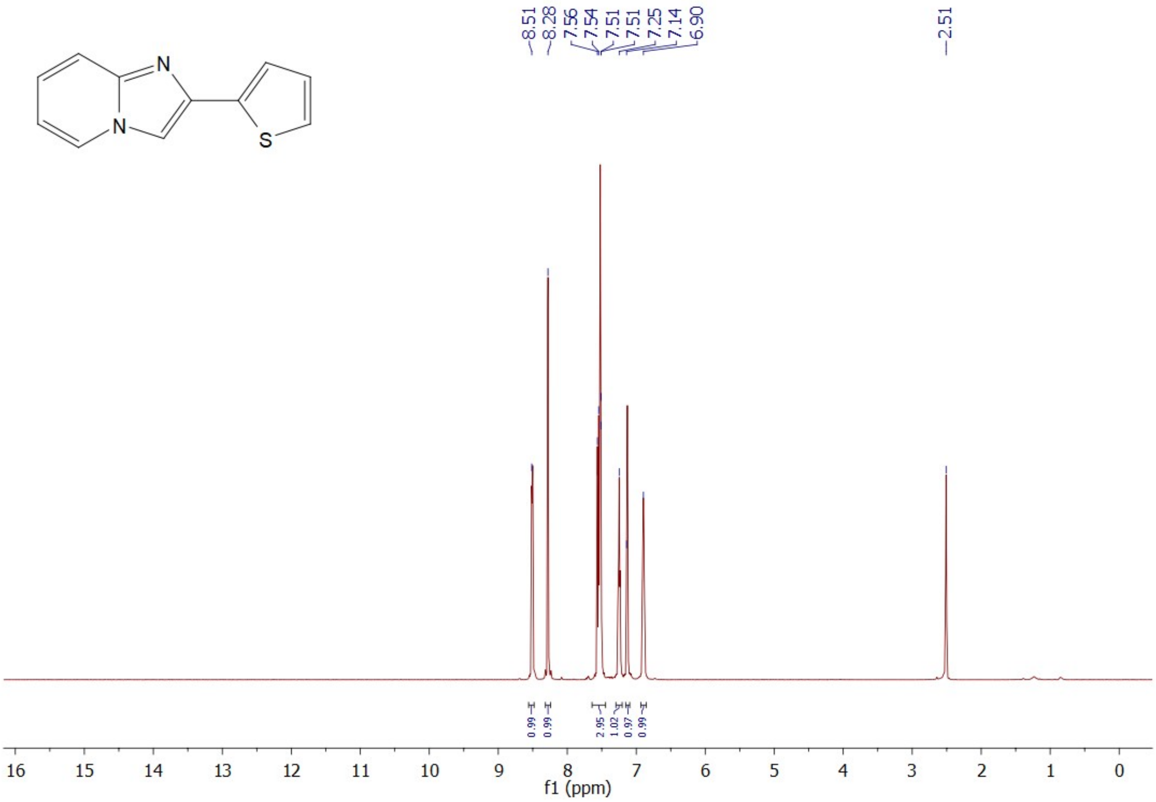

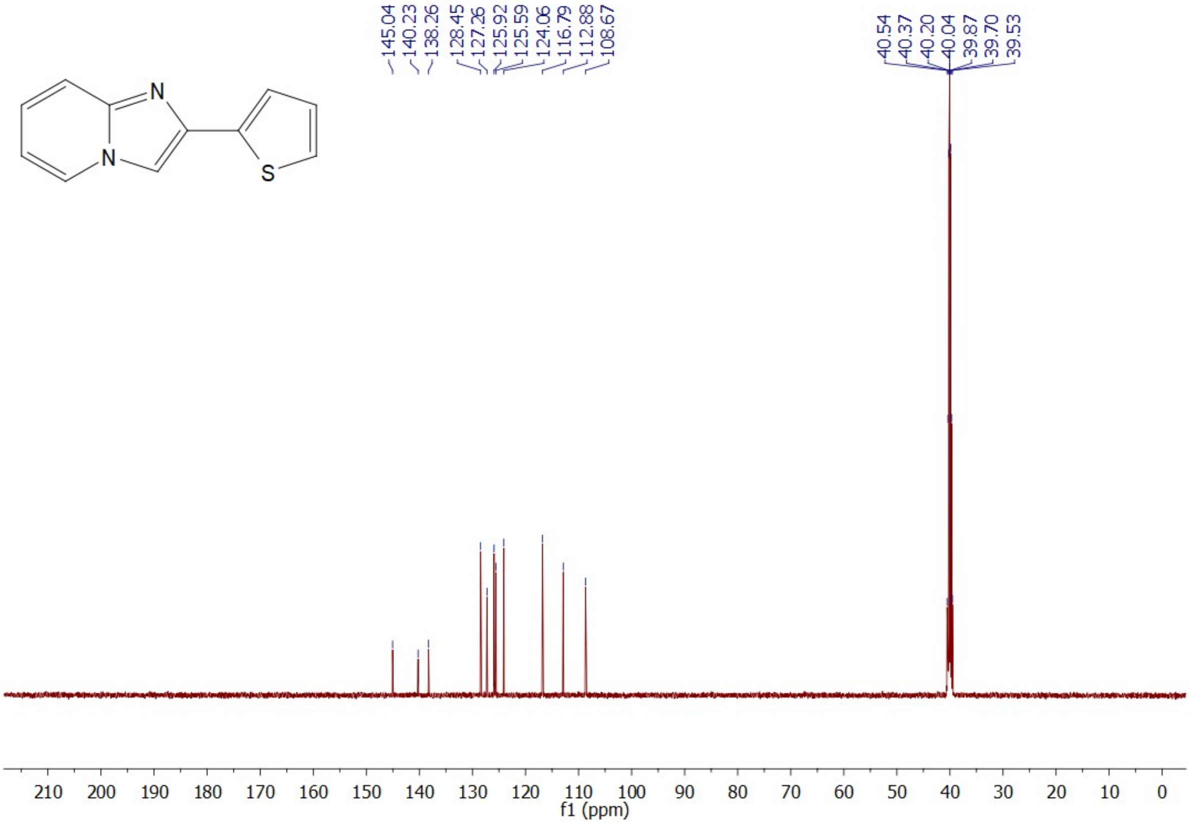

[3.12], (^{1}H and ^{13}C NMR) of the 2-(Naphthalen-1-yl)imidazo[1,2-a]pyridine(3la)

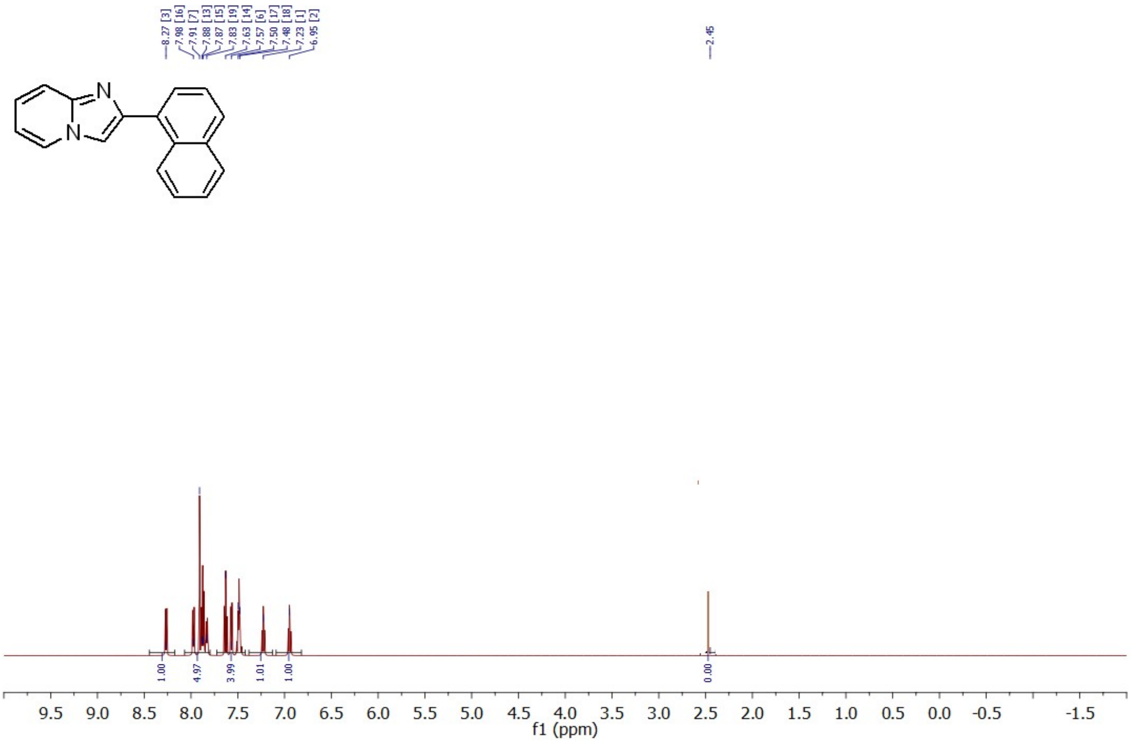

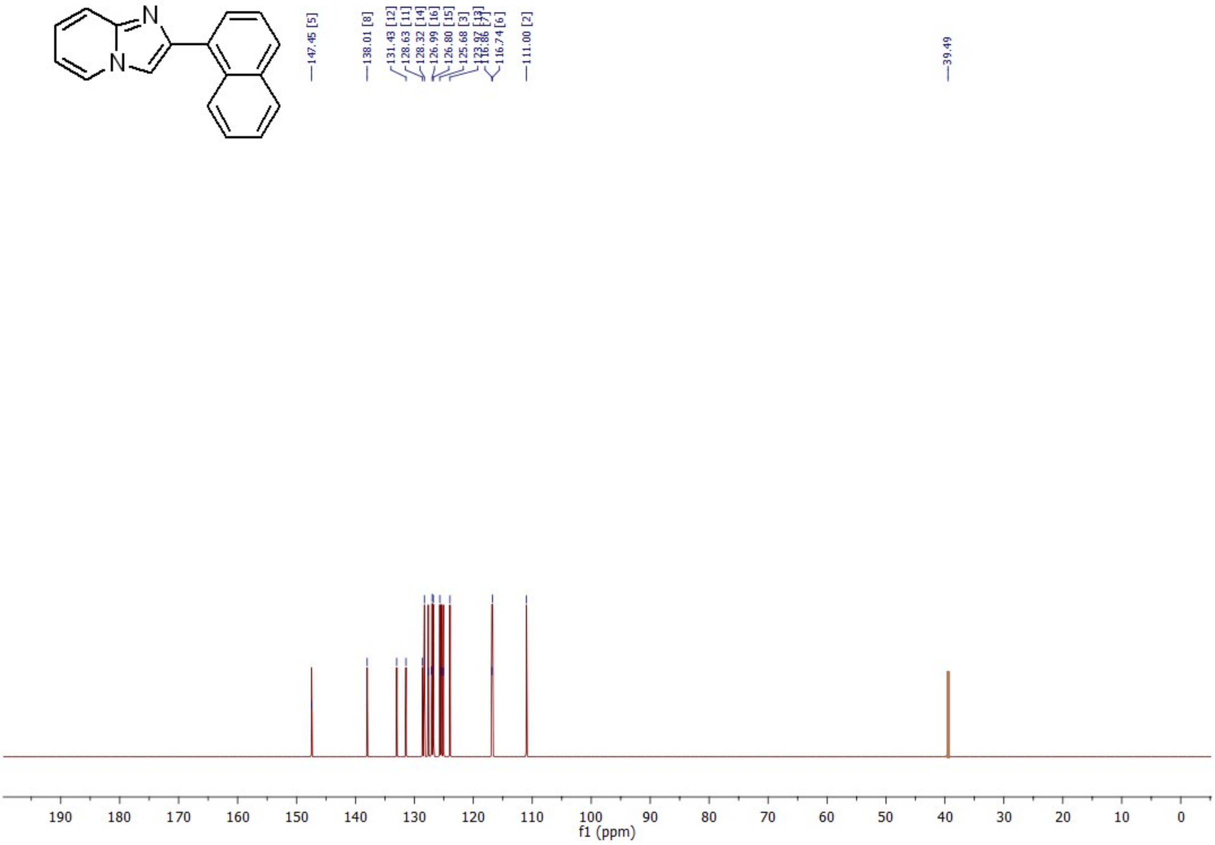

[3.13] (^{1}H & ^{13}C NMR), of the 8-Methyl-2-phenylimidazo[1,2-a]pyridine (3ab)

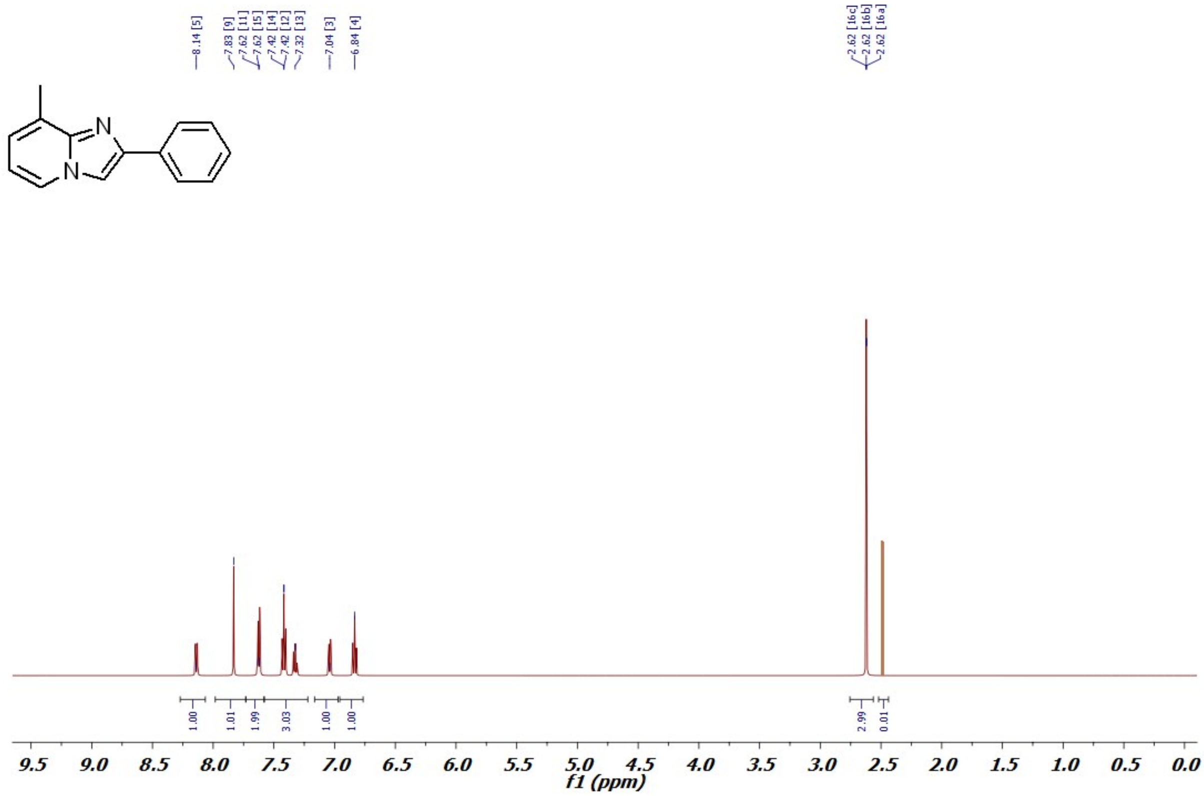

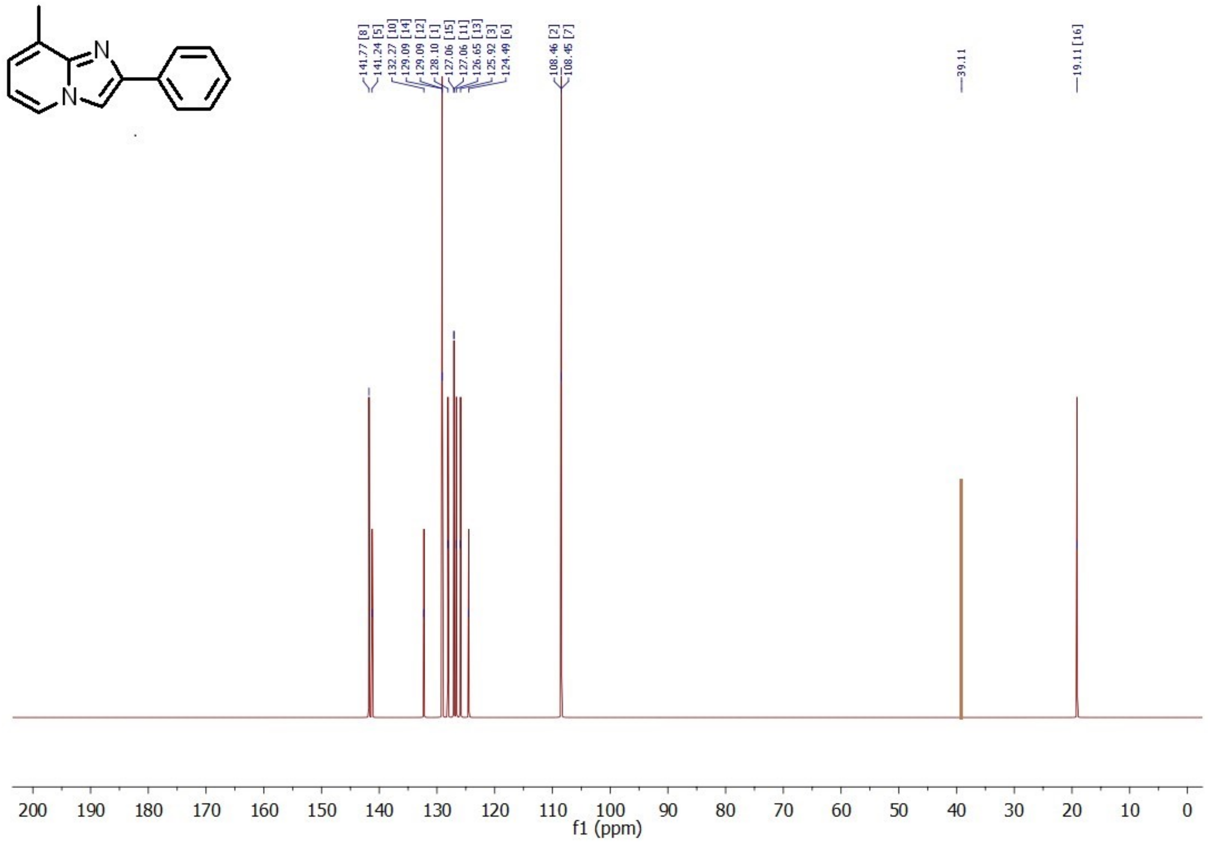

[3.14], (^{1}H &^{13}CNMR) of the 2-(4-Methoxyphenyl)-8-methylimidazo[1,2-a]pyridine(3cb)

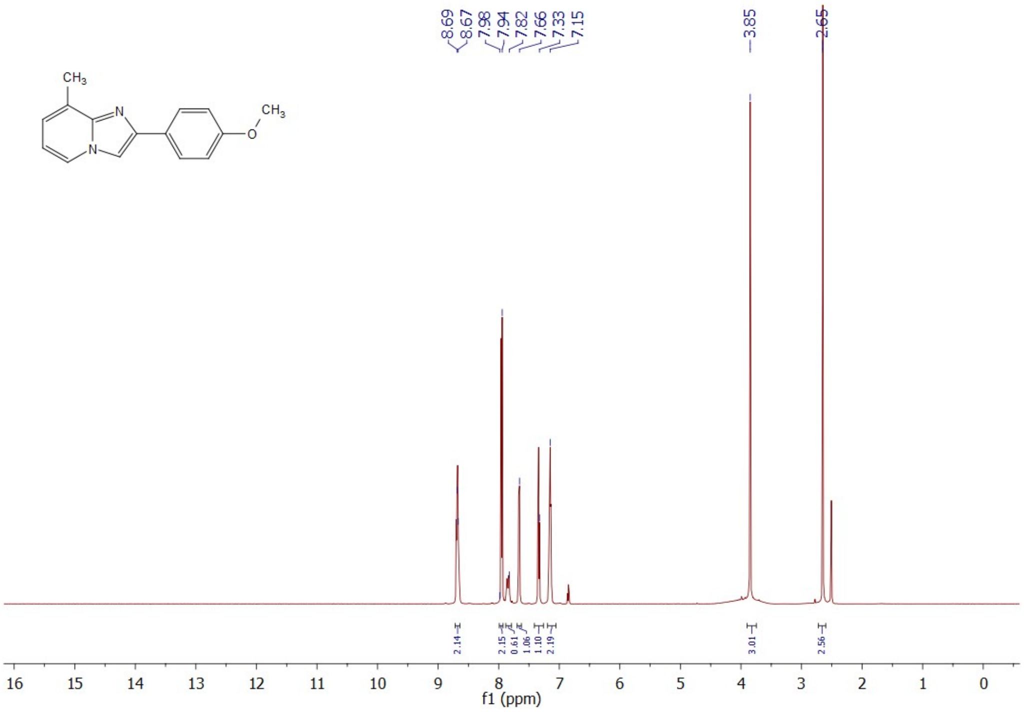

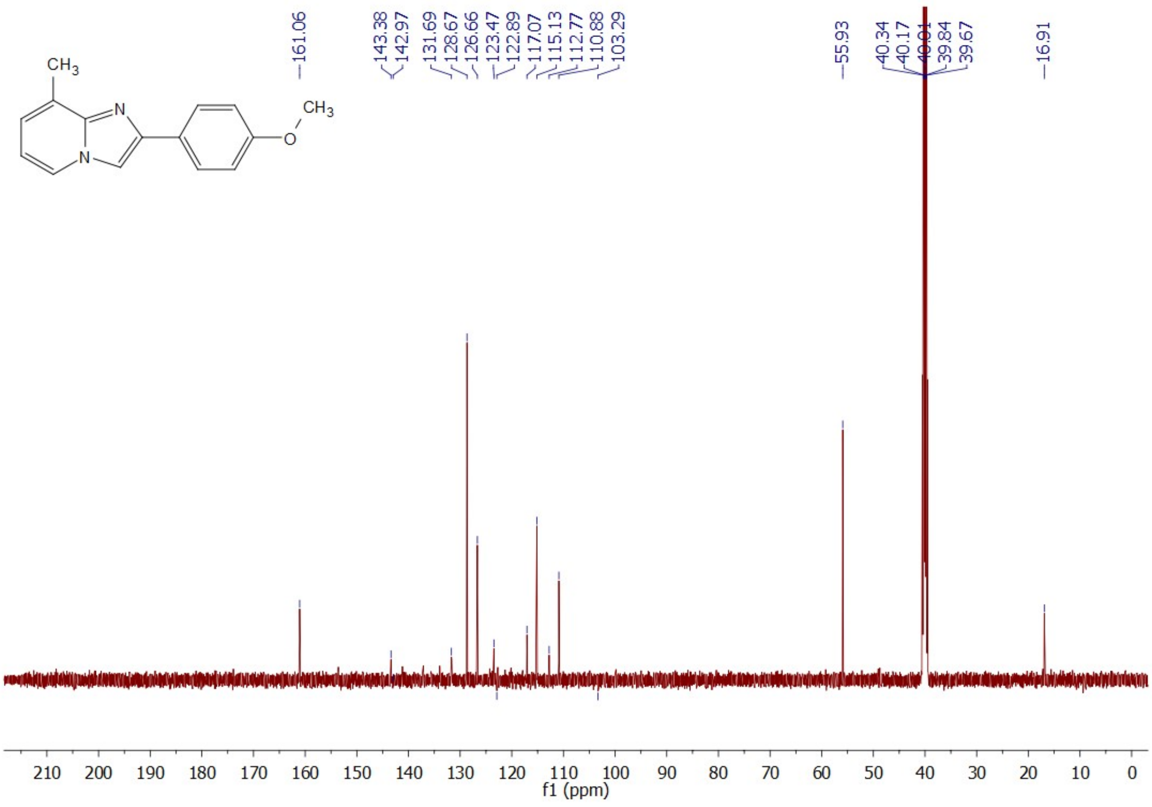

[3.15], (^{1}H and ^{13}CNMR) of the 8-Methyl-2-(3-nitrophenyl)imidazo[1,2-a]pyridine, (3db)

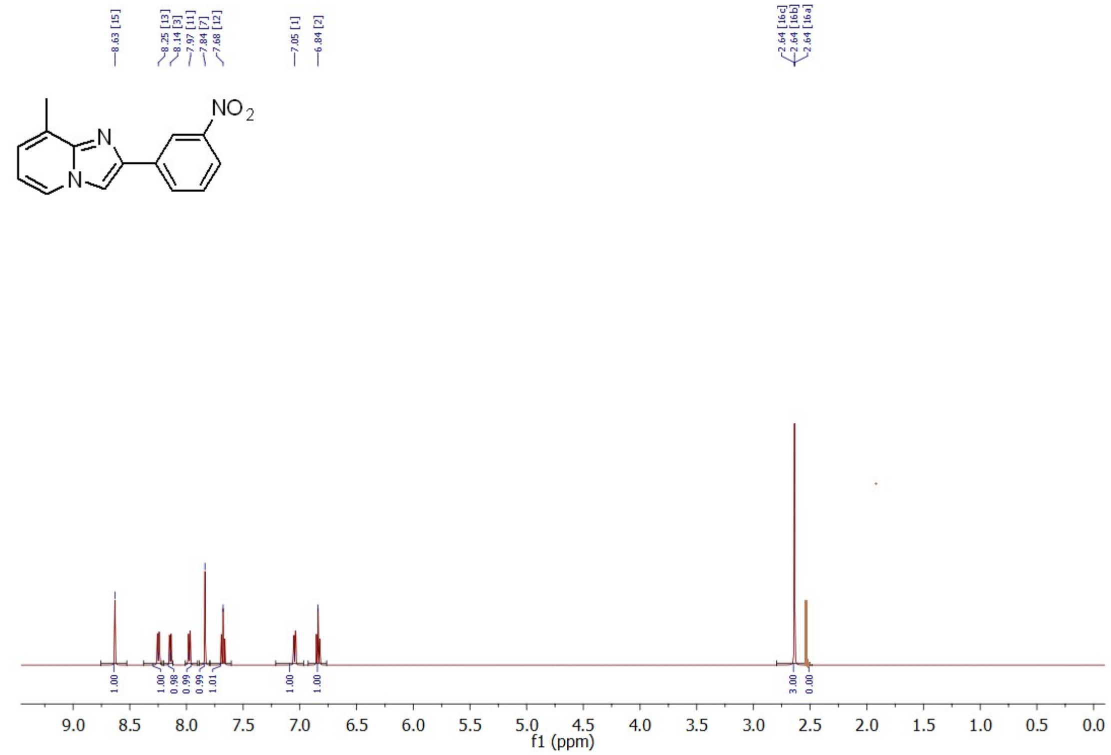

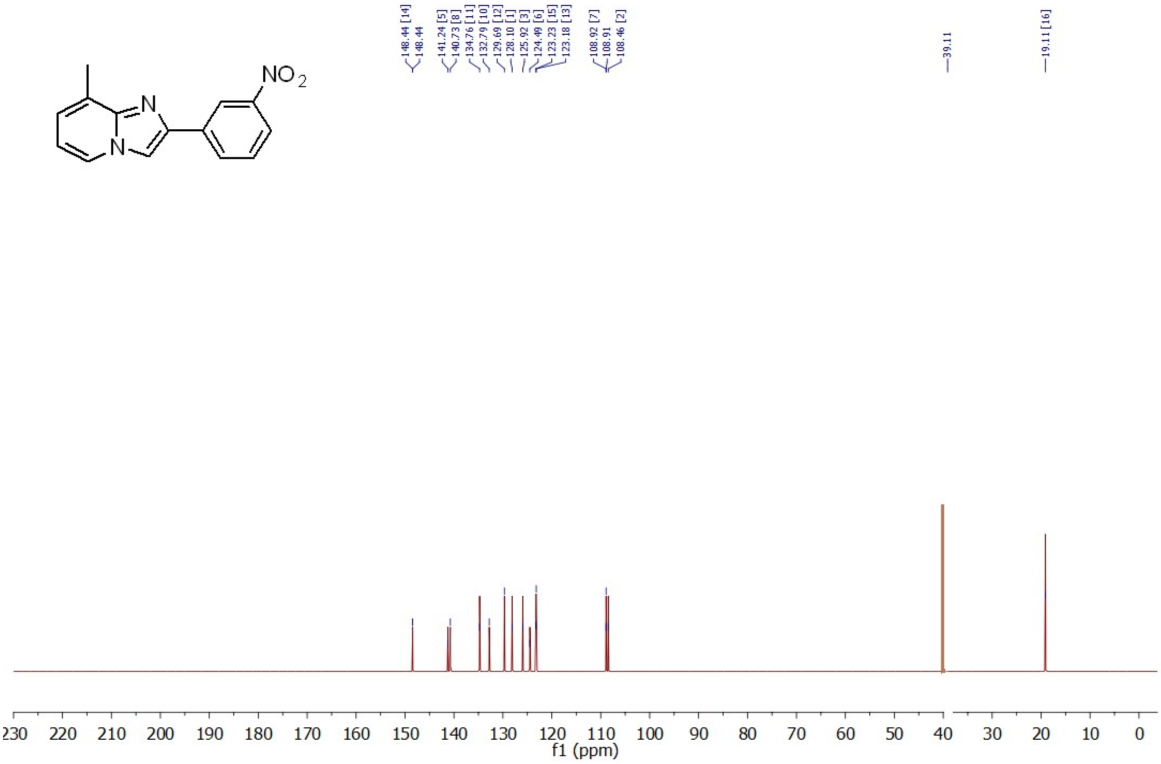

[3.16] (^{1}H & ^{13}C NMR), of the 2-(4-Chlorophenyl)-8-methylimidazo[1,2-a]pyridine (3eb)

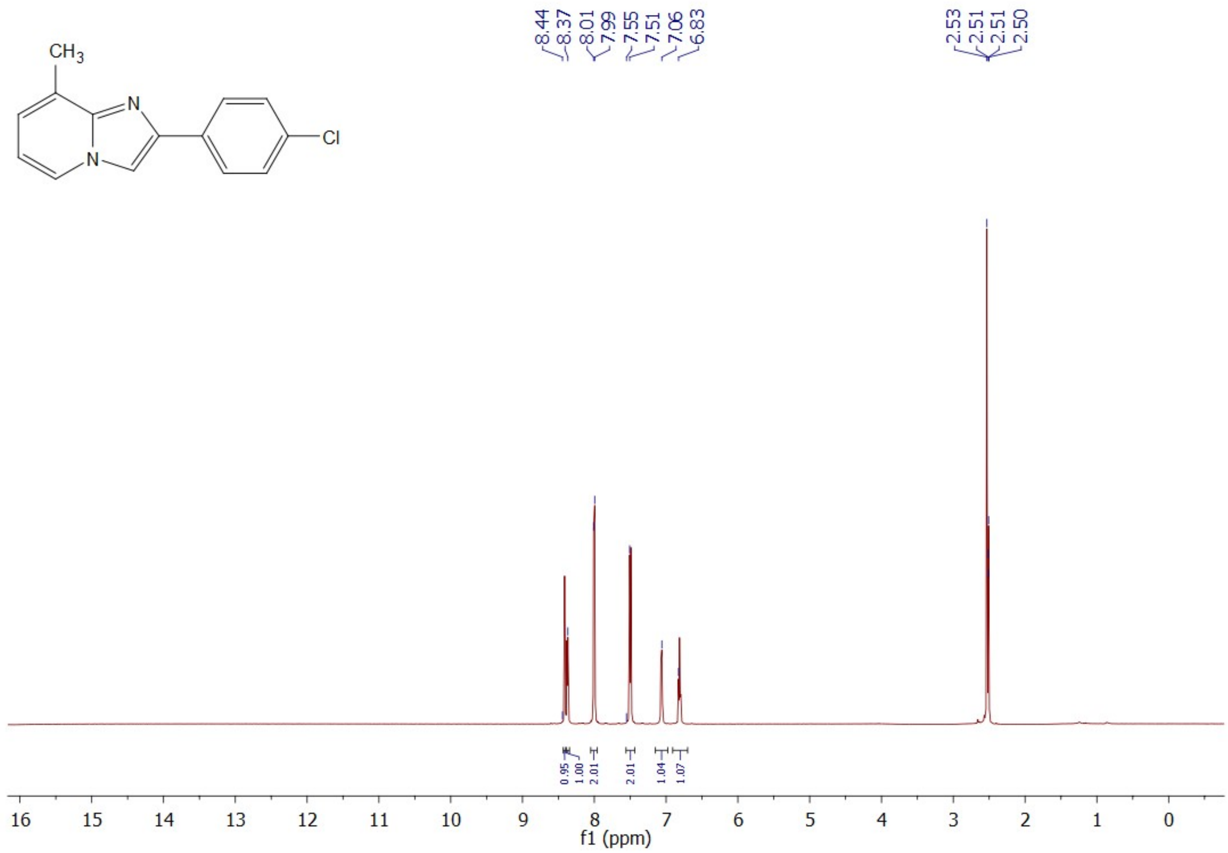

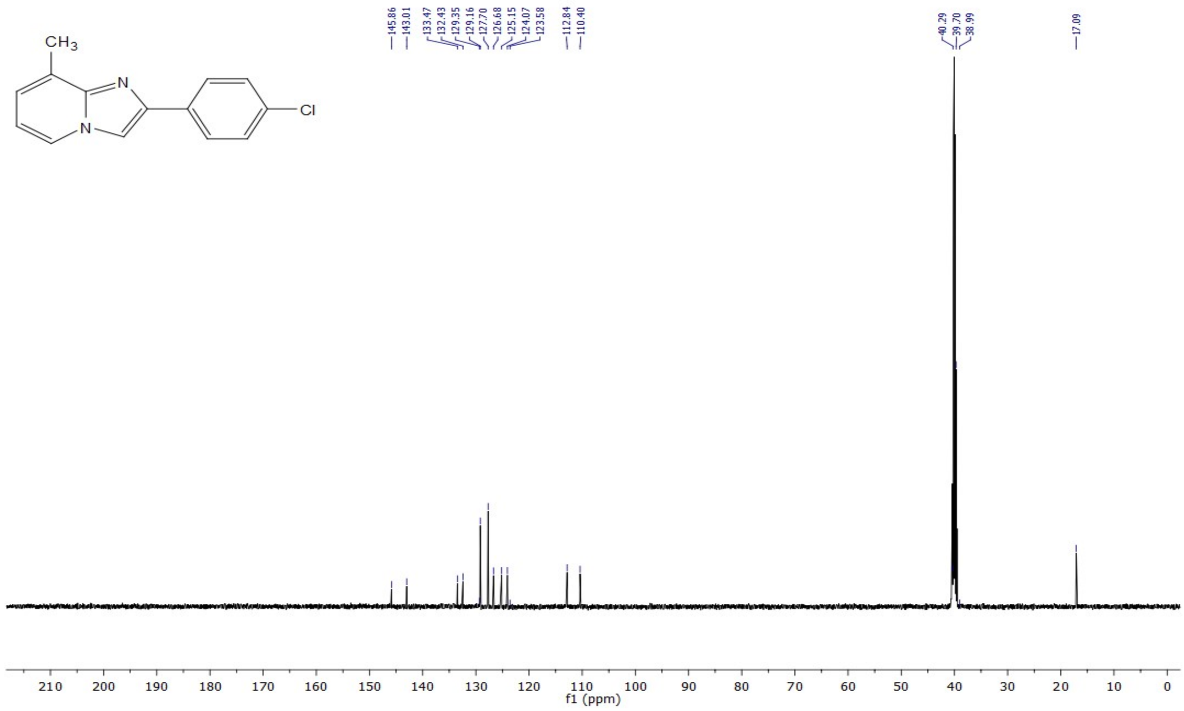

[3.17] (^{1}H & ^{13}C NMR), of the 2-(4-Bromophenyl)-8-methylimidazo[1,2-a]pyridine (3fb)

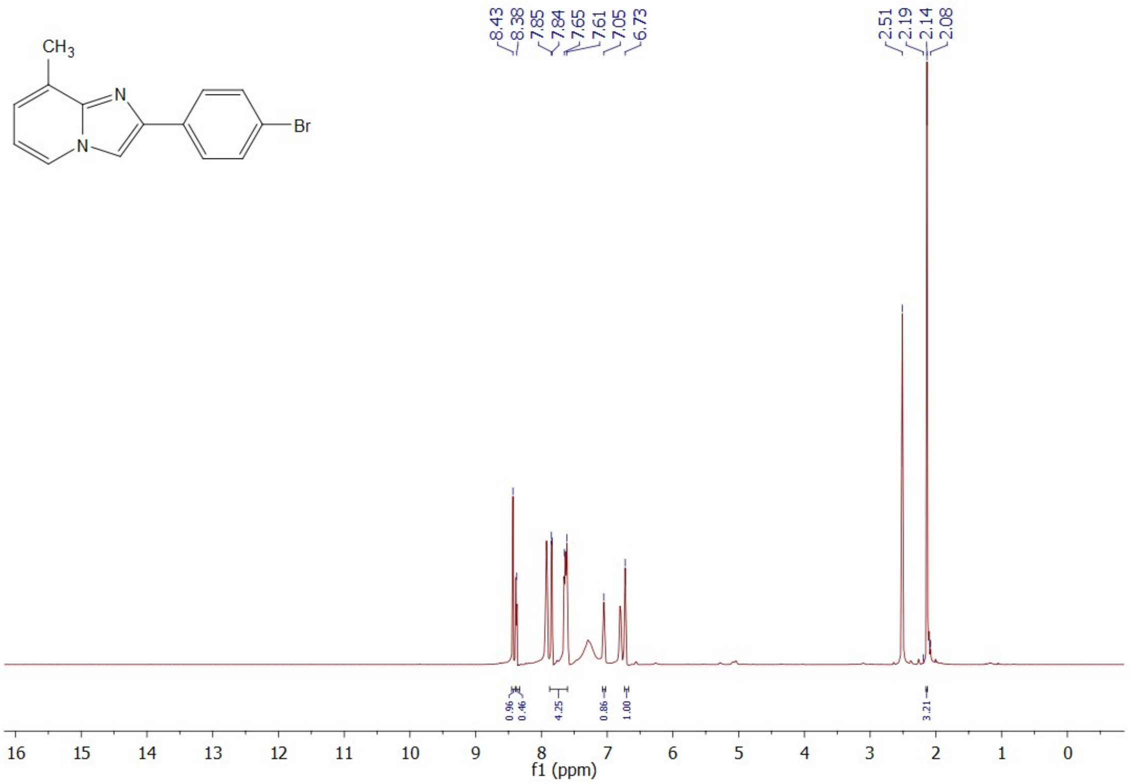

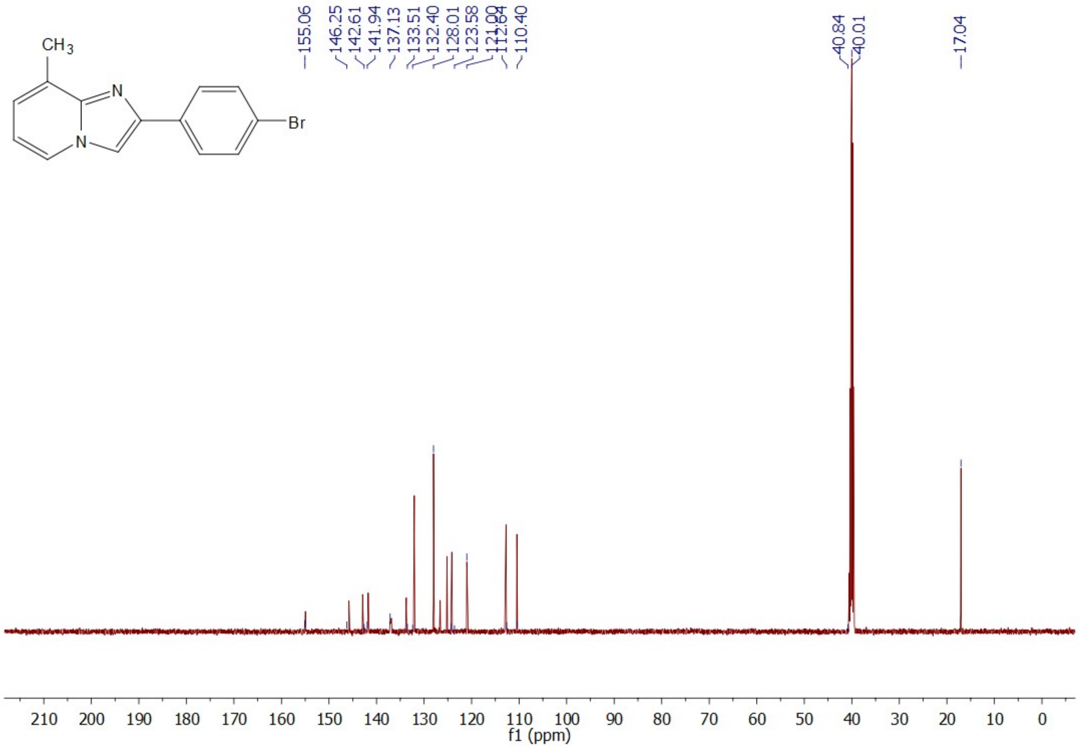

[3.18] ^{1}H&^{13}C NMR, of the 2-(-4-Flurophenyl)-8-methylimidazo[1,2-a]pyridine (3gb)

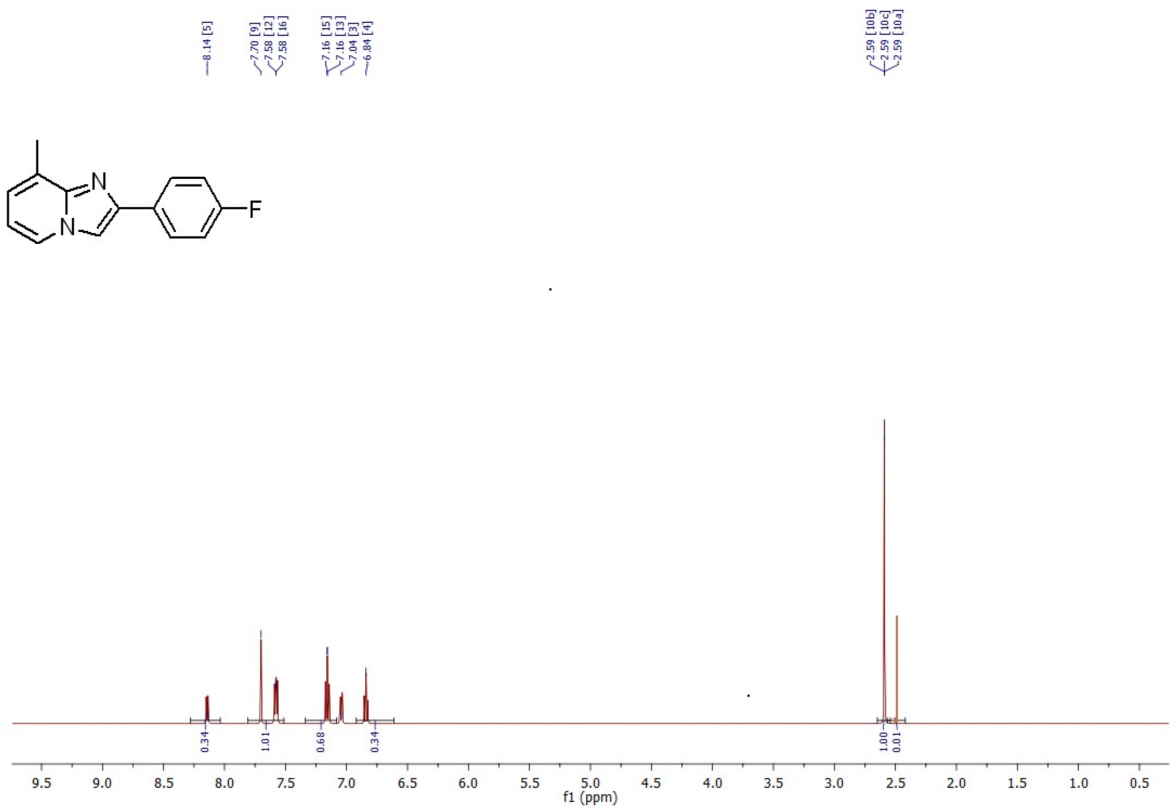

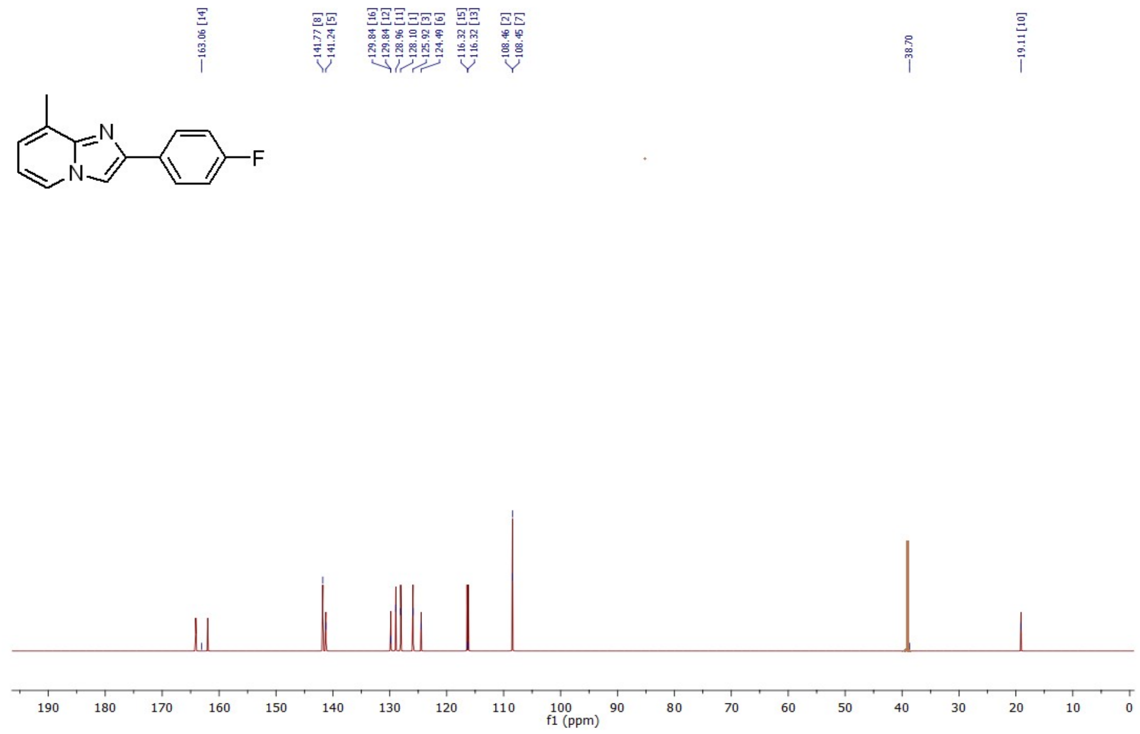

[3.18] HRMS & ^{19}F NMR of the 2-(4-Fluorophenyl) imidazo[1,2-a] pyridine(3gb)

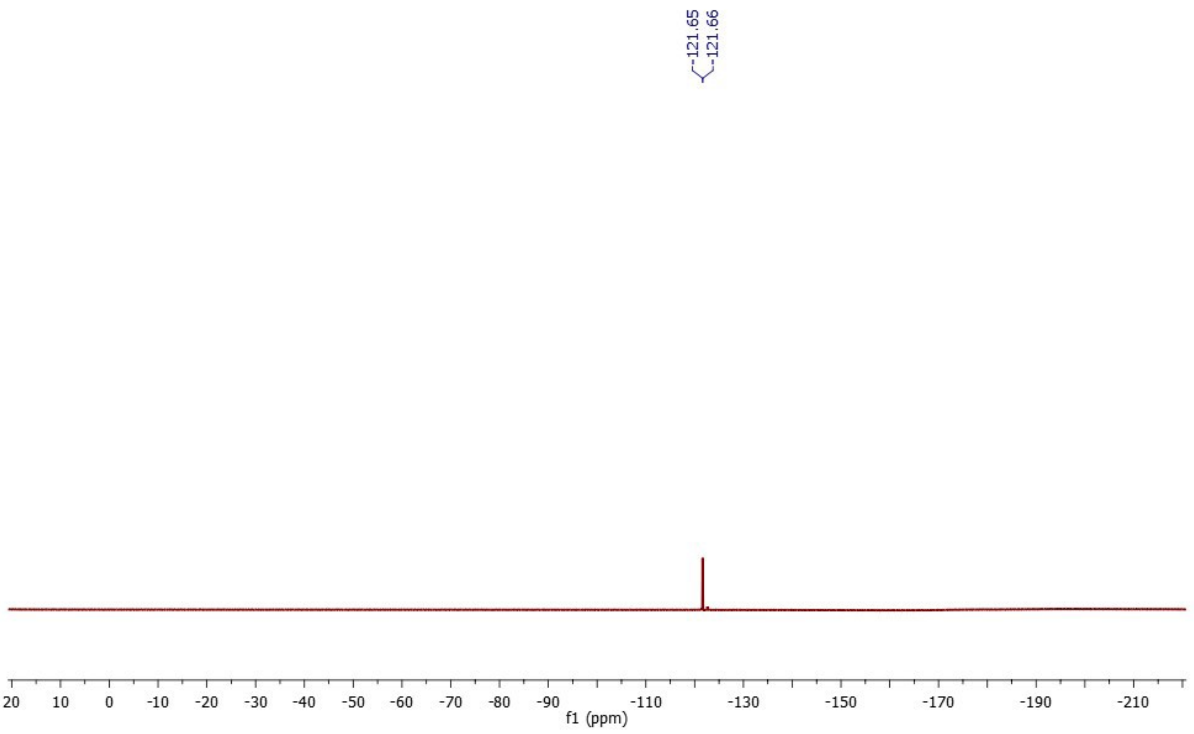

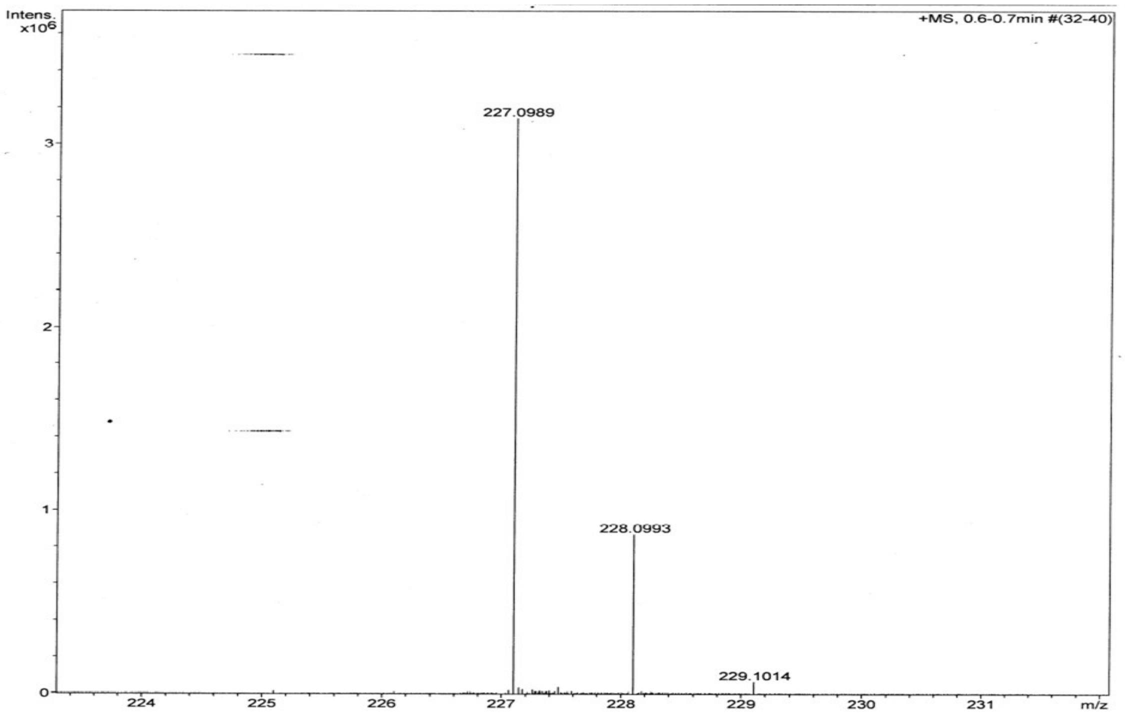

[3.19] (^{1}H & ^{13}C NMR) of the,2-(8-Methylimidazo[1,2-a]pyridin-2-yl) phenol, (3hb) :

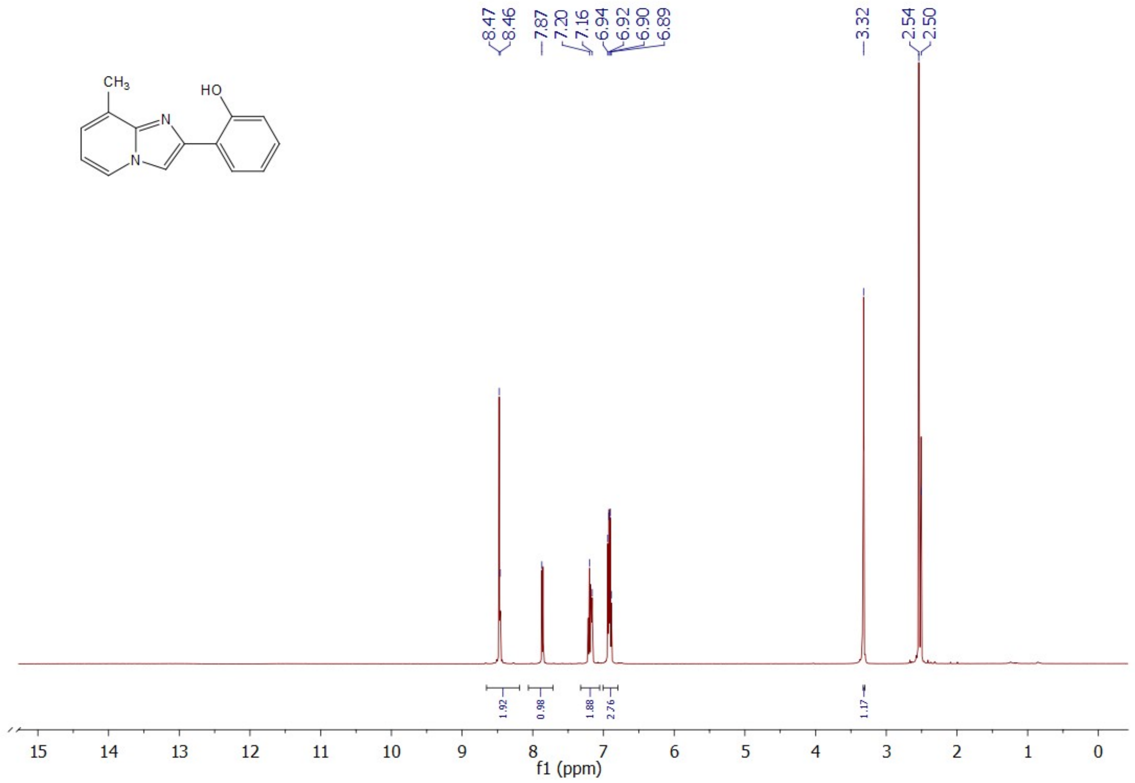

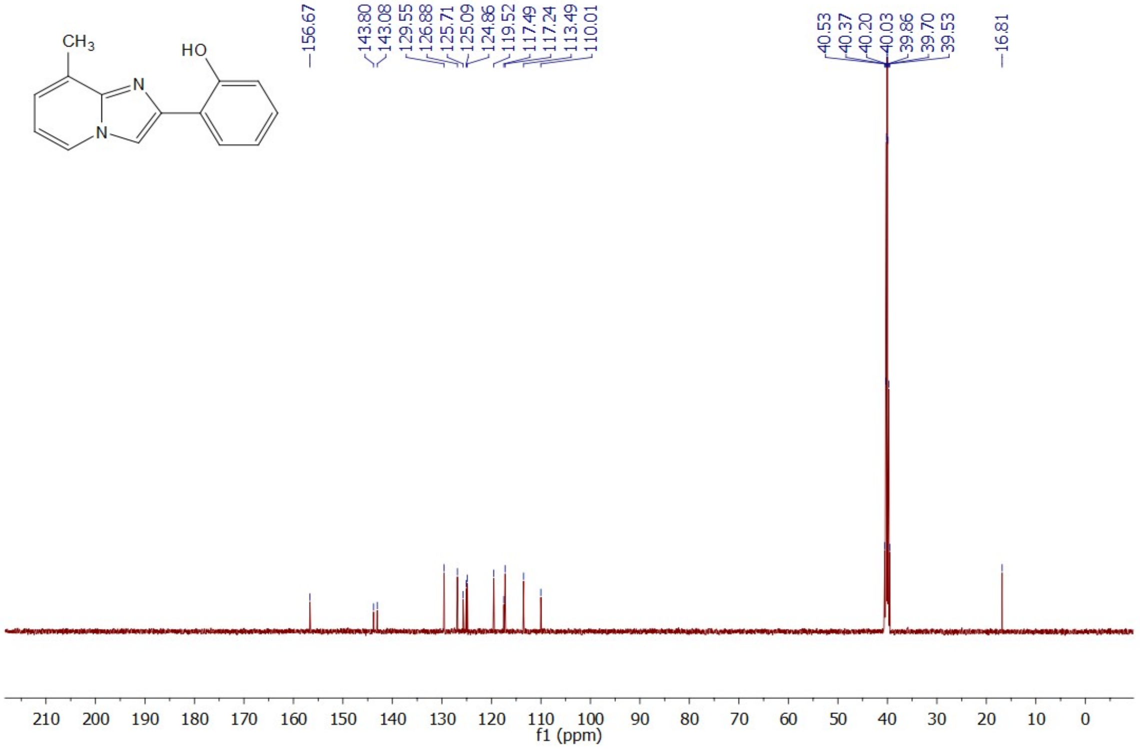

[3.20], (^{1}H &^{13}C NMR) of the [4-(8-Methylimidazo[1,2-a] pyridin-2-yl)phenol (3ib)

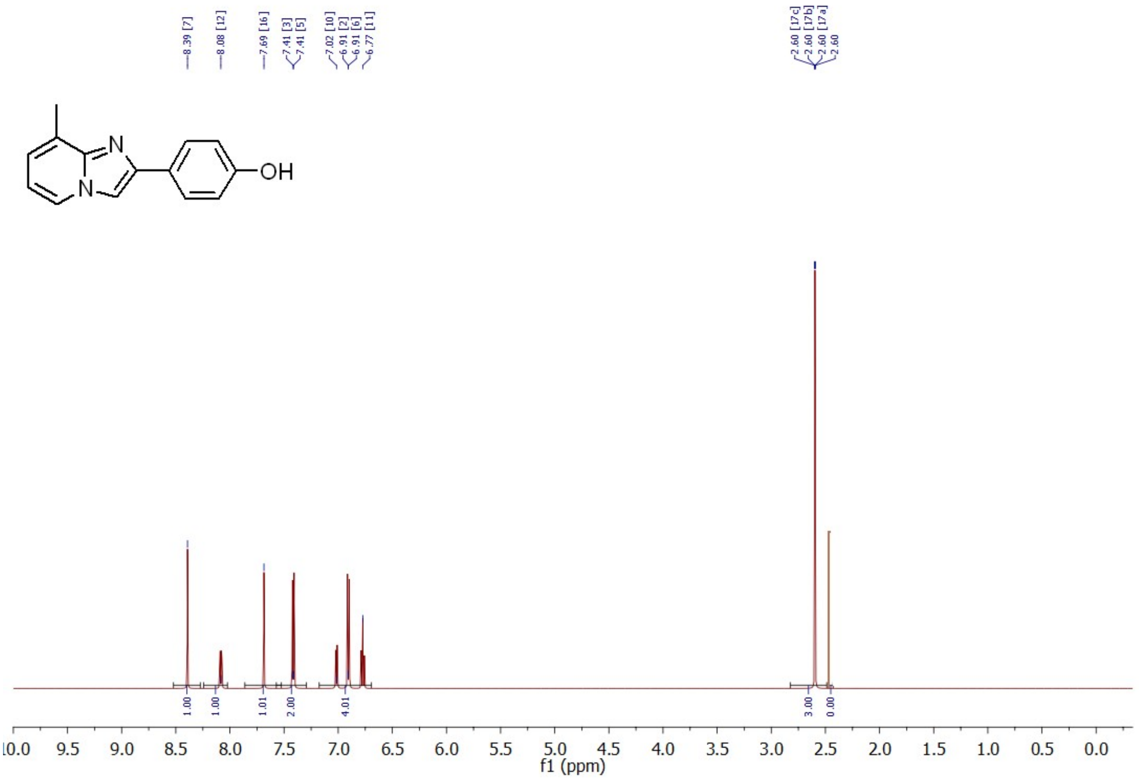

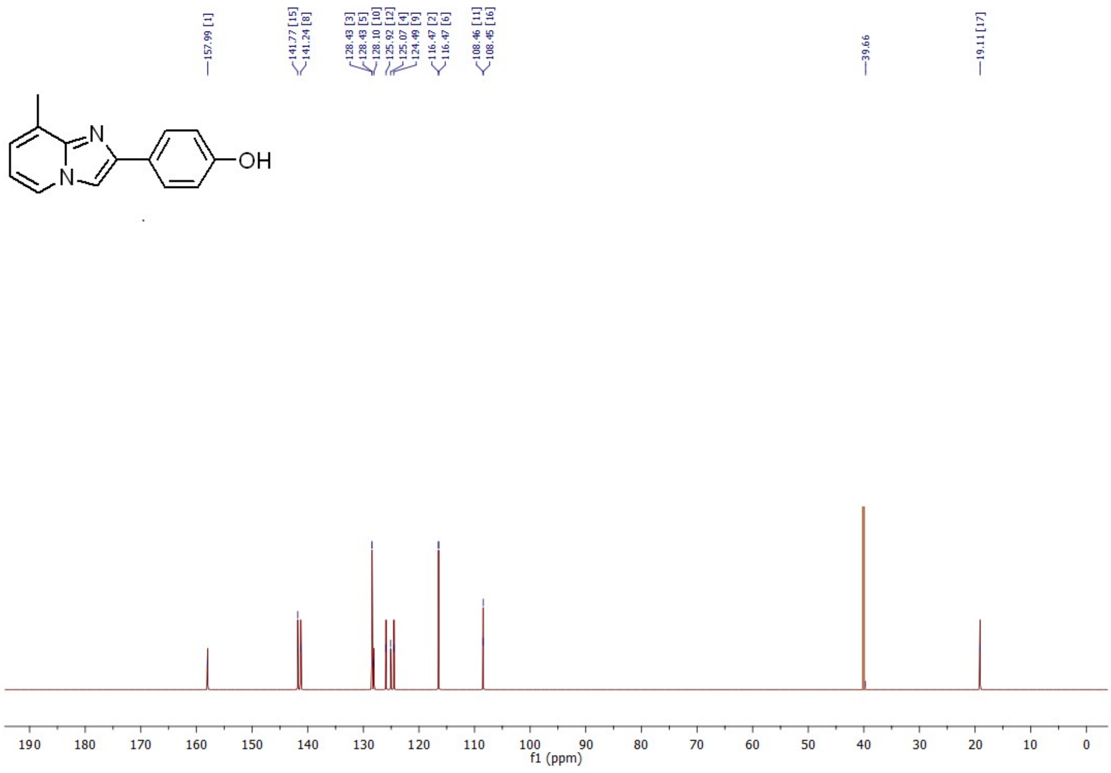

[3.21], (^{1}H and ^{13}C NMR) of the 6-Chloro-2-phenylimidazo[1,2-a]pyridine, (3ac):

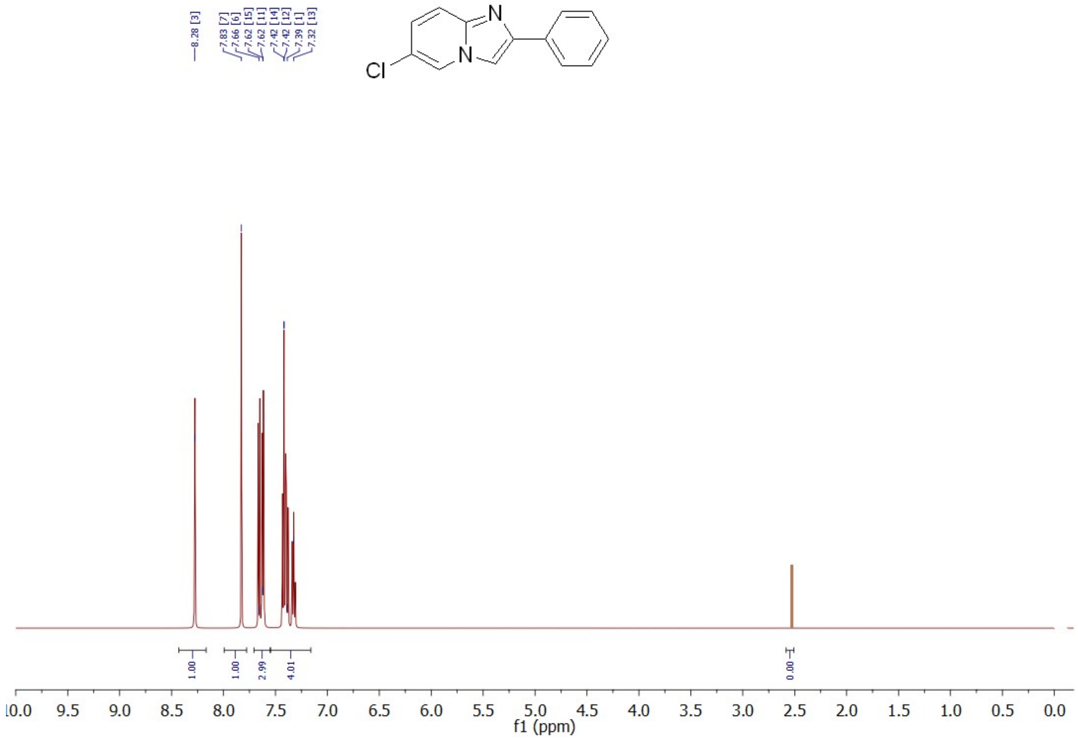

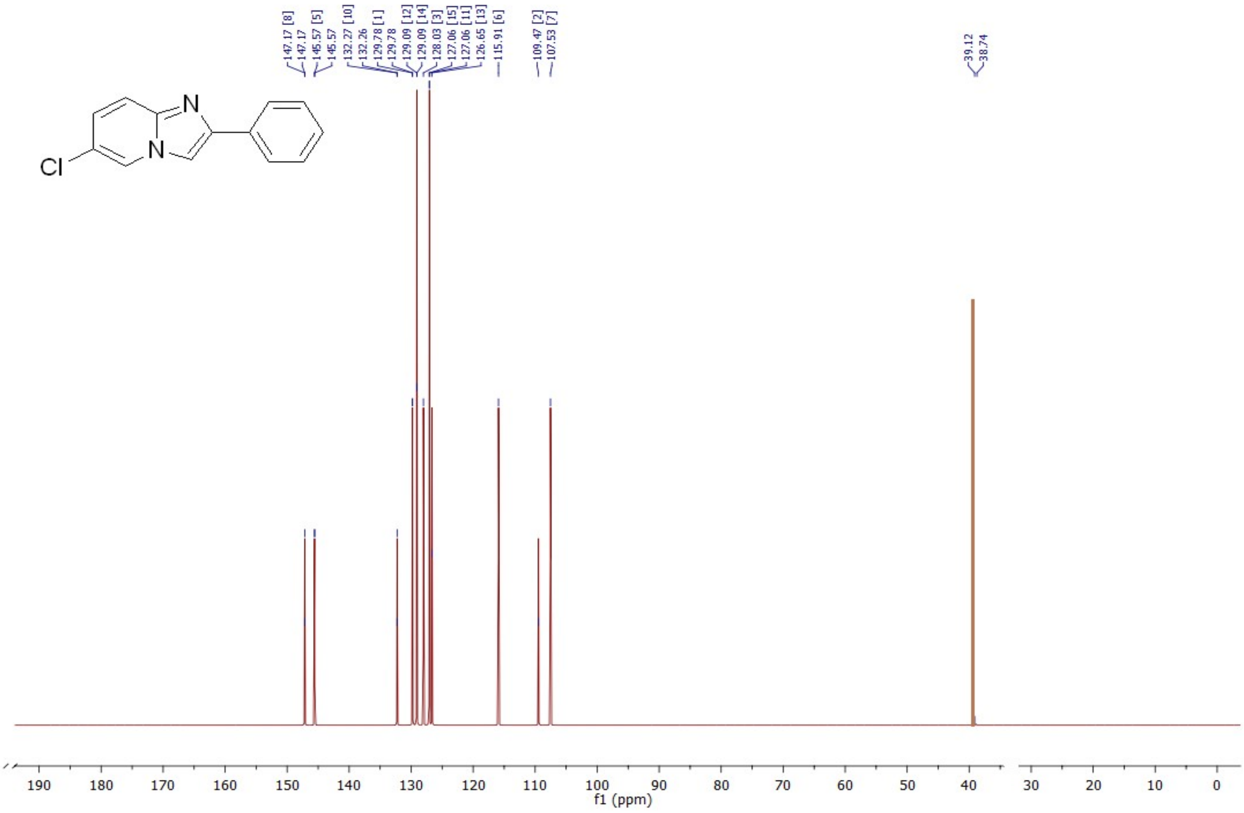

[3.22], ¹H &¹³C of the NMR 2-(4-Bromophenyl)-6-chloroimidazo[1,2-a] pyridine, (3fc)

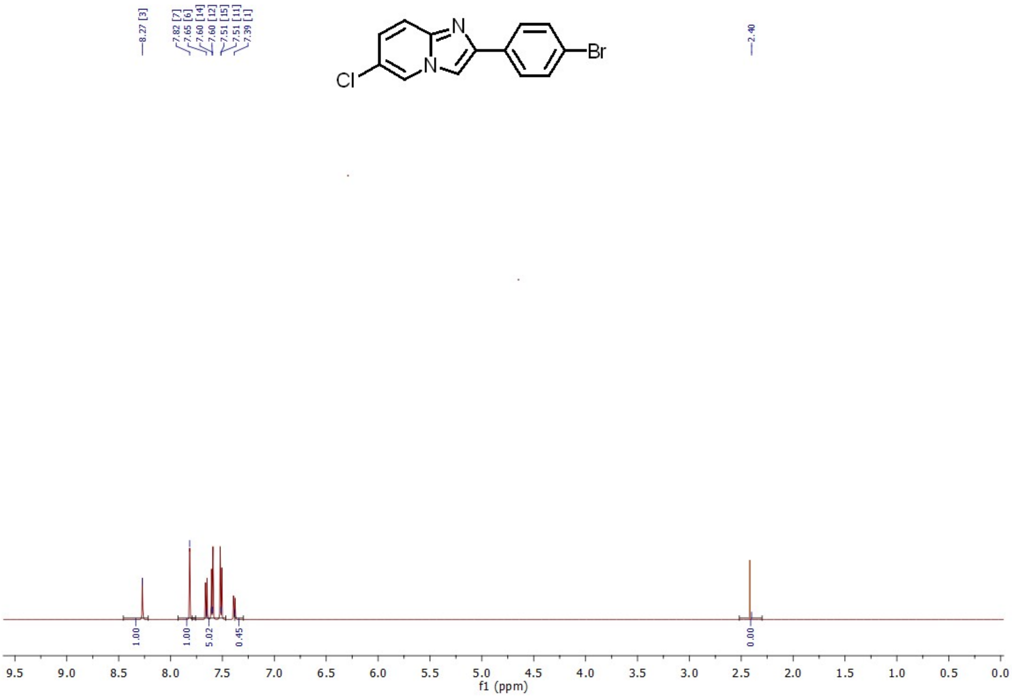

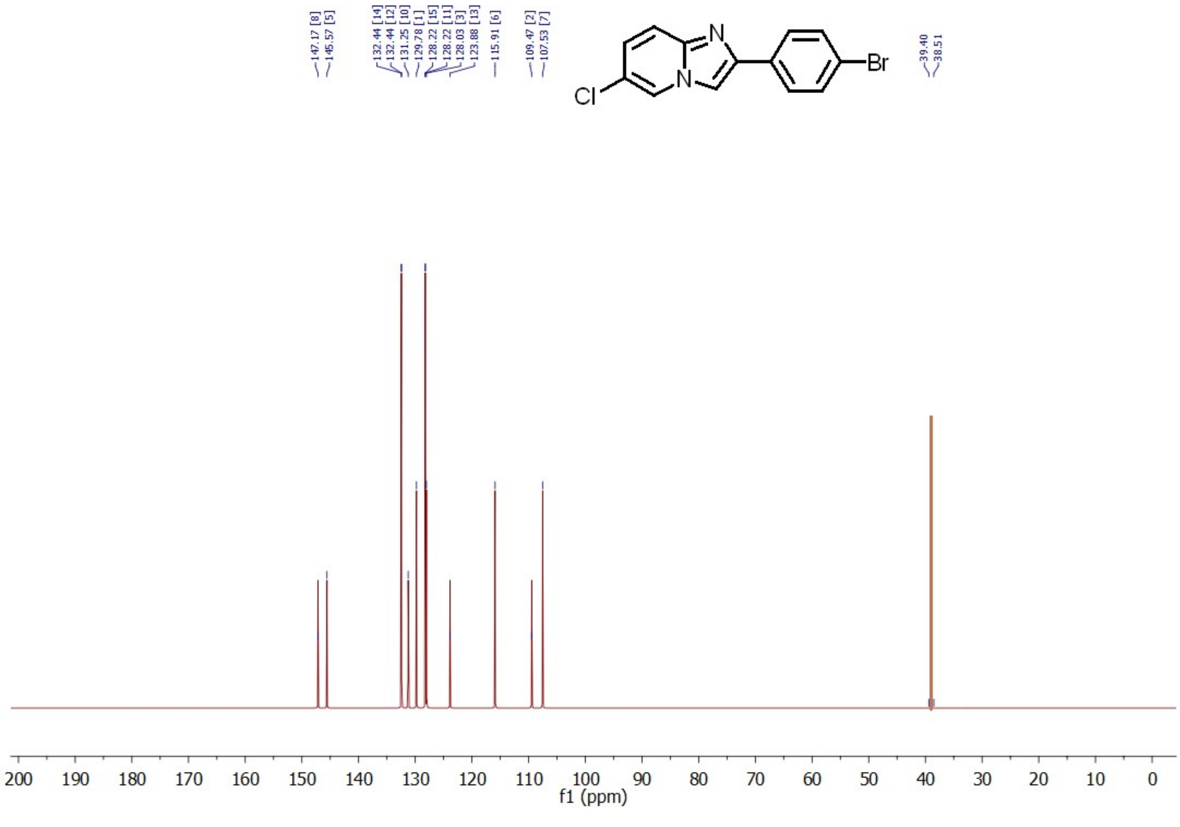

[3.23], (^{1}H and ^{13}C the of the NMR) 6-Chloro-2-(p-tolyl)imidazo[1,2-a]pyridine ,(3bc):

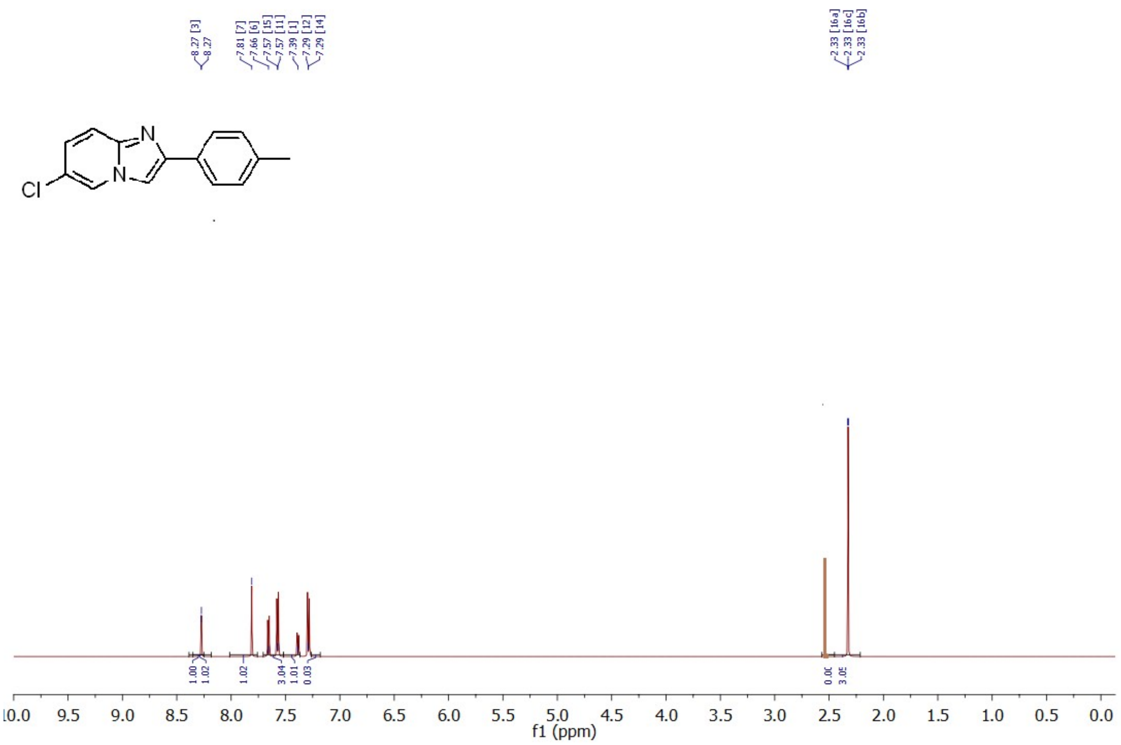

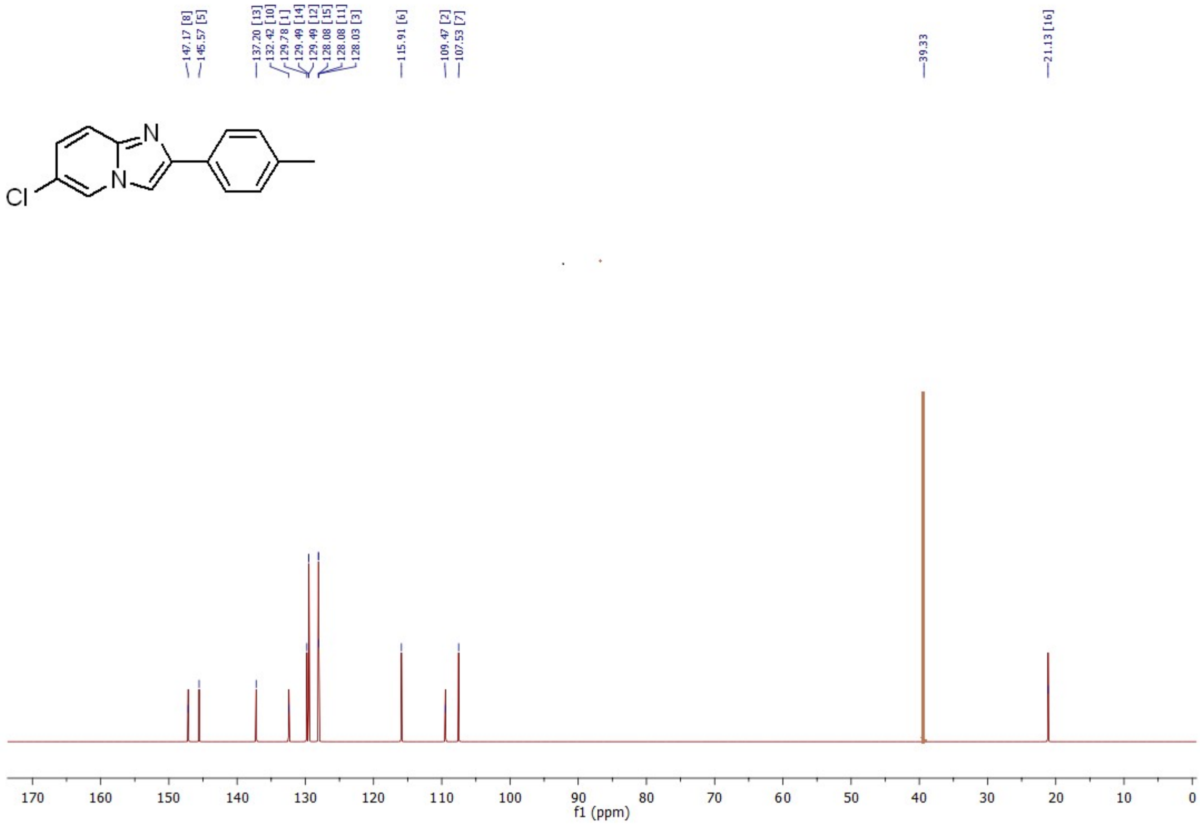

[3.24] (^{1}H and ^{13}C, of the NMR 6-Chloro-2-(4-chlorophenyl)imidazo[1,2a]pyridine(3ec):

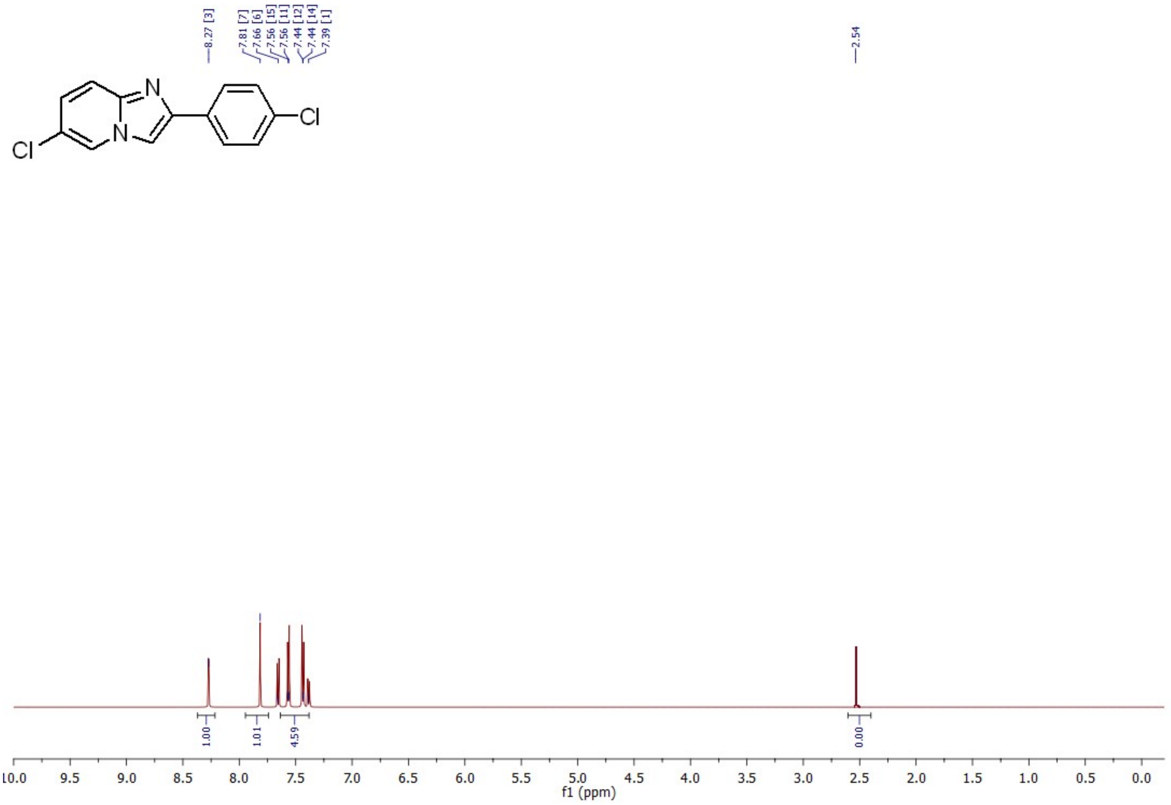

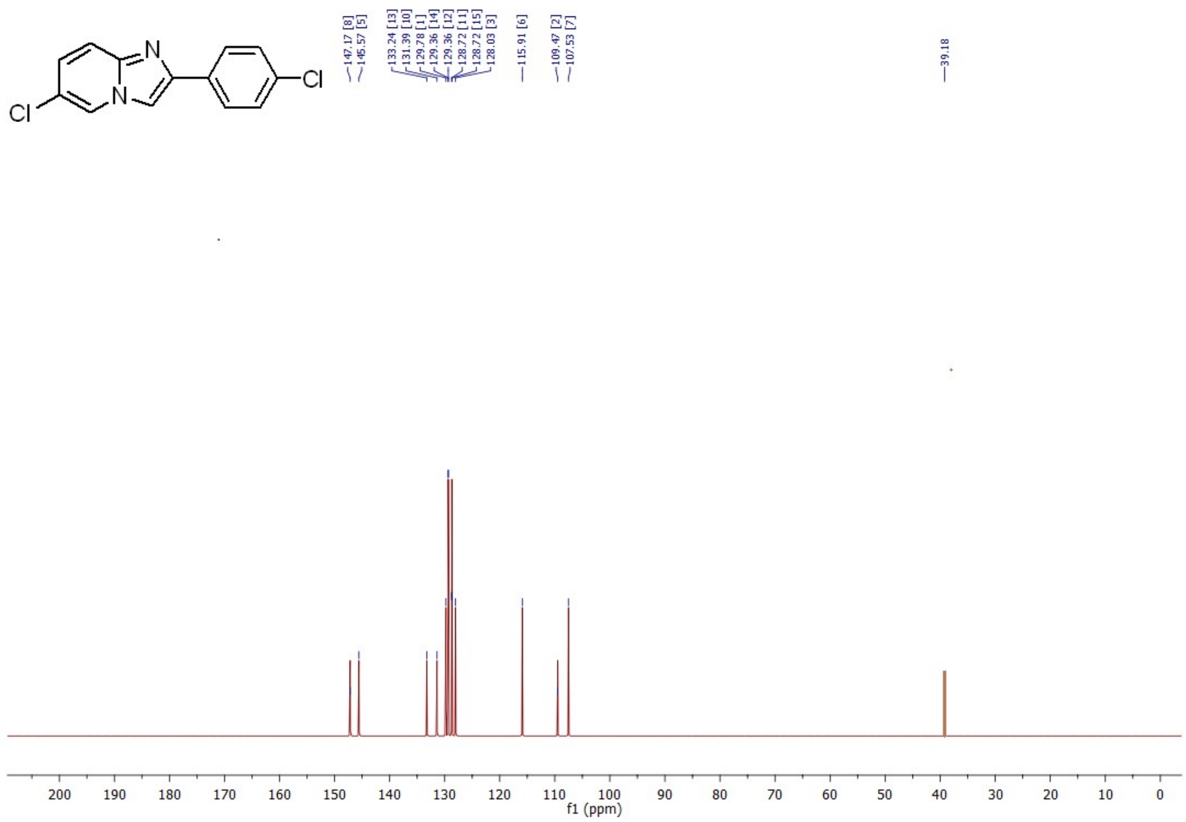

[3.25] ^{1}H and ^{13}C of the NMR,2-(6-Chloroimidazo[1,2-a]pyridin-2-yl)phenol (3hc):

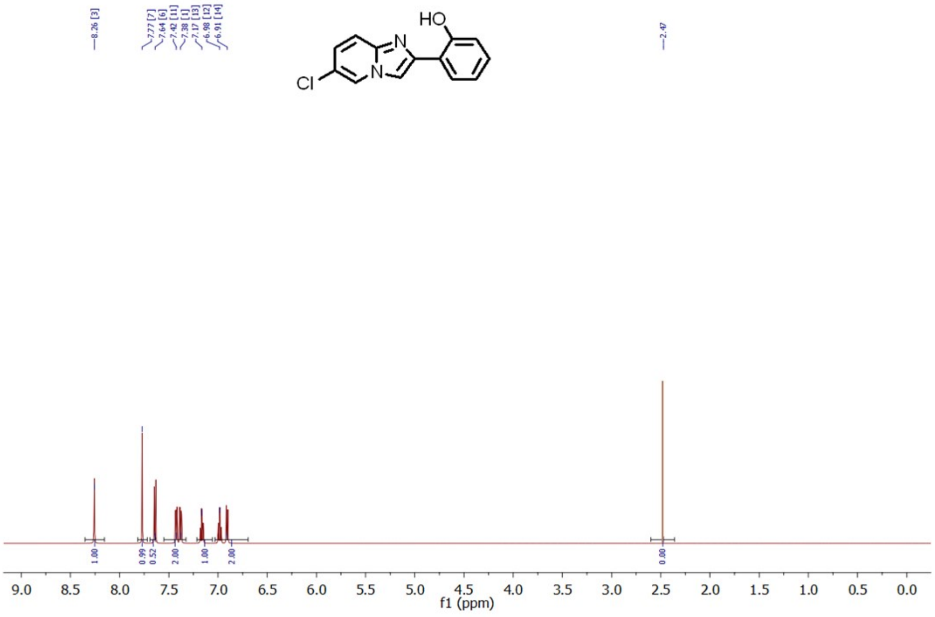

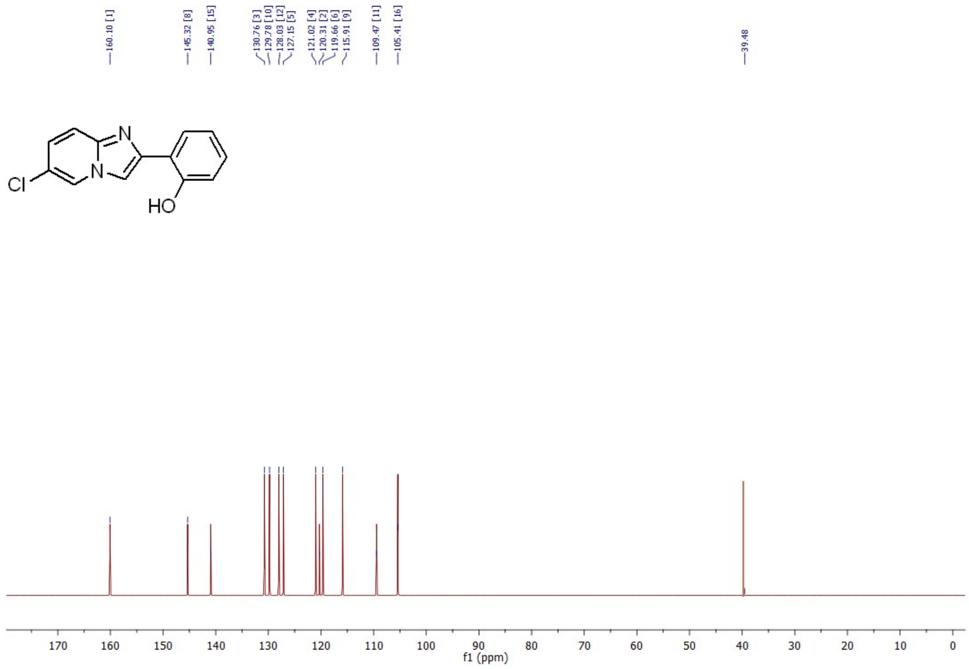

[3.26] (^{1}H and ^{13}C NMR) of the (Phenoxymethyl)-2-phenylimidazo[1,2-a]pyridine (3ad)

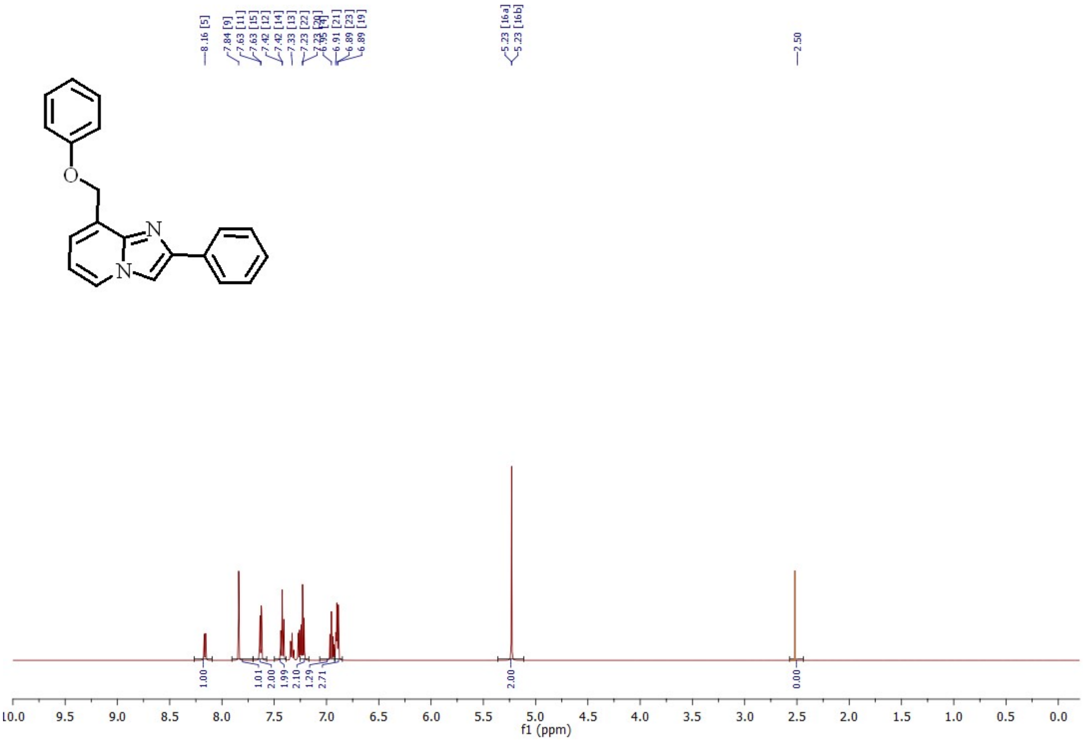

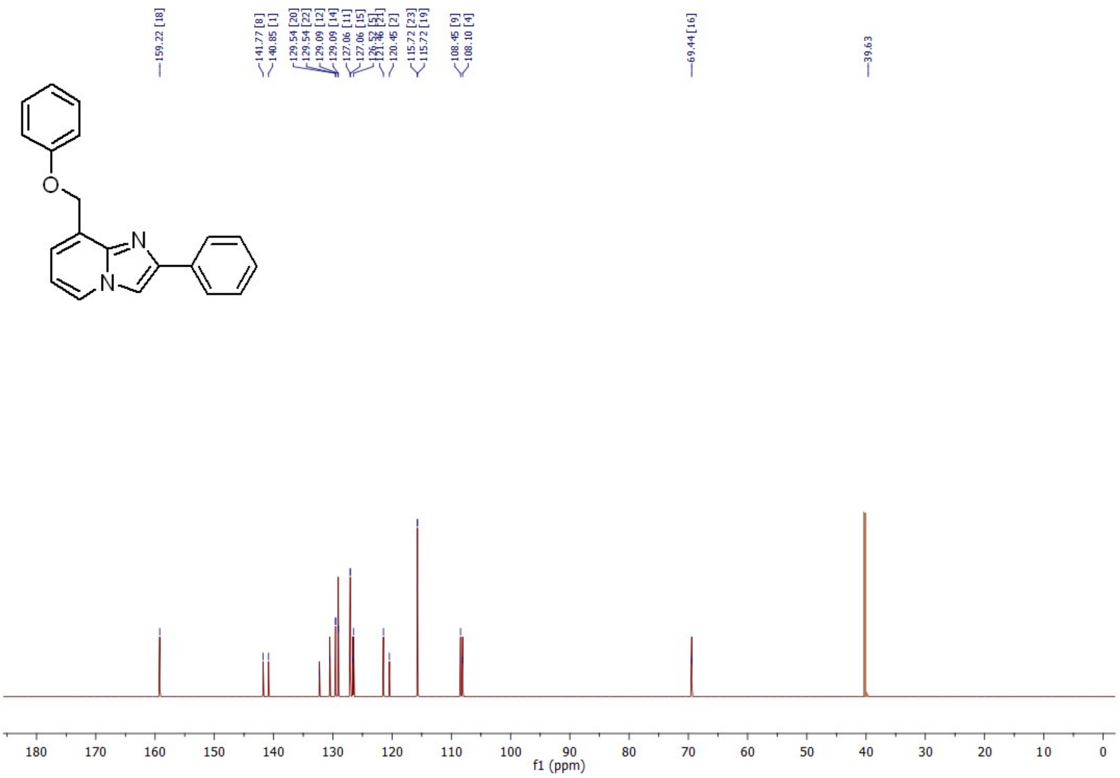

[3.27] ^{1}H and $^{13}CNMR$ of the 2-methylimidazo[1,2-a] pyridine of (3ae)

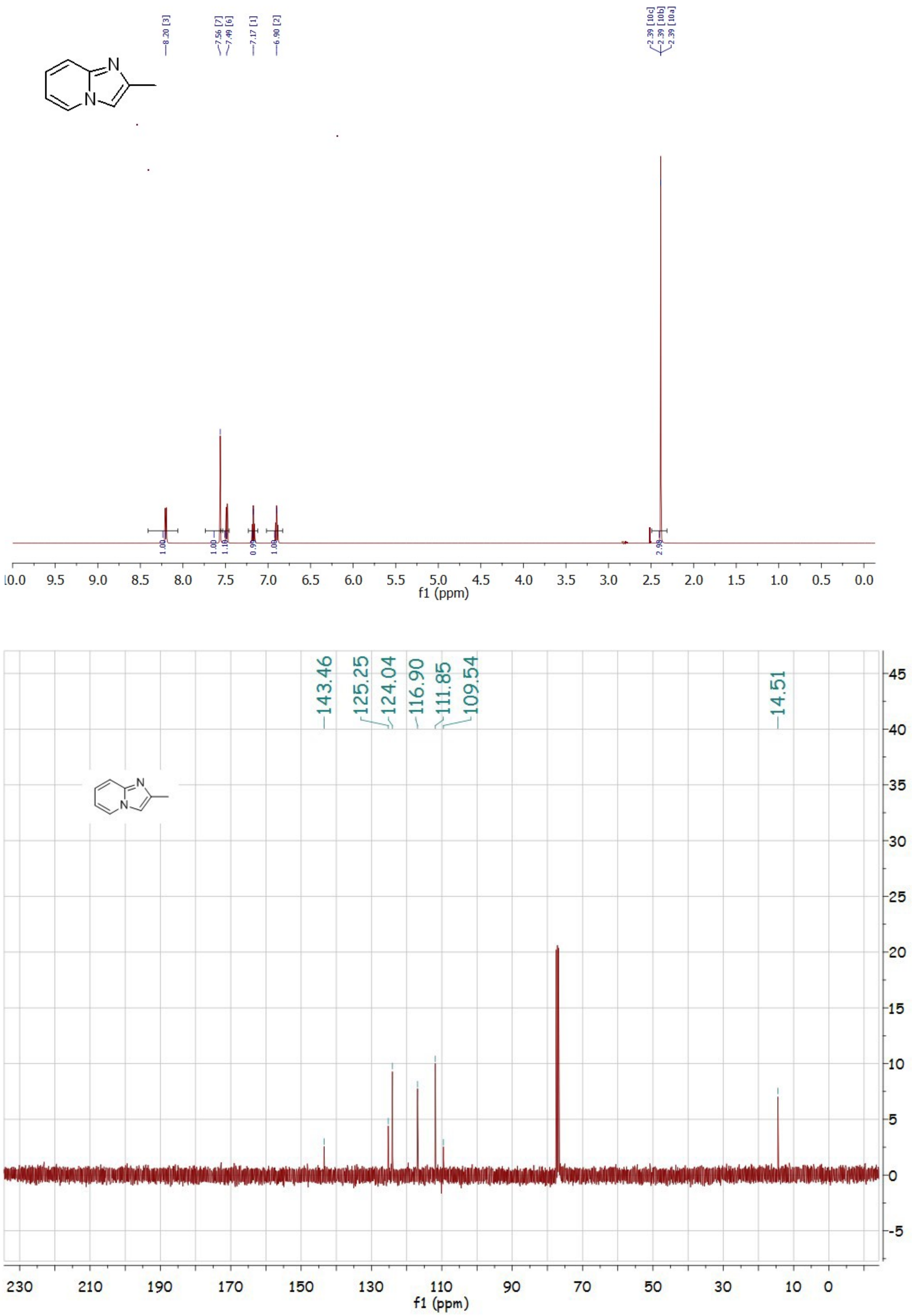

[3.28] ^{1}H and ^{13}CNMR of the 2-methylimidazo[1,2-a] pyridine of (3be)

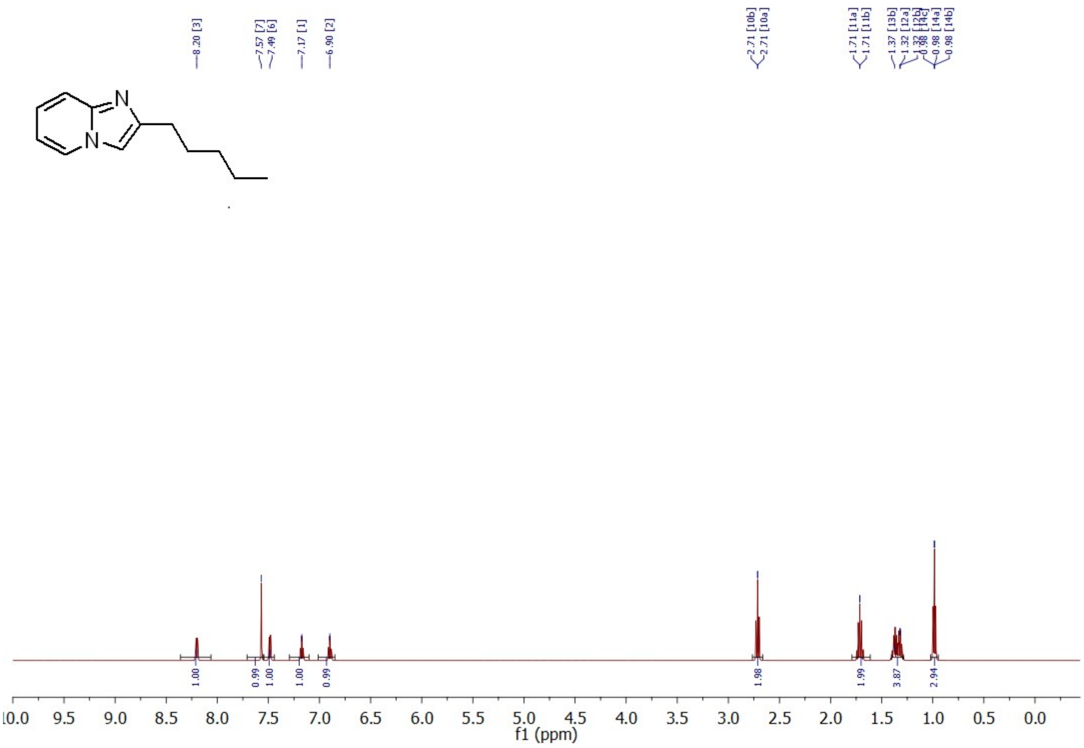

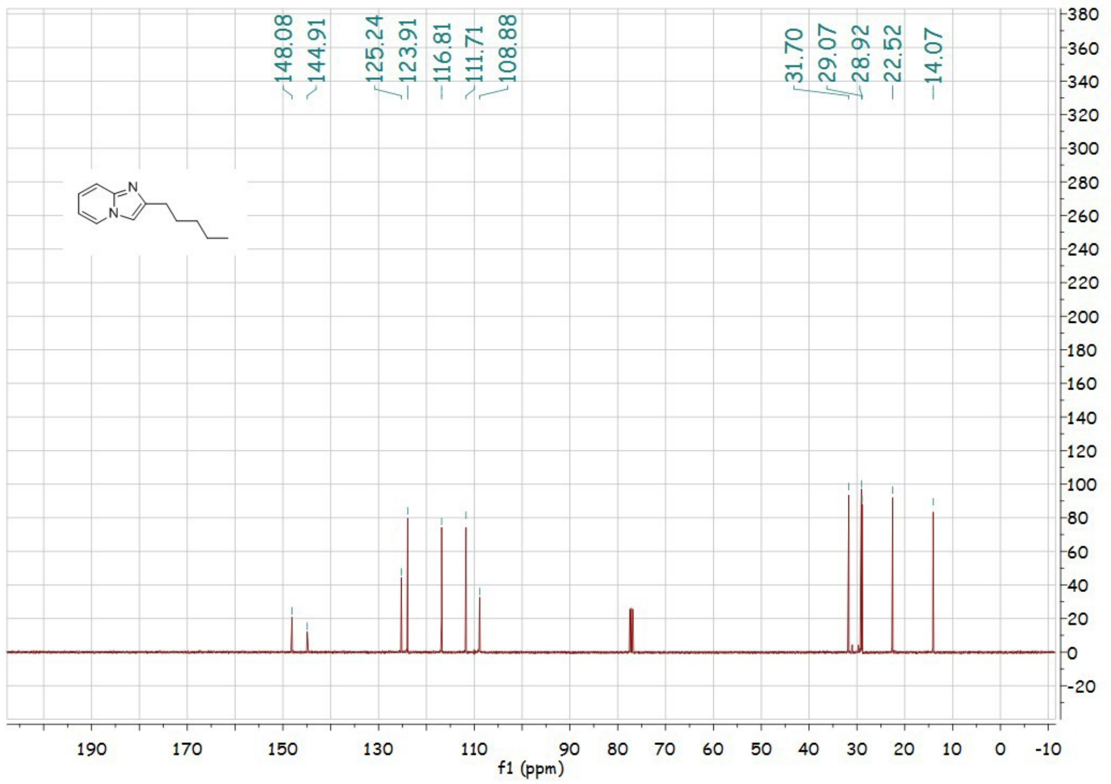

[3.29] ^{1}H and ^{13}C NMR of the 2-methylimidazo[1,2-a] pyridine of (3ce):

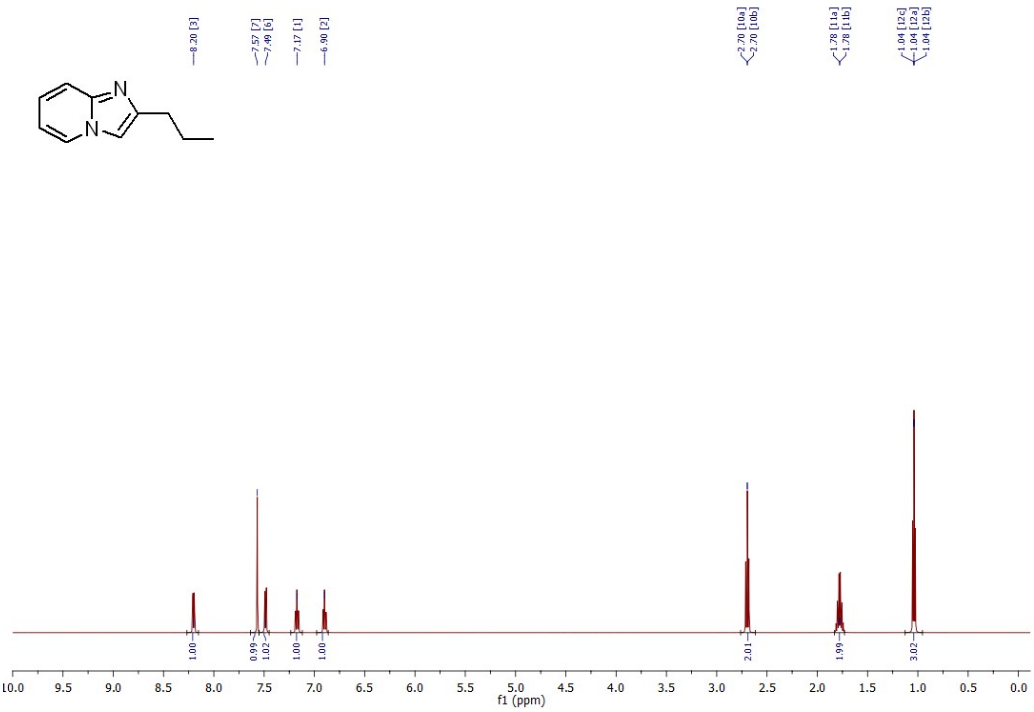

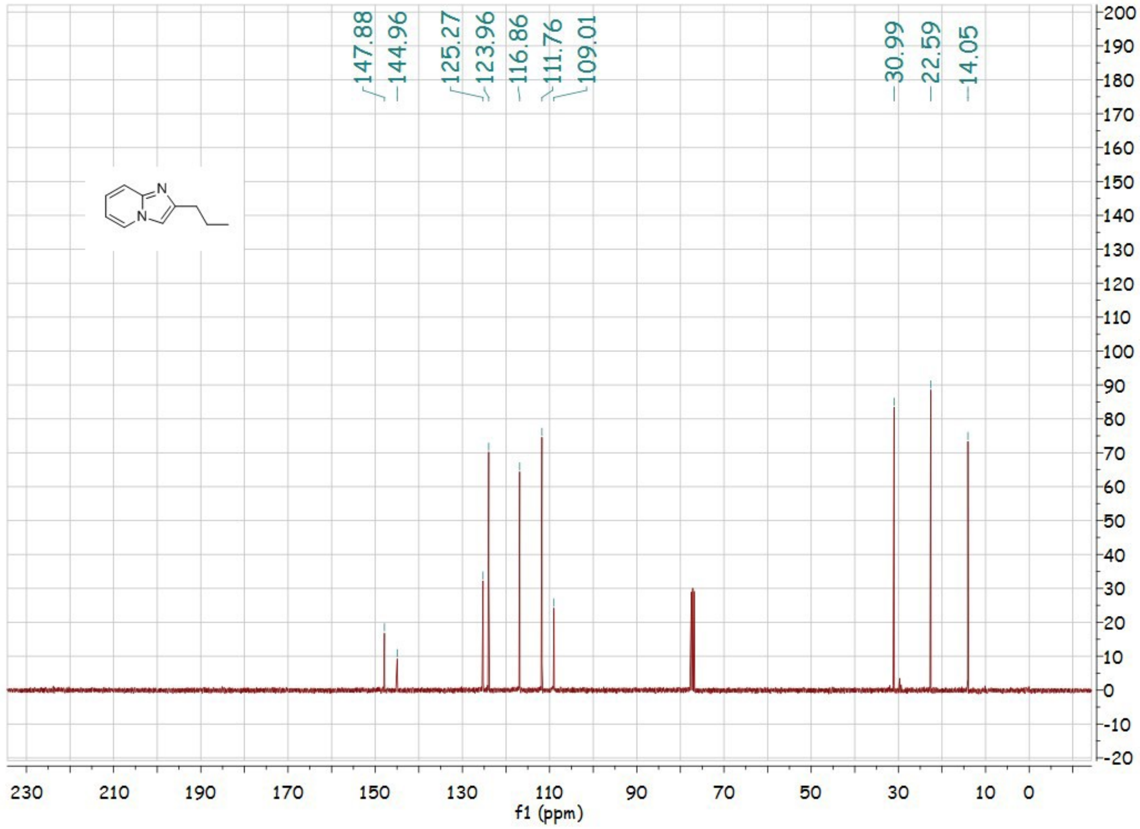

[3.29] ^{1}H and ^{13}CNMR of the 2-methylimidazo[1,2-a] pyridine of (3de)

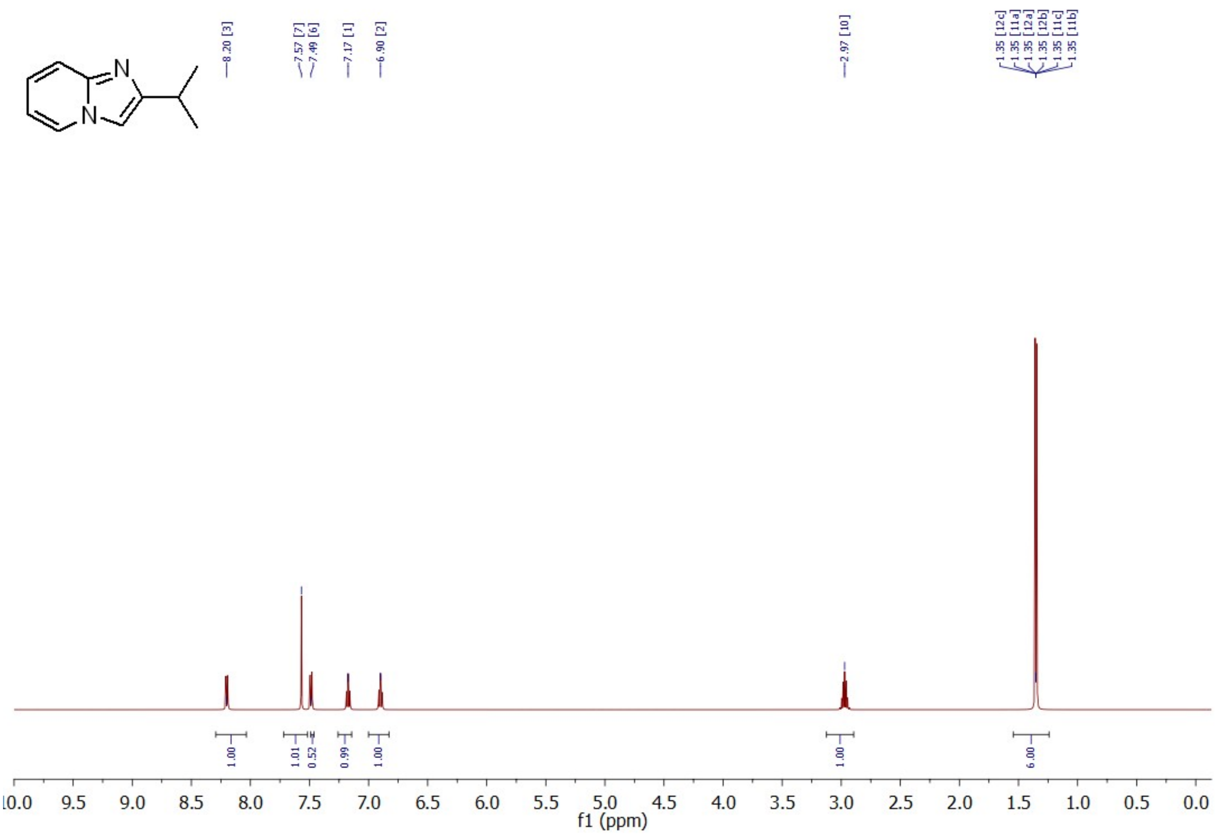

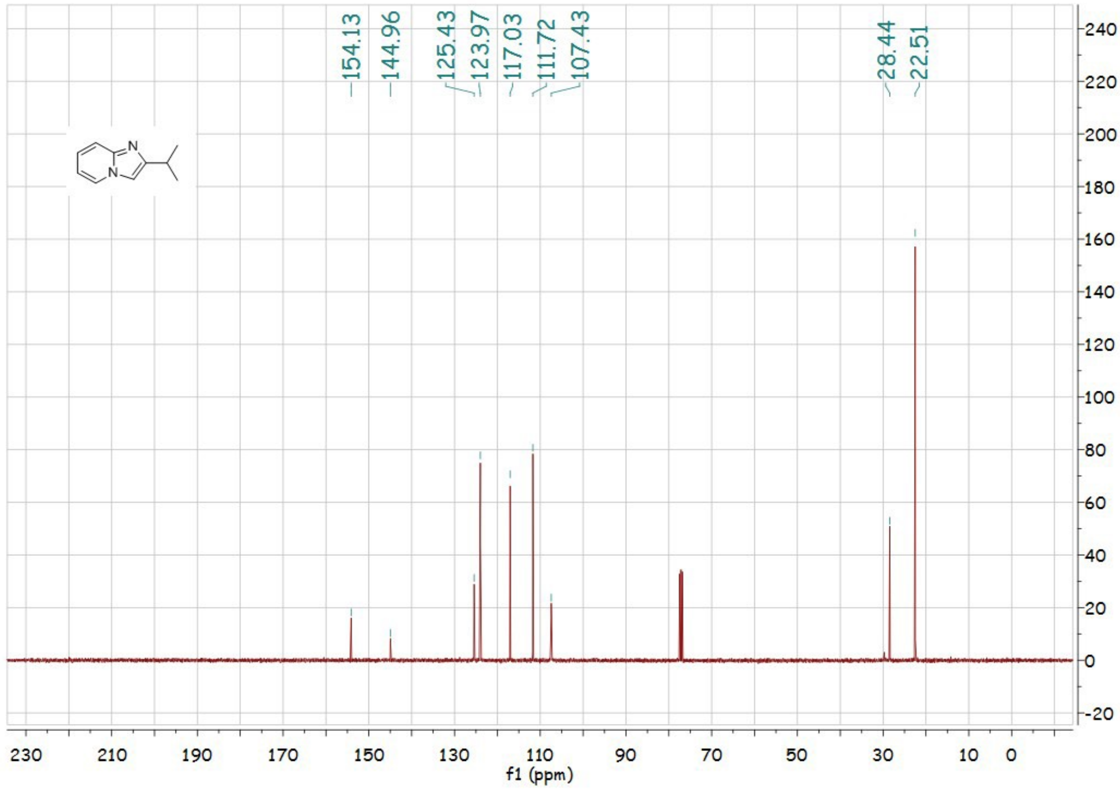

[3.1] HRMS of 2-phenylimidazo[1,2-a] pyridine(3aa) :

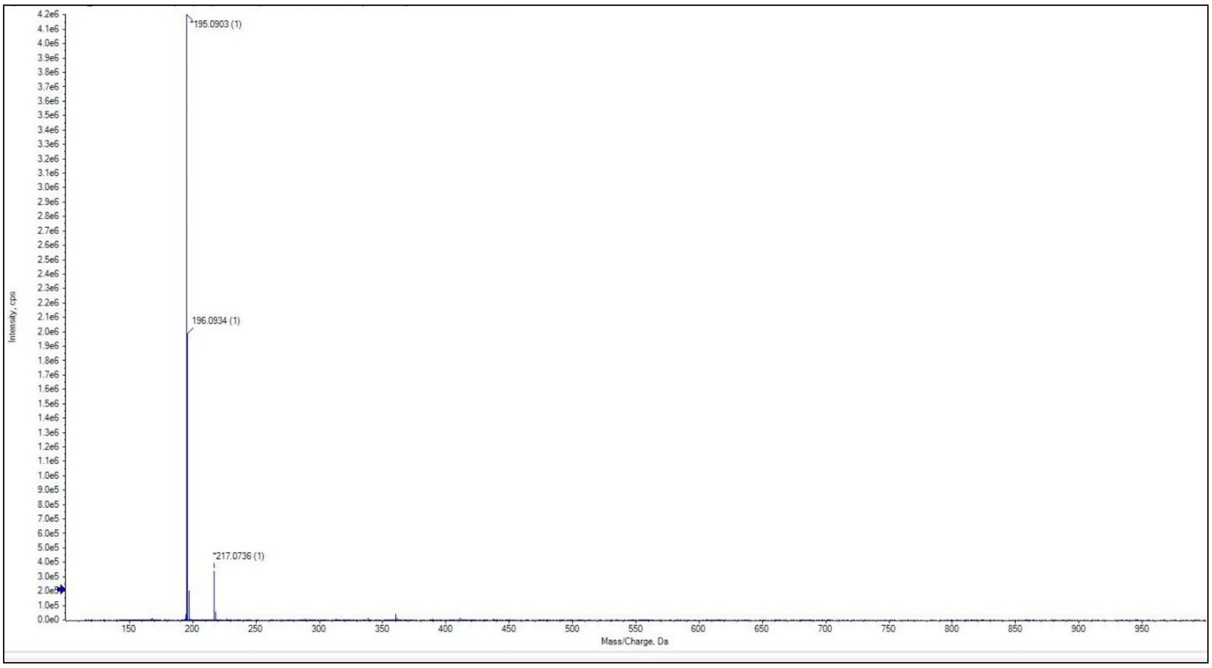

[3.1] HRMS of the 2-(p-Tolyl) imidazo [1,2-a] pyridine (3ba) :

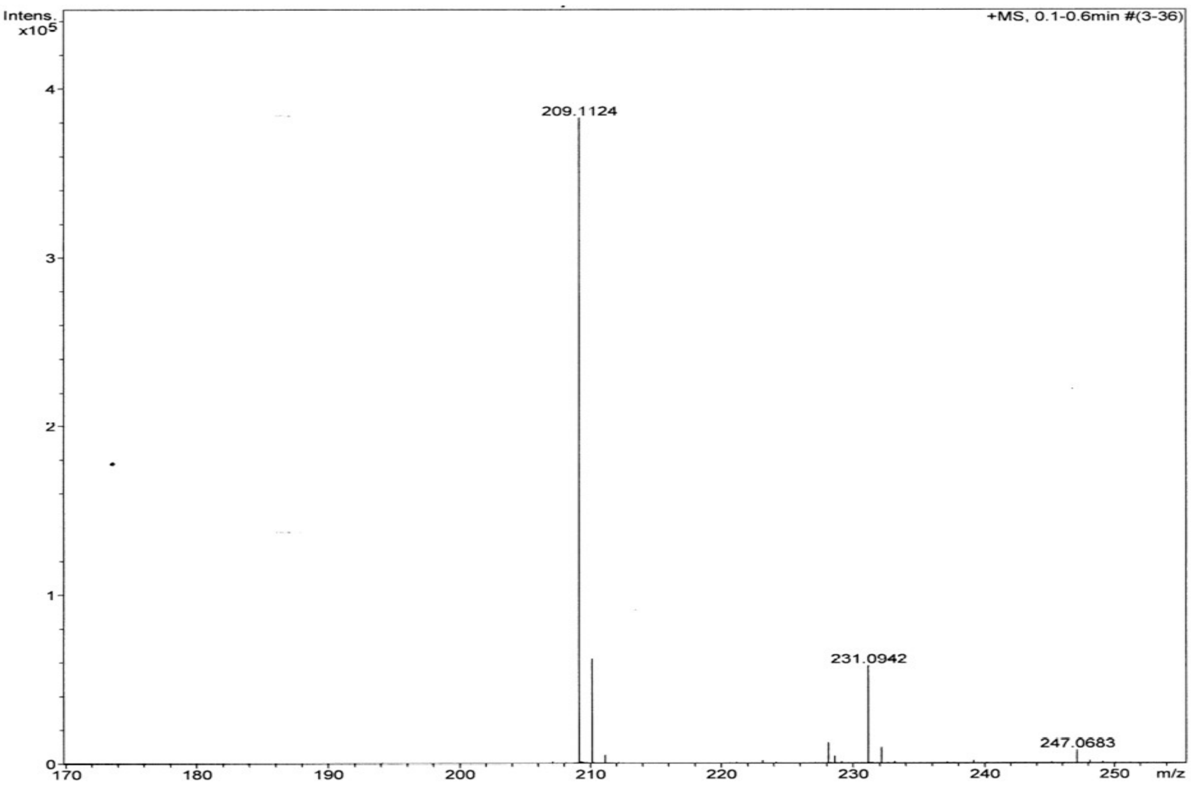

[3.1] HRMS of the 2-(4-Bromophenyl) imidazo[1,2-a] pyridine (3fa)

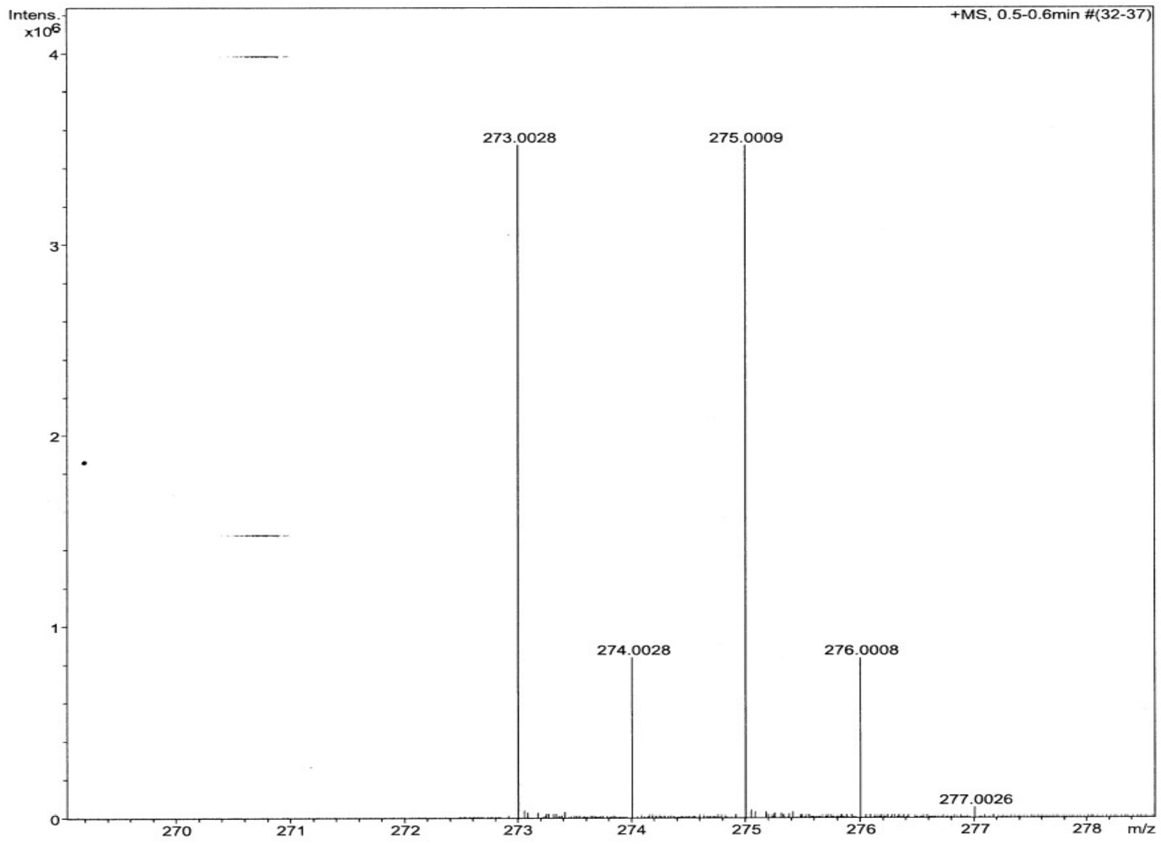

I.R.Spectra:(3aa)

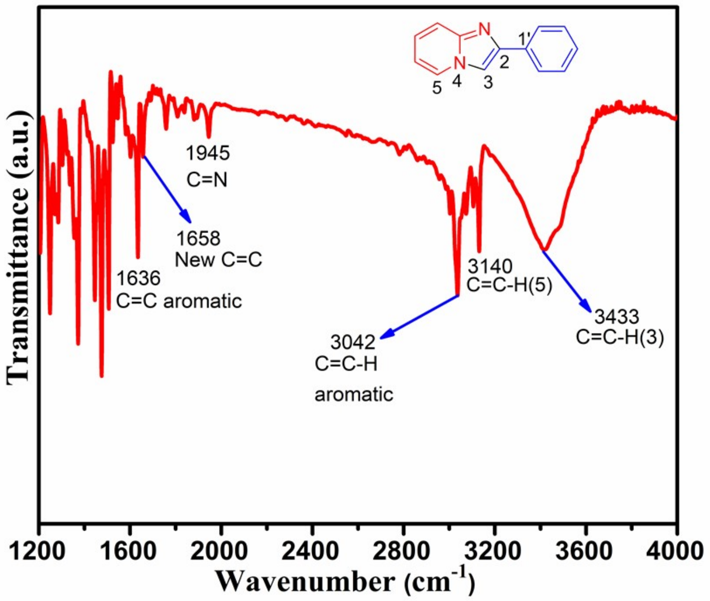

Supplement: RA-014-D3RA07145F-s001 [file RA-014-D3RA07145F-s001.pdf]
